# Sequence variants contributing to dysregulated inflammatory responses across keratoconic cone surface in adolescent patients with keratoconus

**DOI:** 10.3389/fimmu.2023.1197054

**Published:** 2023-07-06

**Authors:** Katarzyna Jaskiewicz, Magdalena Maleszka-Kurpiel, Michał Kabza, Justyna A. Karolak, Marzena Gajecka

**Affiliations:** ^1^ Institute of Human Genetics, Polish Academy of Sciences, Poznan, Poland; ^2^ Optegra Eye Health Care Clinic in Poznan, Poznan, Poland; ^3^ Chair of Ophthalmology and Optometry, Poznan University of Medical Sciences, Poznan, Poland; ^4^ Chair and Department of Genetics and Pharmaceutical Microbiology, Poznan University of Medical Sciences, Poznan, Poland

**Keywords:** cornea, corneal epithelium, inflammation, non-coding sequence variation, whole genome, WGS, keratoconus

## Abstract

**Background:**

Keratoconus (KTCN) is the most common corneal ectasia resulting in a conical shape of the cornea. Here, genomic variation in the corneal epithelium (CE) across the keratoconic cone surface in patients with KTCN and its relevance in the functioning of the immune system were assessed.

**Methods:**

Samples from four unrelated adolescent patients with KTCN and two control individuals were obtained during the CXL and PRK procedures, respectively. Three *topographic* regions, *central, middle*, and *peripheral*, were separated towards the whole-genome sequencing (WGS) study embracing a total of 18 experimental samples. The coding and non-coding sequence variation, including structural variation, was assessed and then evaluated together with the previously reported transcriptomic outcomes for the same CE samples and full-thickness corneas.

**Results:**

First, pathway enrichment analysis of genes with identified coding variants pointed to “Antigen presentation” and “Interferon alpha/beta signaling” as the most overrepresented pathways, indicating the involvement of inflammatory responses in KTCN. Both coding and non-coding sequence variants were found in genes (or in their close proximity) linked to the previously revealed KTCN-specific cellular components, namely, “Actin cytoskeleton”, “Extracellular matrix”, “Collagen-containing extracellular matrix”, “Focal adhesion”, “Hippo signaling pathway”, and “Wnt signaling” pathways. No genomic heterogeneity across the corneal surface was found comparing the assessed *topographic* regions. Thirty-five chromosomal regions enriched in both coding and non-coding KTCN-specific sequence variants were revealed, with a most representative 5q locus previously recognized as involved in KTCN.

**Conclusion:**

The identified genomic features indicate the involvement of innate and adaptive immune system responses in KTCN pathogenesis.

## 1 Introduction

Keratoconus (KTCN) is the most common corneal ectasia, characterized by progressive stromal thinning and the presence of the keratoconic cone (1). The structural changes, which often manifest asymmetrically, lead to a loss of visual acuity as an effect of irregular astigmatism (2). The first symptoms usually occur in adolescence, and the disease progresses through the third or fourth decade of life (1). Keratoconus affects both genders, but the reason of its higher occurrence in male patients remains unclear (3). The occurrence of KTCN may vary depending on the ethnicity of patients (1), with an estimated prevalence of 1.38 per 1,000 in a general population (4).

Advanced KTCN remains one of the most common indications for full-thickness (penetrating keratoplasty) and partial-thickness (deep anterior lamellar keratoplasty) corneal transplantation in developed countries (5). In the face of the organ shortage crisis in worldwide transplantation, there is a need for other therapies that inhibit the progression of KTCN to an advanced stage. A corneal collagen cross-linking (CXL) is an effective alternative used to stabilize the progression of KTCN and often improves the quality of patients’ vision (6).

Histopathological abnormalities are present in all corneal layers of the KTCN cornea (7), with diverse manifestations in central and peripheral zones being an effect of different biomechanical tension across corneal curvature in KTCN (8). Changes in collagen fibers and other elements of the extracellular matrix (ECM) form stromal irregularities (8) contributing to the KTCN cone formation and also affect the corneal epithelium (CE). The resulting rebuilt epithelial structure is characterized by a central thinning surrounded by a thickened annulus, named *doughnut* pattern (9). In the morphological observations of the CE (including confocal and immunofluorescence microscopy), the decreased cell density and their elongated shape, disruptions in basal integrity, increased number of apoptotic cells, and depositions of iron particles have been previously described (7, 10, 11). Moreover, some molecular proteomic and transcriptomic findings showed disruption of elements of Wnt signaling (e.g., *WNT10A*) (12), cell–cell communications (e.g., CDH13) (13), pro-inflammatory cytokines (e.g., *IL6*) (14), matrix metalloproteinases (e.g., MMP9) (15), and innate immune system (e.g., TLR2 and TLR4) (16).

The available reports regarding molecular and environmental findings in KTCN studies (17–22) indicate the multifactorial nature of the disease. The behavioral, environmental, and socioeconomic factors supposedly induce disease manifestation/progression in genetically predisposed individuals. In clinical studies, KTCN was found to be related to chronic eye rubbing, allergy, atopy, asthma, and UV exposure (18).

Historically, KTCN has been described as a non-inflammatory disease (2), but in the last decade, this aspect has been widely studied and questioned by ophthalmologists. However, the currently valid Global Consensus on Keratoconus and Ectatic Diseases (23) has not addressed this issue.

Previously published transcriptomic and proteomic findings derived a hint for the influence of inflammatory factors in KTCN as the increased levels of inflammatory cytokines (as *IL6, TNFα*, and *MMP9*) were found in patients’ tear fluid and serum samples and suggested to contribute to the thinning and weakening of the corneal tissue in KTCN (17, 24–26).

So far, the genetic aspects regarding the functioning of the immune system in the KTCN have not been demonstrated. Numerous genetic findings, including familial inheritance (27, 28), a concordance between monozygotic twins in contrast to dizygotic twins (29), and the occurrence of syndromic KTCN (30), imply the presence of a genetic component for the disease development. The previous results obtained by our research group confirmed the postulated genetic heterogeneity between patients and the involvement of numerous genetic factors in the pathogenesis of KTCN (19, 31–33). Besides the identification of the 13q32 (31), 5q31.1-q35.3 (32), 2q13-q14.3, and 20p13-p12.2 (33) KTCN loci, the numerous sequence variants in *VSX1, TGFBI, DOCK9, STK24, IPO5, SKP1* and *IL17B* candidate genes (19, 32) have been demonstrated. So far, whole-genome sequencing (WGS) has not been applied in KTCN research. As only WGS provides an overview of the entire human genome, and it is a suitable method for causative variant discovery in genetically heterogeneous diseases, this approach is a natural next step in investigating the pathogenesis of KTCN. We hypothesize that variants in non-coding regions of the genome complement the previously identified features specific to the KTCN exome.

The multilevel structural and functional changes in cornea, including the varied morphology and various intensities of inflammatory-causing internal biomechanical tension and external environmental stimuli (e.g., eye rubbing) acting on particular zones of CE, suggest the diverse molecular features across corneal curvature. These premises support the designation and separation of three *topographic* regions of the CE, namely, *central*, *middle*, and *peripheral*. Here, we assessed the genomic diversity across keratoconic cone surface implementing a unique study design embracing the three *topographic* regions of the CE of adolescent patients with KTCN.

## 2 Materials and methods

### 2.1 Ophthalmic examination and patients’ inclusion and exclusion criteria

Unrelated adolescent patients with KTCN and the youngest available controls (the non-KTCN individuals with mild myopia) were involved in this study. The study protocol was approved by the Bioethics Committee at Poznan University of Medical Sciences, Poznan, Poland. The possible consequences of the study were explained, and informed consent was obtained from all participants, according to the Declaration of Helsinki.

Each individual underwent a complete ophthalmological examination, including the assessments of both uncorrected (UCVA) and best-corrected visual acuity (BCVA), intraocular pressure (IOP), corneal tomography with rotating Scheimpflug camera WaveLight^®^ Oculyzer^™^ II (Alcon, TX, USA), epithelial thickness mapping [Spectral-Domain Optical Coherence Tomography (SD-OCT) device, Zeiss Cirrus 5000, Carl Zeiss Meditec, Dublin, California, USA], and slit-lamp and dilated funduscopic examination. A questionnaire comprising the behavioral, environmental, and socioeconomic aspects, including eye rubbing, use of contact lenses, atopy, UV exposure, smoking, reading habits, time spent in front of a screen, hormone intake, education level, and place of living, was completed by each participant.

The inclusion and exclusion criteria for adolescents with KTCN, and control individuals are described in detail in **Supplementary Methods 1.1**.

### 2.2 CXL and PRK procedures

CXL in patients with KTCN was performed in accordance with the standard Dresden Protocol (34), while the PRK was performed as a refractive error correction procedure (35) in control individuals as described in **Supplementary Methods 1.2, 1.3** respectively.

### 2.3 Material collection and sample preparation

Stamps towards the nose and eyebrow were made on the CE before the excision in the CXL/PRK procedures to enable correct tissue orientation during cutting and separation of the *topographic* regions. The obtained tissues were submersed in an RNA stabilization solution (RNAlater; Qiagen, Hilden, Germany) immediately after excision and stored at −80°C until further proceeding.

**Figure 1:**
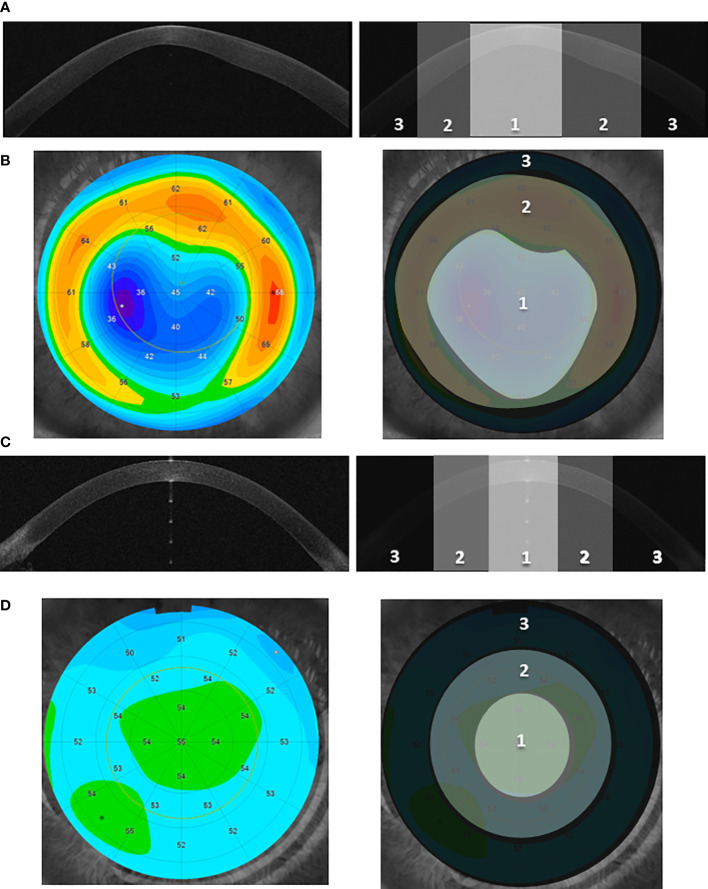
The designation of the three corneal *topographic* regions. **(A, C)** Examples of the cross-sectional image of the anterior segment of the eye (Anterior Segment Optical Coherence Tomography, AS-OCT) for the KTCN adolescent **(A)** and control individual **(C)**, respectively. Three distinct *topographic* regions (1—*central*, 2—*middle*, and 3—*peripheral*) are indicated. The KTCN-specific thinning at the 1—*central* region is visible. **(B, D) **Example of the epithelial thickness mapping for the KTCN adolescent **(B) **and control individual **(D)**, respectively. Representative images were obtained using MS-39 AS-OCT (CSO, Firenze, Italy). The *topographic* regions: 1—*central*, 2—*middle*, and 3—*peripheral*, were designated based on the steepness of epithelial thickness and corneal tomography with a rotating Scheimpflug camera (not shown). The designated CE *topographic* regions were separated and processed towards the DNA extraction.

The procedure of designation of the particular *topographic* region is shown in **Figures 1**, **S1**. The details of the sample preparation are described in **Supplementary Methods 1.4**.

### 2.4 DNA extraction

Separated CE samples were transferred from the microscope slides to the lysis solution (Norgen Biotek, Thorold, ON, Canada). Total RNA, DNA, and proteins were extracted and purified according to the instructions of the RNA/DNA/Protein Purification Plus Micro Kit (Norgen Biotek). The quantity of DNA samples was measured by Qubit dsDNA HS Assay Kit (Invitrogen, Thermo Fisher Scientific, Waltham, MA, USA) and quality was assessed by 1% gel electrophoresis.

### 2.5 Library preparation, sequencing, and WGS data analyses

WGS of the 18 CE samples was performed with a TruSeq Nano DNA HT Library Prep Kit (Illumina, San Diego, CA, USA) and the HiSeqX platform (Illumina) with mean coverage depth 30× at CloudHealth Genomics (Shanghai, China) as previously described (36). WGS data processing is summarized in the **Supplementary Methods 1.5**.

The following subset of sequence variants were analyzed: high impact variants (nonsense mutations/stop gain, stop lost, frameshifts, splice site mutations, etc.), missense variants (missense mutations considered deleterious by either SIFT or PolyPhen), and variants in regulatory regions [overlapping promoters, promoter flanking regions, enhancers, CCCTC-binding factor binding sites, transcription factor (TF) binding sites, or open chromatin regions, based on the Ensembl Regulatory Build for fibroblasts], classified as single-nucleotide variant (SNV)/deletion/insertion/indel/sequence alteration (substitution).

Motif analysis of variants located in regulatory elements (REs) was executed using the web-based FABIAN-variant application (37), and SNVs were additionally verified using MotifbreakR (38).

A set of impacted genes (high impact/missense/regulatory regions variants) was assessed for each patient after removing variants identified in control patients and variants with a MAF_AF (Maximum observed allele frequency in 1000 Genomes, ESP, and gnomAD) ≥0.1 (1000 Genomes Project phase three, ESP6500SI-V2, gnomAD v2.1). The non-redundant set of impacted genes in KTCN patients was analyzed using ConsensusPathDB (39), ShinyGO (40), STRING tool (41), and Reactome database (42). In pathway enrichment and gene ontology analyses, the p-value ≤ 0.05 and/or FDR ≤ 0.05 were the cutoffs.

The identification of genomic regions significantly enriched in genes with KTCN-specific sequence variants, compared to the density of genes in the background, was performed using ShinyGO (40). A “sliding window” is applied to scan the genome and multiple hypergeometric tests to determine whether the evaluated genes are significantly overrepresented within the analyzed window with a size of 6 Mbp and the FDR ≤ 0.05 as a cutoff.

The Integrative Genomics Viewer (IGV) (43) was used for visual exploration of genomic data.

To determine if there is any difference between the genomic characteristics of the particular CE *topographic* regions and full-thickness corneas, we compared the data obtained for the CE samples with our previously generated molecular data for the same population (Polish Caucasians) (19, 32, 44, 45) and with other published data on candidate genes and gene pathways (31, 46–51).

All experimental samples were analyzed separately and at no step of the analysis (including the analysis of raw next generation sequencing reads) were data from the three regions of one individual combined. In the Results section, the variant(s) referred as recognized in the KTCN patient were present in all three *topographic* regions.

### 2.6 Data integration

Simultaneously, the transcriptomic and proteomic assessments as well as morphological evaluation in the same CE samples were performed, as described in detail elsewhere (13). All the data of the mentioned investigation were considered in final interpretation of the study outcomes.

## 3 Results

### 3.1 Characteristics of patients and DNA samples

Four unrelated adolescent patients with KTCN (one female/three male patients) and two control individuals (two male individuals) were involved in this study. There was no history of KTCN and genetic diseases (including autoimmune diseases) in families of ascertained patients and controls. Clinical characteristics of the examined individuals and the eyes subjected to the surgery are presented in **Table 1**, while the information collected for both eyes in the studied individuals is compiled in **S1 Table**. Selected behavioral, environmental, and socioeconomic aspects evaluated in the questionnaire are presented in **S2 Table**.

**Table 1:** The clinical characteristics of the ascertained adolescent patients with KTCN and control individuals (non-KTCN mild myopia patients).

ID	Diagnosis	Study subgroup	Sex	Age at examination	Age at diagnosis	K1 [D]	K2 [D]	Kmax [D]	Anterior elevation [um]	Posterior elevation [μm]	TCT [µm]	AL [mm]	Cone/apex^X^ location	Thinnest epithelial thickness	Average thickness of central region-1 [μm]	Average thickness of middle region-2 [μm]	Average thickness of periphery region-3 [μm]	KTCN grade
Adolescent KTCN																		
10 OPT/KTCN	KTCN	Adolescent	M	14	14	49.5	54.8	66.2	+39	+52	391	24.2	Central	39	35	57	47	3
13 OPT/KTCN	KTCN	Adolescent	M	16	16	44.1	45.8	50.3	+11	+30	489	22.45	Inferior, temporal	39	42	49	45	2
18 OPT/KTCN	KTCN	Adolescent	M	14	13	42.8	44.5	49.6	+14	+28	413	23.71	Inferior, central	34	41	47	44	1–2
30 OPT/KTCN	KTCN	Adolescent	F	13	12	45.1	49.9	58.9	+31	+50	437	24.31	Central, temporal	35	39	56	47	2–3
Controls																		
5 OPT/M	Myopia	Control	M	24	nd	43.3	44.2	44.4	+2	+2	531	23.31	Central	41	52	50	49	n/a
6 OPT/M	Myopia	Control	M	30	nd	41.3	42.8	43.1	+1	+1	489	25.09	Central	46	49	46	45	n/a

M, male; F, female; K1, flat keratometric readings; K2, steep keratometric readings; Kmax, maximum simulated keratometry; OD, right eye; OS, left eye; nd, data not available; n/a, not applicable; TCT, thinnest corneal thickness; AL, Axial length; ^X^, applicable for control. Clinical data concerning only the eyes subjected to surgery are presented.

A total of 18 experimental samples of CE (three *topographic* regions in each of the six ascertained individuals) were collected and used in the WGS experiments, in accordance with a study scheme (**Figures 1**, **S1**). The results of quantity and quality control of the 18 DNA samples and quality control of WGS reads are presented in **S3 Table**.

### 3.2 Genes shaping the inflammatory responses

Analyzing the coding sequence, a total of 646 variants were identified in KTCN patients, including high impact and missense variants (classified as SNVs, deletions, and insertions). In aspects of molecular function, biological process, and their cellular location, genes with identified variants contributed to ECM structural constituent, collagen-containing ECM, and cytoskeleton organization (**S2 Figure** and **S4 Table**). What is important, the pathway enrichment analysis comprising all genes with recognized sequence variation revealed the “Antigen presentation” and “Interferon alpha/beta signaling” as the most overrepresented Reactome pathways (FDR < 0.001, **S5 Table**). In STRING analysis, embracing the same set of genes with the 646 variants, the significant enrichment of 63 clusters, including network of interferon alpha/beta signaling, was recognized (**S3 Figure** and **S6 Table**).

Seventeen out of 646 variants were found as recurring in patients with KTCN, whereas the remaining 158, 159, 187, and 165 variants identified in patients 10 OPT/KTCN, 13 OPT/KTCN, 18 OPT/KTCN, and 30 OPT/KTCN, respectively, were unique for the evaluated individuals. The recurrent coding variants were as follows: rs687485 in *KIR2DL*; rs643861 and rs652641 in *KIR3DL1*; rs878913005, rs878949904, and rs1269243287 in *MUC5AC*; rs767573621 and rs752917826 in *TRAV19*; rs17229 in *TRBV12-5*; rs2257251 in *NANOGP8*; rs1227351091 in *NBPF19;* rs148235978 in *NFE2L3;* rs151080920 in *TSC22D2*; rs140771568 in *UCMA;* rs28575804 (A>G) in *UGT2B25P;* rs1563130264 in *AC011005.1*; and rs879192097 in *AL354822.1* (**S7 Table**).

**Figure 2:**
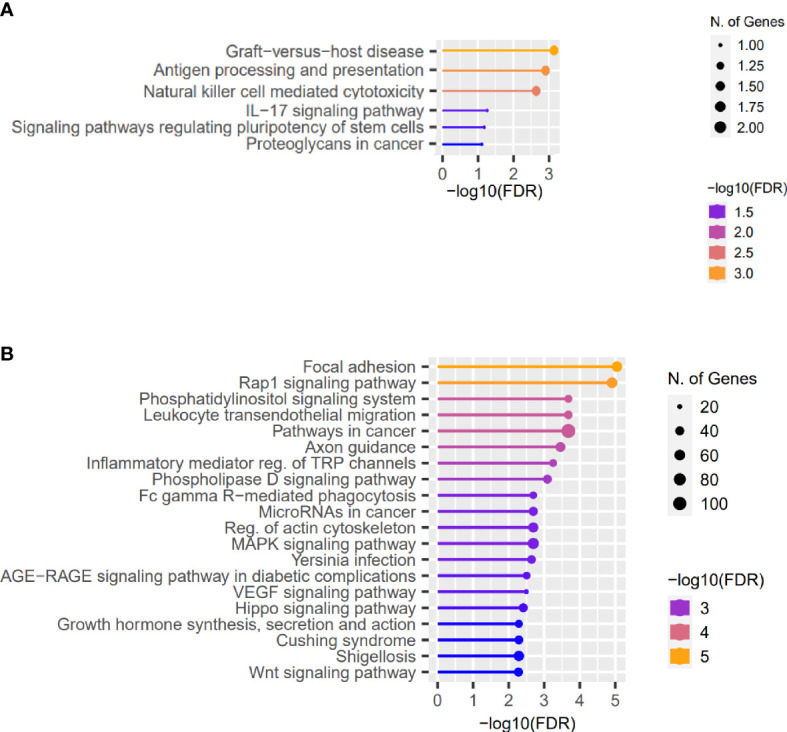
Representative results of the pathway enrichment analysis. Representative results of the pathway enrichment analysis of **(A)** genes with the same coding sequence variants identified in more than one KTCN patient, pointing to the involvement of innate and adaptive immune system responses in KTCN pathogenesis and **(B)** genes located close to the variants in REs. On *x* axes, −log(FDR) values of enriched pathways are presented, and on *y* axes, the KEGG pathways are presented. The size of the bubbles indicates the number of genes attributed to the particular pathway and present in our set.

Furthermore, analyzing the high-impact and missense variants identified in at least two patients with KTCN, the “Graft-versus-host disease” and “Antigen processing and presentation” were found to be the most enriched pathways (**Figure 2A** and **S8 Table**).

### 3.3 KCTN-specific non-coding sequence features

Excluding variants recognized in control individuals and variants with MAF>0.1, 15,213 sequence variants in REs in KTCN patients, classified as SNVs, deletions, insertions, indels, and substitutions, were identified.

Next, using the FABIAN-variant application that predicts the effects of DNA variant located in RE on transcription factor binding sites (TFBSs), we narrowed down the number of variants and genes for further evaluation to 7,886 and 5,709, respectively. We assessed genes in which close proximity in at least one sequence variant in RE per KTCN patient was recognized as potentially forming or abolishing TFBS. Pathway enrichment analysis of these genes disclosed “Focal adhesion”, “Rap1 signaling pathway”, and also “Hippo signaling pathway”, “Wnt signaling”, and “TGF-beta signaling” pathways (**Figure 2B** and **S9 Table**).

### 3.4 Coexistence of coding and non-coding sequence variants in genes influencing the KTCN-specific pathways

Both coding and non-coding sequence variants of KTCN patients were found in the genes/genes’ close proximity linked to the revealed KTCN-specific cellular components, namely, “Actin cytoskeleton”, “Extracellular matrix”, and “Collagen-containing extracellular matrix”, and KTCN-specific pathways such as “Focal adhesion”, “Hippo signaling pathway”, and “Wnt signaling” (**S4 Figure** and **S10 Table**). Genes with coding and non-coding sequence variants, attributed to biological pathways from Reactome database, are listed in **Table 2**, and in detail in **S11 Table**.

**Table 2:** Chosen WGS results, list of genes with high impact, missense, and variants in regulatory elements attributed to the selected biological pathways.

Pathway module, Pathway	Genes with variants
Cytokine signaling, Interferon α/β	***HLA-A**, **MX2**, **XAF1**, IRF1^‡^, IFITM3^‡^, IRF2, IFI35, ISG20, IFI27, TYK2, OAS1*
Cytokine signaling, Interferon γ	***HLA-A**, **ICAM1**, **GBP7**, IRF1^‡^, IFITM3^‡^, GBP5, GBP1, MT2A, PTPN2, PML, OAS1, IRF2, TRIM31, SUMO1, CD44, NCAM1, IFNGR2*
Cytokine signaling, Interleukin-1	***PTPN14**, **SQSTM1**, **PSMD3**, IL1R1^‡^, PTPN12^‡^, PELI2, TAB3, PTPN20, TAB1, IL1RAP, PTPN2, APP, SIGIRR, IL1RL1, UBE2N, PSMD1, UBE2V1*
Cytokine signaling, Interleukin-6	***CNTFR**, OSM, TYK2, IL6ST, OSMR, STAT3*
Innate immune system, Neutrophil degranulation	***PECAM1^†‡^**, **ATPSCKMT**, **HPSE**, **A1BG**, **HLA-A**, **TOM1**, **GALNS**, **PFKL**, **CEP290**, **GUSB**, **PIEZO1**, **EPO**, **PSMD3**, **LILRA6**, **ITGAD**, **EPX**, **IQGAP2**, **ATAD3A**, ANO6^‡^, TMC6^‡^, GDI2^‡^, HSPA6^‡^, S100Z^‡^, FCGR3B^‡^, RAB7B^‡^, XRCC5, KCMF1, FCGR3B, GOLGA7, ATP8B4, PRC1, RAB18, IQGAP1, CPNE1, RNF38, RAP1B, PTPRC, RAP1A, PLD1, ADA2, CTSC, SH3GLB2, CEACAM6, RHOG, TMC6, IST1, CEACAM1, CEACAM3, PSMB1, RAB31, RHOA, PKP1, CYFIP1, CFD, STK10, CD44, DDX3X, DOCK2, RAB7B, SLC27A2, ITGAV, LTF, KCNAB2, GDI2, PSMA5, RAB44*
Adaptive Immune System, Class I MHC antigen processing and presentation	***HLA-A**, **UBE2Q2**, **TOM1**,**FBXO27**, **PSMD3**, **RNF213**, **KBTBD13**, **SH3RF1**, **CDRT1**, **ANAPC10**, NEDD4L^‡^, SMURF1^‡^, S100Z^‡^, ZNRF1^‡^, SPSB1^‡^, ASB2^‡^, PJA2^‡^, ANAPC5, TRIM9, RNF126, TRIM41, FBXL7, ANAPC1, MIB2, PSMD1, FGG, FBXL20, UBE3B, UBA6, UBE3D, UBE3C, RNF19A, CDC27, HERC4, TRIM32, LNX1, TRIM36, UBE2N, PSMC6, PRKN, FBXO32, FBXO15, SMURF2, HECTD2, FBXO17, UBE2H, UBE2K, CDRT1, RNF220, KLHL3, PSMB1, LMO7, RNF217, PXK, ITGB5, ITGAV, UBE2V2, UBE2V1, FBXW9, FBXW7, ANAPC13, PSMA5, PATJ*
Signal transduction, Signaling by TGFB family members	***TGIF1**, **CCNT1**, NEDD4L^‡^, SERPINE1^‡^, SMURF1^‡^, FST^‡^, CDKN2B, EMB, SMOX, SMURF2, WWTR1, FKBP1B, STAG1, E2F5, ITGA8, TGFB3, FKBP1A, USP15, LTBP1, RHOA, TFAM, HDAC1, NCOR2, ITGB5, CCNT2, ITGAV, FBN1, SMAD4, SMAD1, CDK6, PRKCZ, SMAD6, CDK9*
Signal transduction, Signaling by WNT	***LGR5**, **ROR2**, **PRKCB**, **PSMD3**, **HECW1**, **CCDC88C**, NFATC1^‡^, SMURF1^‡^, PPP2R1A^‡^, LRP5^‡^, RAC2, CSNK1E, EMB, WNT3A, H2AC6, PRKCB, FZD1, VANGL2, WLS, PSMD1, HECW1, YWHAZ, TCF7L2, GNB5, ITPR1, ITPR2, LGR4, CUL3, TCF4, LGR5, APC, TNRC6C, KRAS, PRKG1, TNRC6B, H3-3B, GSK3B, H3-3A, GNG2, PPP3CA, BCL9, CREBBP, PSMC6, AXIN1, H3C2, AXIN2, SMURF2, H2BC6, PDE6A, WNT5B, DACT1, PSMB1, TLE4, RHOA, CTBP1, CTBP2, ROR1, LRTM1, HDAC1, PLCB1, PPP2R5C, RSPO2, NLK, DAAM1, PSMA5*
Extracellular matrix organization, Integrin cell surface interaction	***PECAM1^†‡^**, **TNC**, **FN1**, **COL23A1**, **VWF**, **COL5A1**, **ITGAD**, **ICAM1**, ITGA4^‡^, COL5A2^‡^, COL8A1, COL6A3, ITGAD, COL13A1, ITGA11, COL5A1, CD44, ITGB5, VTN, ITGB7, ITGAV, FBN1, JAM3, ITGA8, TNC, ITGA2B, HSPG2, FGG*
Extracellular matrix organization, Collagen formation	***LAMC2**, **P3H1**, **COL23A1**, **COL5A1**, **COL17A1**, **COLGALT1**, COL27A1^‡^, ADAMTS2^‡^, P4HA1^‡^, COL5A2^‡^, COL21A1, COL8A1, COL6A3, COL25A1, COLGALT2, COL5A1, LOXL4, DST, PXDN, COL17A1, LOXL2, LAMC2, PLOD2, COL28A1, COL14A1, LOX, COL13A1, COL12A1*
Extracellular matrix organization, ECM proteoglycans	***TNC**, **FN1**, **LAMB1**, **COL5A1**, **LAMA5**, **ITGAD**, TNR^‡^, COL5A2^‡^, SERPINE1^‡^, COL6A3, MUSK, COL5A1, LRP10, ITGA8, TGFB3, ITGA2B, ACAN, DCN, LAMC1, LAMA1, ASPN, LAMA2, APP, LAMA5, PTPRS, ITGB5, ITGAV, MATN1, TNC, NCAM1, VTN, ITGAD, TNXB, HSPG2*
Extracellular matrix organization, Degradation of ECM	***FN1**, **LAMB1**, **LAMC2**, **COL23A1**, **COL5A1**, **COL17A1**, **LAMA5**, **CAPN9**, **CAPN12**, **NID1**, COL5A2^‡^, COL8A1, COL6A3, COL25A1, COL5A1, GPR37, CAST, MEGF11, MME, SUB1, ACAN, COL17A1, DCN, HTRA1, LAMC1, LAMC2, COL14A1, COL13A1, TLL2, COL12A1, MMP17, CD44, FBN2, LAMA5, ADAMTS9, ADAM17, FBN1, PLG, ELN, CAPN13, NID1, CAPN15, HSPG2*
Developmental biology, Keratinization	***EVPL^†^**, **PCSK6^†^**, **KRTAP10-7**, **KRT35**, **FLG**, **KRT33B**, PKP3, KRTAP5-5, PKP4, PKP1, PI3, KRTAP4-11, KRTAP3-1, SPRR1B, KAZN, KRT39*
Cell–cell communication, Cell–cell junction organization	***LAMC2^†‡^**, **COL17A1^†‡^**, **SDK1^†‡^**, **NECTIN2**, **SDK2**, CLDN6^‡^, FLNC, CLDN8, LIMS1, LIMS2, PARVA, CLDN2, PARVB, MEGF11, DST, VASP, PARD6B, ACTB, CDH6, LAMC2, CLDN16, FERMT2, CTNNA1, NECTIN3, CLDN20, ACTN1, PATJ*
Chromatin modifying enzymes, Methylation	***WDR5B**, **KDM2D**, **SMYD3**, PRDM16^‡^, KDM7A^‡^, H3C2, KDM4C, KDM4B, KDM2B, SMYD3, EHMT1, WDR5, SETD7, DOT1L, SETD3, EZH2, CCND1, H2AC6, SMARCA2, ARID1B*

Bold text indicates genes with coding sequence variants identified in more than one KTCN patient; ^†^ stands for genes with variants in both and non-coding genome sequence; ^‡^ stands for genes with the same variant identified in more than one KTCN patient. Variants identified in control samples and variants with MAF≥0.1 in the gnomAD database were excluded (the completed list of genes with sequence variants is presented in **S9 Table**).

**Figure 3:**
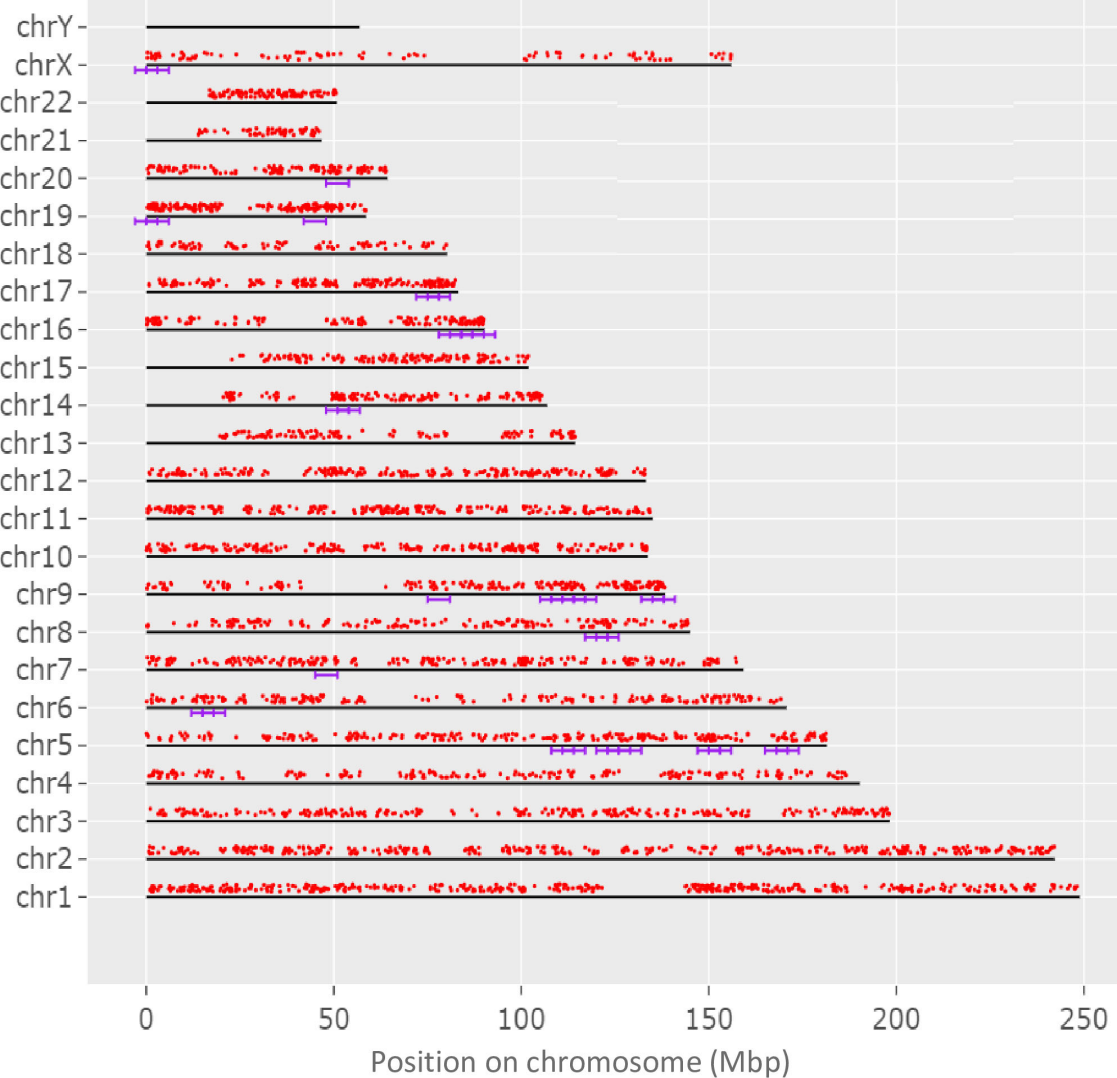
The display of genes, in which both coding and non-coding variants unique for adolescent patients with KTCN were identified, across chromosomes. Genes are represented by red dots. The violet lines indicate 35 regions statistically enriched. The genome was scanned with a sliding window in of size 6 Mbp. Within each window, the hypergeometric tests were used to determine if genes of both coding and non-coding variants were significantly overrepresented. The used FDR cutoff was ≤0.05.

To determine if the evaluated genome regions are significantly enriched, genes with both coding and non-coding sequence variants were analyzed and 35 enriched regions were revealed, as presented in **Figure 3**, with majority of these enriched regions at chromosomes 5q (13 regions), 9q (9 regions), and 16q (5 regions).

### 3.5 Genomic heterogeneity across corneal surface and structural variation

All identified coding-sequence variants were assessed for differences between *topographic* regions, separately for each adolescent patient with KTCN. No genomic heterogeneity across corneal surface was found. Although identified sequence variants in *KIR3DL1* (in two KTCN patients), *UMAD1* (in two KTCN patients), and *GOLGA6A2* (in one KTCN patient) genes have met quality criteria of reads in the applied bioinformatic algorithms and differentiated the *topographic* CE regions, the BAM files visualized with the IGV showed a variation in one of 26–43 reads only in these listed genes (the total number of reads for a particular genome site varied), indicating uncertain results. Expanding the same analysis for control samples (but applying the criterion of MAF ≤ 0.1), the additional coding sequence variants in 15 genes (*AC018682.2, AC092384.1, AC092490.1, AC117481.1, AC118281.1, ANKRD36BP2, CA15P2, CRACD, HLA-DRB5, KRTAP2-2, LINC00634, NBEAP1, OR2T35, SAMD1*, *UGT2B25P*, and*ZNF571*) were recognized as potentially differentiating the corneal surface, but none of these variants was confirmed by the BAM files.

Assessing structural variation, deletions longer than 50 bp were characterized in detail. The length of the deletions and their density in genome are presented in **S5 Figure**. Most of the deletions were localized in non-coding regions of the genome and in gene introns. We did not observe a difference in the number of deletions comparing patients with KTCN and control individuals, assessing the previously published KTCN-specific loci (**S12 Table**) (52). Nevertheless, upstream and downstream regions of genes involved in the innate immune system (*ITGAM, PRKCE, COL11A2, STK10, ADGRG3, IGHV2-70, IGHG4, MAP2K1, IGHG2, GSDMD, MLC1, MYO9B, IGHV1-69, DEFB131A, KIR2DS4, NKIRAS2, RNF38*, and *RPS6KA5*); the adaptive immune system (*TRAC, INPP5D, IGHV2-70, ASB3, PRKG1, TAP2, HMCN2, KIR3DL2, KIR3DL1, IGHV1-69, KIR2DL4, RASGRP3*), cytokine signaling (*ITGAM, HNRNPA2B1, NUP155, CAMK2A, INPP5D, IGHG4, MAP2K1, CRLF2, GSDMD, NKIRAS2, BOLA2*, and *RPS6KA5*); ECM including collagen biosynthesis, formation, and trimerization (*ITGA9, FBLN1, LOXL4, COL6A6, ITGAM, ADAMTS14, P4HA3, COL11A2, COL27A1*, and *COL26A1*); and others were found to contain deletions (51–5,098 bp in size) in patients with KTCN in contrast to control individuals (MAF > 0.1 was an exclusion criterion).

### 3.6 Influence of the presence of RE variants on gene expression

**Figure 4:**
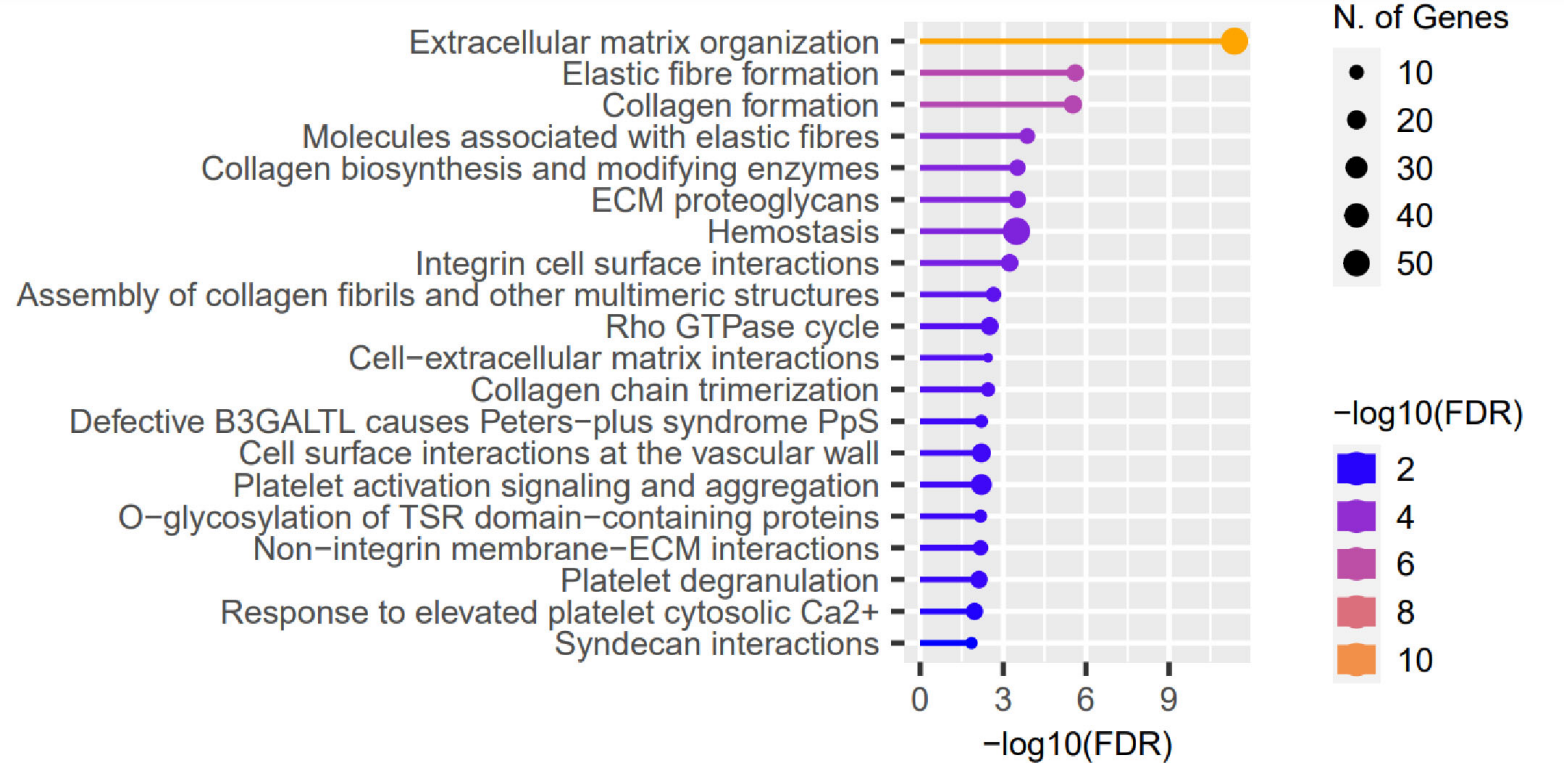
Representative results of the pathway enrichment analysis of 722 genes previously recognized as differentially expressed (DEGs) in full-thickness KTCN corneas and re-recognized as containing variants in the REs localized in close proximity. On the *x* axis, −log(FDR) values of enriched pathways are presented, and on the *y* axis, the KEGG pathways are presented. The size of bubbles indicated the number of genes attributed to the particular pathway and present in our set. The Reactome database was chosen for the definition of pathways.

We evaluated the influence of the presence of identified RE variants on expression of genes localized in close proximity, based on data of RNA-seq performed in the same CE samples, and full-thickness corneas (45). No substantial features were recognized in the assessment of the CE. However, 722 of the previously recognized differentially expressed genes (DEGs) in full-thickness KTCN corneas were re-recognized as the gene containing variants in REs. Then, in the pathway enrichment analysis of the re-recognized genes and transcripts, the ECM pathway as the most overrepresented was revealed (**Figure 4** and **S13 Table**).

## 4 Discussion

The complex background of KTCN, in addition to environmental factors, includes heterogeneous genetic characteristics. The multiple candidate genes and variants identified using different molecular techniques indicate that this heterogeneity requires the introduction of high-throughput investigation methods, which may be more effective in unraveling the KTCN-specific determinants.

Each individual genome differs from the reference human genome by an average of 3.5 million SNPs, but only a minority of those variations contributes to the phenotypic differences and disease predisposition (53). Only WGS can provide an overview of the entire human genome, allowing the global analysis and the full set of variants to be assessed (54–56). The majority of SNVs and indels is identifiable by both WGS and exome sequencing (ES), but still approximately 3% of coding variants is missed in the ES approach (54). Therefore, WGS is more efficient than ES for detecting coding sequence variants, besides the undoubted benefit of identification of non-coding sequence variation. To date, WGS has not been applied in KTCN research. Since WGS is an effective method of detecting mutations in genetically heterogeneous diseases, including ophthalmic diseases (57), and is more efficient than ES in the identification of sequence variation, this technique is crucial in deciphering the genetic aspects of KTCN.

Here, we found that the genetic features of patients with KTCN correspond with those previously reported regarding sequence variants in components of ECM (19, 50). Also, we identified other sequence variants in genes previously reported in KTCN research in the same population (19), including *COL17A1* [playing a role in the integrity of hemidesmosome and the attachment of basal keratinocytes (58), whose expression was decreased in corneal epithelial cells in single-cell RNA-seq analysis of KTCN corneas (59)]; *FREM2* [protein required for maintenance of the integrity of the skin epithelium (60) and eye morphogenesis (60)]; *SSX2IP* [which may connect the nectin–afadin and E-cadherin–catenin system through alpha-actinin and may be involved in the organization of the actin cytoskeleton (61)]; *MINK1* [mediating stimulation of the stress-activated protein kinase MAPK14/p38 MAPK downstream of the Raf/ERK pathway (62)]; *CAMSAP1* [Calmodulin-regulated spectrin-associated protein 1; key microtubule-organizing protein (63)]; and *RGS12* [Regulator of G-protein signaling 12 (64)]. Moreover, we identified variants previously reported in *EML6* (50), which is an element of cytoskeletal network and microtubules in cultured keratocytes (50, 65); *MAGI1* (50), which regulates cell–cell and cell–matrix adhesion (66); and *COL5A1* (48, 49), whose knockout in corneal stroma in mouse showed severe dysfunctional regulation of fibrillogenesis resulting in decreased fibril number and a disorganized lamellae structure with decreased stroma thickness (67).

Also, we have revealed variants in genes involved in keratin-related pathways (namely, the “keratinization” pathway from the Reactome database), such as *EVPL, PCSK6, KRTAP10-7, KRT35, FLG*, and *KRT33B.* As keratins participate in forming intermediate filaments (IF), which provide mechanical support, and form desmosomes between cells and hemi-desmosomes with basement membranes for epithelium integrity (68), this result may be of special interest regarding changes in biomechanics of keratoconic cornea.

Importantly, non-coding variants are now considered to be potentially pathogenic (69, 70). Sequence variation in the form of SNVs or indels in the regulatory regions can either generate or abolish TF binding sites, modulating the transcriptional activity of genes and regulating chromatin state (71). The disruption of the TF binding site and its effect on the disease phenotype have already been reported in many cancer types such as T‐cell acute lymphoblastic leukemia (72) and esophageal and gastric cancers (73). However, while great advances in predicting the effects of coding variants have been made, the assessment of non-coding variants remains challenging. In particular, the immense number of variants revealed per patient sample impedes the analysis and biological interpretation. In our investigation, all variants located in REs, including promoters, promoter flanking regions, enhancers, CCCTC-binding factor binding sites, TFBS, or open chromatin regions of fibroblasts, were evaluated *in silico* towards functional effect (TFBS gain/lost) based on the motif analysis. The obtained list of RE variants showed unity on the pathway level with previously reported variants, namely, focal adhesion (19), Wnt signaling (19), and TGF-beta signaling (74). Moreover, the *LAMC2, COL17A1, SDK1, PECAM1, TJP2, MINK1, NEIL3, EVPL*, and *PCSK6* genes were revealed to have unique variants for patients with KTCN in both coding and non-coding genome sequence, which again were found as involved in the ECM, cell–cell communications, cytoskeleton organization, and keratinization pathways. To further evaluate the effect of variants located in REs, the functional approaches such as gene reporter assay are needed, but firstly transcriptome data from adequate biological material is necessary to be integrated with the genomic data. Therefore, we evaluated the data overlap between DEGs in full-thickness KTCN corneas (45) and variants recognized in the REs. We found the involvement of RE variants in numerous elements of ECM and that fact supports our assumption concerning the role of RE sequence variation in KTCN pathogenesis.

Although single variants could influence the KTCN phenotype in some families (75), our genomic data (both coding and non-coding sequence variation) suggest that KTCN largely occurs through the interplay of multiple variants that interfere with major physiological processes in the cornea. The presented connection between coding and non-coding sequence variants in the gene ontology (“Actin cytoskeleton”, “Extracellular matrix”, and “Collagen-containing extracellular matrix”) and in the pathway assessments (“Focal adhesion”, “Hippo signaling pathway”, and “Wnt signaling”) corroborates this assumption, as well as the identified 13 regions enriched in variants in the 5q locus that had previously been found to be specific to KTCN in distinct populations (32, 46, 47).

It has been known that DNA structural variation modifies the interactions between REs and their target genes (71). Here, the majority of the deletions were found to be located in non-coding and intron regions. Overall, assessing the previously published KTCN-specific loci, we did not observe a difference in the number/density of deletions comparing patients with KTCN and control individuals. However, numerous genes involved in the innate immune system; adaptive immune system; ECM including collagen biosynthesis, formation, and trimerization; and others were found to contain deletions in size of 51–5,098 bp in patients with KTCN in contrast to control individuals. The extent to which this finding is important should be clarified in further research on this aspect. To date, deletions >50 bp in size themselves and their effect on the KTCN phenotype were only once evaluated, in an Ecuadorian family with KTCN (31), but that study did not reveal any copy number variation on evaluated 13q32 in the tested individuals.

We have anticipated that the various intensities of both internal biomechanical tension and external environmental stimuli (e.g., eye rubbing) acting on various *topographic* regions of CE could result in diverse molecular features across corneal curvature. Our study performed in the designated three *topographic* regions of the CE, *central, middle*, and *peripheral*, enabled the conclusion that there is no genomic heterogeneity across the corneal surface in KTCN. However, in a comprehensive morphological, transcriptomic, and proteomic evaluation performed in the same corneal samples, we identified the variability shaping the distinct features of CE zones (13). Continuing the topographic aspects of the CE, we have considered features of mosaicism to be involved in the process of remodeling of the KTCN CE. To date, based on the results of exome and/or Sanger sequencing performed in matched KTCN cornea–blood pairs, we found no evidence of inter-tissue variants in human KTCN corneas, since all variants possibly related to KTCN were identified in both corneal tissue and peripheral blood of patients (19). Again, here we did not identify features of mosaicism.

In our opinion, the most important findings of this study are those concerning the inflammation in KTCN. Nowadays, the inflammatory aspects in KTCN are widely discussed. The multiple analyses of the tear cytokine and protease profiles of patients with KTCN have shown a persistent inflammation (17, 76). Previously, we identified numerous, randomly distributed variants in genes that encoded inflammatory molecules (*IL1A* and *IL1B*) and one variant (c.214+242C>T in the *IL1RN*) presented in all KTCN individuals (33). Here, the enrichment in variants of genes involved in the innate (neutrophil degranulation) and adaptive (Class I MHC antigen processing and presentation) immune system was revealed. In our study, *TRAV19* and *TRVB12-5* genes were recognized with three recurring variants (one as a stop gain). Because these genes encode variable regions of T-cell receptors, it could indirectly influence the process of antigen processing (77). Importantly, in a single-cell study of full corneas, the upregulation of the T-cell receptor signaling pathway has been recognized (59). Identified recurring missense variants in *KIR2DL1* and *KIR3DL1* could influence the activity of the immune system, since they are members of the highly conserved killer cell immunoglobulin-like receptors (KIRs) family (78), which are transmembrane glycoproteins expressed by natural killer (NK) cells and subsets of T cells (79). It was previously reported that the variant in *KIR3DL1* has affected the NK cell function (80). NK cells activate neutrophils and induce both their infiltration to the inflamed sites and degranulation (81). Also, NK cells were reported to participate in acute immune response in dry eye syndrome and trigger Th-17 response, manifested by an increase of IL17 (82). Moreover, the involvement of NK cells in the corneal wound healing has been confirmed in a murine model (83). The evidence for migration of cells into corneal limbus and central stroma after a  2-mm epithelial abrasion of the mouse cornea and altered inflammatory reaction after antibody-induced depletion of NK cells were presented (83). Previously, as elements of innate immune responses, the expression of TLR2 and TLR4 has been studied by flow cytometry in corneal epithelial cells and blood samples of KTCN patients and their relatives (16, 84, 85), and significant upregulation has been shown, indicating a potential of the TLR2 and TLR4 proteins to be the KTCN biomarkers. However, no sequence variants in the TLRs were recognized here as well as in our current gene and protein expression assessment (13). Instead, we recognized sequence variants of other innate immune system elements (e.g., *MUC5AC*, as mucins consist the first-line defense). Moreover, the increased proportions of activated NK cells as well as neutrophils and γδT cells have been reported in KTCN studies (86). Also in many studies of tear fluid collected in KTCN patients, the higher levels of interleukins (IL1β, IL6, and IL17A), interferons (IFNα/β/γ), and other pro-inflammatory factors (TNFα, TGFβ1, LIF, granzyme-B, perforin, and MMP2) were recognized (14, 24, 86–88). Besides published transcriptomic and proteomic findings, the genomic aspects regarding disruption of immune responses have not been demonstrated in KTCN research. Also, the here-identified single high-impact sequence variants (*CEP290, MMRN1*, and *FN1*) and missense variants (*PECAM1, VWF, CD109, HRG, TLN1, HPSE, EPO, SIGLEC10, DOCK8, ERBB3*, and *AP3B1*) could contribute to molecular dysregulation of wound healing, followed by inflammation, although we have not experimentally determined their impact.

Therefore, we conclude here that the disturbance of the immune responses in KTCN affects various elements of corneal hemostasis, including the antigen presentation and neutrophil degranulation processes, and possibly results in thinning and weakening of the KTCN corneas.

Summarizing, applied WGS allowed for identification and confirmation of numerous genomic features in KTCN. In future studies, WGS data should be integrated with other genome-wide analyses data, including chromatin accessibility (Assay for Transposase Accessible Chromatin with sequencing, ATAC-Seq), transcriptomics, and proteomic data, in order to obtain the most reliable research results. In this study, owing to a limited number of included patients, we intently did not focus on particular sequence variations but rather on the sets of impacted genes and the pathways in which those genes are involved. Additionally, we decided to evaluate adolescent patients with KTCN only, assuming the more pronounced genetic background of the disease. The recruitment of the control group was especially challenging, as nowadays the photorefractive keratectomy (PRK) procedure, during which the CE is removed, is rarely performed and is replaced with the newer techniques of vision correction without CE removal. The youngest possible patients with minimal vision impairment undergoing the PRK procedure were ascertained into the control group to minimize potential bias. The PRK procedure is performed in adults, and this fact also results in the difference in age between the compared groups of patients. Although only four patients and two controls were involved, 18 samples were tested in the WGS experiments and data obtained from those 18 samples were carefully curated. In addition, the sequence variant interpretation was performed using additional allele frequency data derived from the reference databases (1000 Genomes, ESP and gnomAD). Owing to the small size of the compared study groups, we were unable to subdivide these groups into subgroups of patients, taking into account the presence of an allergic disease or dry eye syndrome.

The multiple coding and non-coding sequence variants were found in genes (or in their close proximity) contributing to the previously discussed KTCN-specific cellular components, and newly presented aspects of innate and adaptive immune system responses, pointing to the involvement of inflammatory aspects in KTCN. Therefore, we conclude that variants in non-coding regions of the genome have complemented the previously identified features specific to the KTCN exome.

Additionally, the 35 chromosomal regions shown enriched in both coding and non-coding KTCN-specific sequence variants confirmed the heterogenous background of KTCN with a limited number of common elements.

We did not find the diversity in genetic features of the assessed *topographic* regions of CE. This lack of genomic heterogeneity across the corneal surface and no evidence of inter-tissue variants in human KTCN corneas suggest other molecular mechanisms of KTCN-specific changes in CE.

## Data availability statement

The original contributions presented in the study are publicly available. This data can be found here: https://www.ncbi.nlm.nih.gov/clinvar/ under the accession number SUB13057607.

## Ethics statement

The studies involving human participants were reviewed and approved by Bioethics Committee at Poznan University of Medical Sciences, Poznan, Poland. Written informed consent to participate in this study was provided by the participants’ legal guardian/next of kin.

## Author contributions

KJ: participated in research design, sample collection, and patient surveys; performed sample preparations towards WGS experiments; participated in bioinformatics analyses; figures and tables preparation; participated in the genomic and clinical data interpretation; and wrote the manuscript. MM-K: performed recruitment of the patients and clinical evaluation; facilitated sample collection; performed the clinical data interpretation; participated in the genomic data interpretation; and manuscript writing. MK: performed setup of bioinformatics pipeline; bioinformatics analyses of the genomic data; and preparation of figures and tables. JAK: participated in the genomic data interpretation. MG: research design; secured research funding; participated in the genomic data interpretation; and edited the manuscript. All authors contributed to the article and approved the submitted version.

## References

[1] Ferrari G Rama P. The keratoconus enigma: a review with emphasis on pathogenesis. Ocular Surface (2020) 18:363–73. doi: 10.1016/j.jtos.2020.03.006 32234342

[2] Rabinowitz YS. Keratoconus. Surv Ophthalmol (1998) 42:297–319. doi: 10.1016/s0039-6257(97)00119-7 9493273

[3] Fink BA Wagner H Steger-May K Rosenstiel C Roediger T McMahon TT. Differences in keratoconus as a function of gender. Am J Ophthalmol (2005) 140:459–68. doi: 10.1016/j.ajo.2005.03.078 16083843

[4] Hashemi H Heydarian S Hooshmand E Saatchi M Yekta A Aghamirsalim M. The prevalence and risk factors for keratoconus: a systematic review and meta-analysis. Cornea (2020) 39:263–70. doi: 10.1097/ICO.0000000000002150 31498247

[5] Gain P Jullienne R He Z Aldossary M Acquart S Cognasse F. Global survey of corneal transplantation and eye banking. JAMA Ophthalmol (2016) 134:167–73. doi: 10.1001/jamaophthalmol.2015.4776 26633035

[6] O’Brart DPS Kwong TQ Patel P McDonald RJ O’Brart NA. Long-term follow-up of riboflavin/ultraviolet a (370 nm) corneal collagen cross-linking to halt the progression of keratoconus. Br J Ophthalmol (2013) 97:433–7. doi: 10.1136/bjophthalmol-2012-302556 23385632

[7] Naderan M Jahanrad A Balali S. Histopathologic findings of keratoconus corneas underwent penetrating keratoplasty according to topographic measurements and keratoconus severity. Int J Ophthalmol (2017) 10:1640–6. doi: 10.18240/ijo.2017.11.02 PMC5686360 29181305

[8] Scarcelli G Besner S Pineda R Kalout P Yun SH. *In vivo* biomechanical mapping of normal and keratoconus corneas. JAMA Ophthalmol (2015) 133:480–2. doi: 10.1001/jamaophthalmol.2014.5641 PMC4698984 25611213

[9] Silverman RH Urs R RoyChoudhury A Archer TJ Gobbe M Reinstein DZ. Epithelial remodeling as basis for machine-based identification of keratoconus. Invest Ophthalmol Vis Sci (2014) 55:1580. doi: 10.1167/iovs.13-12578 24557351 PMC3954156

[10] Khaled ML Helwa I Drewry M Seremwe M Estes A Liu Y. Molecular and histopathological changes associated with keratoconus. BioMed Res Int (2017) 2017:7803029. doi: 10.1155/2017/7803029 28251158 PMC5303843

[11] Sherwin T Brookes NH. Morphological changes in keratoconus: pathology or pathogenesis. Clin Exp Ophthalmol (2004) 32:211–7. doi: 10.1111/j.1442-9071.2004.00805.x 15068441

[12] Foster JW Parikh RN Wang J Bower KS Matthaei M Chakravarti S. Transcriptomic and immunohistochemical analysis of progressive keratoconus reveal altered WNT10A in epithelium and bowman’s layer. Invest Ophthalmol Vis Sci (2021) 62:16. doi: 10.1167/iovs.62.6.16 PMC8132000 33988693

[13] Jaskiewicz K Maleszka-Kurpiel M Matuszewska E Kabza M Rydzanicz M Malinowski R. The impaired wound healing process is a major factor in remodeling of the corneal epithelium in adult and adolescent patients with keratoconus. Invest Ophthalmol Vis Sci (2023) 64:22. doi: 10.1167/iovs.64.2.22 PMC9970004 36811882

[14] Shetty R Sathyanarayanamoorthy A Ramachandra RA Arora V Ghosh A Srivatsa PR. Attenuation of lysyl oxidase and collagen gene expression in keratoconus patient corneal epithelium corresponds to disease severity. Mol Vis (2015) 21:12–25. 25593510 PMC4301596

[15] Yam GH-F Fuest M Zhou L Liu Y-C Deng L Chan AS-Y. Differential epithelial and stromal protein profiles in cone and non-cone regions of keratoconus corneas. Sci Rep (2019) 9:2965. doi: 10.1038/s41598-019-39182-6 30814630 PMC6393548

[16] Malfeito M Regueiro U Pérez-Mato M Campos F Sobrino T Lema I. Innate immunity biomarkers for early detection of keratoconus. Ocular Immunol Inflammation (2019) 27:942–8. doi: 10.1080/09273948.2018.1511813 30230940

[17] Balasubramanian SA Pye DC Willcox MDP. Effects of eye rubbing on the levels of protease, protease activity and cytokines in tears: relevance in keratoconus. Clin Exp Optom (2013) 96:214–8. doi: 10.1111/cxo.12038 23496656

[18] Gordon-Shaag A Millodot M Shneor E Liu Y. The genetic and environmental factors for keratoconus. BioMed Res Int (2015) 2015:795738. doi: 10.1155/2015/795738 26075261 PMC4449900

[19] Karolak JA Gambin T Rydzanicz M Polakowski P Ploski R Szaflik JP. Accumulation of sequence variants in genes of wnt signaling and focal adhesion pathways in human corneas further explains their involvement in keratoconus. PeerJ (2020) 8:e8982. doi: 10.7717/peerj.8982 32328353 PMC7164425

[20] Nowak-Malczewska DM Karolak JA Swierkowska J Jaworska MM Kulinska KI Polakowski P. Changes in nuclear gene expression related to mitochondrial function affect extracellular matrix, collagens, and focal adhesion in keratoconus. Transl Vis Sci Technol (2021) 10:6. doi: 10.1167/tvst.10.11.6 PMC8419871 34478492

[21] Özalp O Atalay E Yıldırım N. Prevalence and risk factors for keratoconus in a university-based population in Turkey. J Cataract Refract Surg (2021) 47:1524–9. doi: 10.1097/j.jcrs.0000000000000669 33929805

[22] Sahebjada S Chan E Xie J Snibson GR Daniell M Baird PN. Risk factors and association with severity of keratoconus: the Australian study of keratoconus. Int Ophthalmol (2021) 41:891–9. doi: 10.1007/s10792-020-01644-6 33200389

[23] Gomes JAP Tan D Rapuano CJ Belin MW Ambrósio R Guell JL. Group of panelists for the global Delphi panel of keratoconus and ectatic diseases. Global consensus keratoconus ectatic diseases. Cornea (2015) 34:359–69. doi: 10.1097/ICO.0000000000000408 25738235

[24] Lema I Sobrino T Durán JA Brea D Díez-Feijoo E. Subclinical keratoconus and inflammatory molecules from tears. Br J Ophthalmol (2009) 93:820–4. doi: 10.1136/bjo.2008.144253 19304583

[25] Lema I Durán JA. Inflammatory molecules in the tears of patients with keratoconus. Ophthalmology (2005) 112:654–9. doi: 10.1016/j.ophtha.2004.11.050 15808258

[26] López-López M Regueiro U Bravo SB Chantada-Vázquez MDP Varela-Fernández R Ávila-Gómez P. Tear proteomics in keratoconus: a quantitative SWATH-MS analysis. Invest Ophthalmol Vis Sci (2021) 62:30. doi: 10.1167/iovs.62.10.30 PMC8399462 34431975

[27] Hutchings H Ginisty H Le Gallo M Levy D Stoësser F Rouland JF. Identification of a new locus for isolated familial keratoconus at 2p24. J Med Genet (2005) 42:88–94. doi: 10.1136/jmg.2004.022103 15635082 PMC1735904

[28] Brancati F. A locus for autosomal dominant keratoconus maps to human chromosome 3p14-q13. J Med Genet (2004) 41:188–92. doi: 10.1136/jmg.2003.012872 PMC1766922 14985379

[29] Bechara SJ Waring GO Insler MS. Keratoconus in two pairs of identical twins. Cornea (1996) 15:90–3. 8907387

[30] Elder MJ. Leber congenital amaurosis and its association with keratoconus and keratoglobus. J Pediatr Ophthalmol Strabismus (1994) 31:38–40. doi: 10.3928/0191-3913-19940101-08 8195961

[31] Gajecka M Radhakrishna U Winters D Nath SK Rydzanicz M Ratnamala U. Localization of a gene for keratoconus to a 5.6-Mb interval on 13q32. Invest Ophthalmol Vis Sci (2009) 50:1531–9. doi: 10.1167/iovs.08-2173 PMC4547351 19011015

[32] Karolak JA Gambin T Pitarque JA Molinari A Jhangiani S Stankiewicz P. Variants in SKP1, PROB1, and IL17B genes at keratoconus 5q31.1-q35.3 susceptibility locus identified by whole-exome sequencing. Eur J Hum Genet (2017) 25:73–8. doi: 10.1038/ejhg.2016.130 PMC5159765 27703147

[33] Nowak DM Karolak JA Kubiak J Gut M Pitarque JA Molinari A. Substitution at *IL1RN* and deletion at *SLC4A11* segregating with phenotype in familial keratoconus. Invest Ophthalmol Vis Sci (2013) 54:2207. doi: 10.1167/iovs.13-11592 23462747

[34] Wollensak G Spoerl E Seiler T. Riboflavin/ultraviolet-a-induced collagen crosslinking for the treatment of keratoconus. Am J Ophthalmol (2003) 135:620–7. doi: 10.1016/s0002-9394(02)02220-1 12719068

[35] Ganesh S Brar S Patel U. Comparison of ReLEx SMILE and PRK in terms of visual and refractive outcomes for the correction of low myopia. Int Ophthalmol (2018) 38:1147–54. doi: 10.1007/s10792-017-0575-6 28551832

[36] Karolak JA Vincent M Deutsch G Gambin T Cogné B Pichon O. Complex compound inheritance of lethal lung developmental disorders due to disruption of the TBX-FGF pathway. Am J Hum Genet (2019) 104:213–28. doi: 10.1016/j.ajhg.2018.12.010 PMC6369446 30639323

[37] Steinhaus R Robinson PN Seelow D. FABIAN-variant: predicting the effects of DNA variants on transcription factor binding. Nucleic Acids Res (2022) 50:W322–9. doi: 10.1093/nar/gkac393 PMC9252790 35639768

[38] Coetzee SG Coetzee GA Hazelett DJ. *motifbreakR* : an R/Bioconductor package for predicting variant effects at transcription factor binding sites: fig. 1. Bioinformatics (2015), 31(23):3847–9. doi: 10.1093/bioinformatics/btv470 PMC4653394 26272984

[39] Herwig R Hardt C Lienhard M Kamburov A. Analyzing and interpreting genome data at the network level with ConsensusPathDB. Nat Protoc (2016) 11:1889–907. doi: 10.1038/nprot.2016.117 27606777

[40] Ge SX Jung D Yao R. ShinyGO: a graphical gene-set enrichment tool for animals and plants. Bioinformatics (2020) 36:2628–9. doi: 10.1093/bioinformatics/btz931 PMC7178415 31882993

[41] Szklarczyk D Gable AL Nastou KC Lyon D Kirsch R Pyysalo S. The STRING database in 2021: customizable protein-protein networks, and functional characterization of user-uploaded gene/measurement sets. Nucleic Acids Res (2021) 49:D605–12. doi: 10.1093/nar/gkaa1074 PMC7779004 33237311

[42] Gillespie M Jassal B Stephan R Milacic M Rothfels K Senff-Ribeiro A. The reactome pathway knowledgebase. Nucleic Acids Res (2022) 50:D687–92. doi: 10.1093/nar/gkab1028 PMC8689983 34788843

[43] Robinson JT Thorvaldsdóttir H Winckler W Guttman M Lander ES Getz G. Integrative genomics viewer. Nat Biotechnol (2011) 29:24–6. doi: 10.1038/nbt.1754 PMC3346182 21221095

[44] Kabza M Karolak JA Rydzanicz M Udziela M Gasperowicz P Ploski R. Multiple differentially methylated regions specific to keratoconus explain known keratoconus linkage loci. Invest Ophthalmol Vis Sci (2019) 60:1501. doi: 10.1167/iovs.18-25916 30994860

[45] Kabza M Karolak JA Rydzanicz M Szcześniak MW Nowak DM Ginter-Matuszewska B. Collagen synthesis disruption and downregulation of core elements of TGF-β, hippo, and wnt pathways in keratoconus corneas. Eur J Hum Genet (2017) 25:582–90. doi: 10.1038/ejhg.2017.4 PMC5437911 28145428

[46] Bisceglia L De Bonis P Pizzicoli C Fischetti L Laborante A Di Perna M. Linkage analysis in keratoconus: replication of locus 5q21.2 and identification of other suggestive loci. Invest Ophthalmol Vis Sci (2009) 50:1081–6. doi: 10.1167/iovs.08-2382 18978346

[47] Bykhovskaya Y Li X Taylor KD Haritunians T Rotter JI Rabinowitz YS. Linkage analysis of high-density SNPs confirms keratoconus locus at 5q chromosomal region. Ophthalmic Genet (2016) 37(1):109–10. doi: 10.3109/13816810.2014.889172 PMC4139481 24555746

[48] Li X Bykhovskaya Y Haritunians T Siscovick D Aldave A Szczotka-Flynn L. A genome-wide association study identifies a potential novel gene locus for keratoconus, one of the commonest causes for corneal transplantation in developed countries. Hum Mol Genet (2012) 21:421–9. doi: 10.1093/hmg/ddr460 PMC3276283 21979947

[49] Lu Y Vitart V Burdon KP Khor CC Bykhovskaya Y Mirshahi A. Genome-wide association analyses identify multiple loci associated with central corneal thickness and keratoconus. Nat Genet (2013) 45:155–63. doi: 10.1038/ng.2506 PMC3720123 23291589

[50] Shinde V Sobreira N Wohler ES Maiti G Hu N Silvestri G. Pathogenic alleles in microtubule, secretory granule and extracellular matrix-related genes in familial keratoconus. Hum Mol Genet (2021) 30:658–71. doi: 10.1093/hmg/ddab075 PMC8867208 33729517

[51] Xu X Zhang X Cui Y Yang H Ping X Wu J. Three novel variants identified within ECM-related genes in Chinese han keratoconus patients. Sci Rep (2020) 10:5844. doi: 10.1038/s41598-020-62572-0 32246022 PMC7125089

[52] Karolak JA Gajecka M. Genomic strategies to understand causes of keratoconus. Mol Genet Genomics (2017) 292:251–69. doi: 10.1007/s00438-016-1283-z PMC5357269 28032277

[53] Gonzaga-Jauregui C Lupski JR Gibbs RA. Human genome sequencing in health and disease. Annu Rev Med (2012) 63:35–61. doi: 10.1146/annurev-med-051010-162644 22248320 PMC3656720

[54] Belkadi A Bolze A Itan Y Cobat A Vincent QB Antipenko A. Whole-genome sequencing is more powerful than whole-exome sequencing for detecting exome variants. Proc Natl Acad Sci U.S.A. (2015) 112:5473–8. doi: 10.1073/pnas.1418631112 PMC4418901 25827230

[55] Esser D Holze N Haag J Schreiber S Krüger S Warneke V. Interpreting whole genome and exome sequencing data of individual gastric cancer samples. BMC Genomics (2017) 18:517. doi: 10.1186/s12864-017-3895-z 28683819 PMC5501078

[56] Sun Y Ruivenkamp CAL Hoffer MJV Vrijenhoek T Kriek M van Asperen CJ. Next-generation diagnostics: gene panel, exome, or whole genome? Hum Mutat (2015) 36:648–55. doi: 10.1002/humu.22783 25772376

[57] González-Del Pozo M Fernández-Suárez E Bravo-Gil N Méndez-Vidal C Martín-Sánchez M Rodríguez-de la Rúa E. A comprehensive WGS-based pipeline for the identification of new candidate genes in inherited retinal dystrophies. NPJ Genom Med (2022) 7:17. doi: 10.1038/s41525-022-00286-0 35246562 PMC8897414

[58] Löffek S Hurskainen T Jackow J Sigloch FC Schilling O Tasanen K. Transmembrane collagen XVII modulates integrin dependent keratinocyte migration via PI3K/Rac1 signaling. PloS One (2014) 9:e87263. doi: 10.1371/journal.pone.0087263 24505282 PMC3914815

[59] Collin J Queen R Zerti D Bojic S Dorgau B Moyse N. A single cell atlas of human cornea that defines its development, limbal progenitor cells and their interactions with the immune cells. Ocular Surface (2021) 21:279–98. doi: 10.1016/j.jtos.2021.03.010 PMC8343164 33865984

[60] Zhang X Wang D Dongye M Zhu Y Chen C Wang R. Loss-of-function mutations in FREM2 disrupt eye morphogenesis. Exp Eye Res (2019) 181:302–12. doi: 10.1016/j.exer.2019.02.013 30802441

[61] Reis AH Xiang B Ossipova O Itoh K Sokol SY. Identification of the centrosomal maturation factor SSX2IP as a wtip-binding partner by targeted proximity biotinylation. PloS One (2021) 16:e0259068. doi: 10.1371/journal.pone.0259068 34710136 PMC8553094

[62] Nicke B Bastien J Khanna SJ Warne PH Cowling V Cook SJ. Involvement of MINK, a Ste20 family kinase, in ras oncogene-induced growth arrest in human ovarian surface epithelial cells. Mol Cell (2005) 20:673–85. doi: 10.1016/j.molcel.2005.10.038 16337592

[63] Zhou Z Xu H Li Y Yang M Zhang R Shiraishi A. CAMSAP1 breaks the homeostatic microtubule network to instruct neuronal polarity. Proc Natl Acad Sci U.S.A. (2020) 117:22193–203. doi: 10.1073/pnas.1913177117 PMC7486724 32839317

[64] Schroer AB Mohamed JS Willard MD Setola V Oestreich E Siderovski DP. A role for regulator of G protein signaling-12 (RGS12) in the balance between myoblast proliferation and differentiation. PloS One (2019) 14:e0216167. doi: 10.1371/journal.pone.0216167 31408461 PMC6691989

[65] Yin H Hou X Zhang T Shi L. Su y-q. participation of EML6 in the regulation of oocyte meiotic progression in mice. J BioMed Res (2019) 34:44–53. doi: 10.7555/JBR.33.20190014 35081682 PMC7007726

[66] Wörthmüller J Rüegg C. MAGI1, a scaffold protein with tumor suppressive and vascular functions. Cells (2021) 10:1494. doi: 10.3390/cells10061494 34198584 PMC8231924

[67] Sun M Chen S Adams SM Florer JB Liu H Kao WW-Y. Collagen V is a dominant regulator of collagen fibrillogenesis: dysfunctional regulation of structure and function in a corneal-stroma-specific Col5a1-null mouse model. J Cell Sci (2011) 124:4096–105. doi: 10.1242/jcs.091363 PMC3244988 22159420

[68] Kao WW-Y. Keratin expression by corneal and limbal stem cells during development. Exp Eye Res (2020) 200:108206. doi: 10.1016/j.exer.2020.108206 32882212

[69] Johnston JJ Williamson KA Chou CM Sapp JC Ansari M Chapman HM. NAA10 polyadenylation signal variants cause syndromic microphthalmia. J Med Genet (2019) 56:444–52. doi: 10.1136/jmedgenet-2018-105836 PMC7032957 30842225

[70] Wright CF Quaife NM Ramos-Hernández L Danecek P Ferla MP Samocha KE. Non-coding region variants upstream of MEF2C cause severe developmental disorder through three distinct loss-of-function mechanisms. Am J Hum Genet (2021) 108:1083–94. doi: 10.1016/j.ajhg.2021.04.025 PMC8206381 34022131

[71] Diederichs S Bartsch L Berkmann JC Fröse K Heitmann J Hoppe C. The dark matter of the cancer genome: aberrations in regulatory elements, untranslated regions, splice sites, non-coding RNA and synonymous mutations. EMBO Mol Med (2016) 8:442–57. doi: 10.15252/emmm.201506055 PMC5126213 26992833

[72] Mansour MR Abraham BJ Anders L Berezovskaya A Gutierrez A Durbin AD. Oncogene regulation. an oncogenic super-enhancer formed through somatic mutation of a noncoding intergenic element. Science (2014) 346:1373–7. doi: 10.1126/science.1259037 PMC4720521 25394790

[73] Malik MA Zargar SA Mittal B. A six-nucleotide deletion polymorphism in the casp8 promoter is associated with reduced risk of esophageal and gastric cancers in Kashmir valley. Indian J Hum Genet (2011) 17:152–6. doi: 10.4103/0971-6866.92090 PMC3276982 22345985

[74] Lin Q Zheng L Shen Z. A novel variant in TGFBI causes keratoconus in a two-generation Chinese family. Ophthalmic Genet (2022) 43:159–63. doi: 10.1080/13816810.2021.2015788 34895010

[75] Bisceglia L Ciaschetti M De Bonis P Campo PAP Pizzicoli C Scala C. VSX1 mutational analysis in a series of Italian patients affected by keratoconus: detection of a novel mutation. Invest Ophthalmol Vis Sci (2005) 46:39–45. doi: 10.1167/iovs.04-0533 15623752

[76] Acera A Vecino E Rodríguez-Agirretxe I Aloria K Arizmendi JM Morales C. Changes in tear protein profile in keratoconus disease. Eye (Lond) (2011) 25:1225–33. doi: 10.1038/eye.2011.105 PMC3178250 21701529

[77] Parkhurst M Robbins P Rosenberg S. Isolation of T cell receptors specifically reactive with mutated tumor associated antigens. J immunotherapy Cancer (2014) 2:P33:2051–1426-2-S3-P33. doi: 10.1186/2051-1426-2-S3-P33 PMC6453117 27827318

[78] Sambrook JG Bashirova A Andersen H Piatak M Vernikos GS Coggill P. Identification of the ancestral killer immunoglobulin-like receptor gene in primates. BMC Genomics (2006) 7:209. doi: 10.1186/1471-2164-7-209 16911775 PMC1559706

[79] Campbell KS Purdy AK. Structure/function of human killer cell immunoglobulin-like receptors: lessons from polymorphisms, evolution, crystal structures and mutations. Immunology (2011) 132:315–25. doi: 10.1111/j.1365-2567.2010.03398.x PMC3044898 21214544

[80] Carr WH Pando MJ Parham P. KIR3DL1 polymorphisms that affect NK cell inhibition by HLA-Bw4 ligand. J Immunol (2005) 175:5222–9. doi: 10.4049/jimmunol.175.8.5222 16210627

[81] Jensen KN Omarsdottir SY Reinhardsdottir MS Hardardottir I Freysdottir J. Docosahexaenoic acid modulates NK cell effects on neutrophils and their crosstalk. Front Immunol (2020) 11:570380. doi: 10.3389/fimmu.2020.570380 33123143 PMC7573488

[82] Zhang X Volpe EA Gandhi NB Schaumburg CS Siemasko KF Pangelinan SB. NK cells promote Th-17 mediated corneal barrier disruption in dry eye. PloS One (2012) 7:e36822. doi: 10.1371/journal.pone.0036822 22590618 PMC3348128

[83] Liu Q Smith CW Zhang W Burns AR Li Z. NK cells modulate the inflammatory response to corneal epithelial abrasion and thereby support wound healing. Am J Pathol (2012) 181:452–62. doi: 10.1016/j.ajpath.2012.04.010 PMC3409433 22728064

[84] Regueiro U López-López M Hervella P Sobrino T Lema I. Corneal and conjunctival alteration of innate immune expression in first-degree relatives of keratoconus patients. Graefes Arch Clin Exp Ophthalmol (2021) 259:459–67. doi: 10.1007/s00417-020-04929-9 32949300

[85] Sobrino T Regueiro U Malfeito M Vieites-Prado A Pérez-Mato M Campos F. Higher expression of toll-like receptors 2 and 4 in blood cells of keratoconus patiens. Sci Rep (2017) 7:12975. doi: 10.1038/s41598-017-13525-7 29021606 PMC5636878

[86] D’Souza S Nair AP Sahu GR Vaidya T Shetty R Khamar P. Keratoconus patients exhibit a distinct ocular surface immune cell and inflammatory profile. Sci Rep (2021) 11:20891. doi: 10.1038/s41598-021-99805-9 34686755 PMC8536707

[87] Chaerkady R Shao H Scott S-G Pandey A Jun AS Chakravarti S. The keratoconus corneal proteome: loss of epithelial integrity and stromal degeneration. J Proteomics (2013) 87:122–31. doi: 10.1016/j.jprot.2013.05.023 PMC3721369 23727491

[88] Jun AS Cope L Speck C Feng X Lee S Meng H. Subnormal cytokine profile in the tear fluid of keratoconus patients. PloS One (2011) 6:e16437. doi: 10.1371/journal.pone.0016437 21298010 PMC3029330

