# Supplementary material for: Sequence variants contributing to dysregulated inflammatory responses across keratoconic cone surface in adolescent patients with keratoconus
Source: Front Immunol. 2023 Jul 6;14:1197054. doi: 10.3389/fimmu.2023.1197054 (PMC10359427; doi:10.3389/fimmu.2023.1197054)
Supplement: Supplementary file 1 [file DataSheet_1.docx]

Supplementary Materials

Sequence Variants Contributing to Dysregulated Inflammatory Responses Across Keratoconic Cone Surface in Adolescent Patients with Keratoconus

**Katarzyna Jaskiewicz, Magdalena Maleszka-Kurpiel, Michał Kabza, Justyna A. Karolak, Marzena Gajecka***

*** Correspondence:** Marzena Gajecka, [gamar@man.poznan.pl](mailto:gamar@man.poznan.pl)

1. **SUPPLEMENTARY METHODS**
   1. **Ophthalmic examination and Patients’ inclusion and exclusion criteria**

The inclusion criteria for adolescents with KTCN (patients aged below 18 years, males and females) comprised the clinical features such as steepening of the anterior corneal surface, steepening of the posterior corneal surface, thinning and/or an increase in the rate of corneal thickness change from the periphery to the thinnest point, as well as KTCN observed with a perceived risk of progression in accordance with the recommendation of the Global Consensus of Keratoconus and Ectatic Diseases that children and adolescents should undergo surgery after the KTCN diagnosis without waiting for progression, due to the high risk of disease progression (1–3).

Corneal scarring and posthydrops were the main exclusion criteria in the KTCN patients. Also, corneal thickness lower than 400 µm, measured during the irradiation step of the CXL procedure was the exclusion criterion, taking into consideration the safety (avoiding potential radiation damage) of the corneal endothelium and deeper structures (4).

The control individuals’ inclusion criteria (non-KTCN patients, males, and females) comprised the mild myopia phenotype with no clinical signs of corneal ectasia/KTCN in both eyes, examined in both the corneal tomography map and the corneal epithelium thickness map, without other corneal and eye abnormalities.

Patients were not wearing contact lenses prior to examination for 4 weeks, 2 weeks, and 1 week respectively for gas permeable rigid, toric, and soft lenses.

The presence of genetic disease was the exclusion criterion in the examined study subgroups.

To avoid information bias all recruited patients and control individuals and their collected biological materials were recruited/proceeded by the same researchers and with the use of the same chemical reagents.

- 1. **CXL procedure**

CXL was performed in accordance with the standard Dresden Protocol (5–7). Briefly, the CE was removed using a blunt spatula, after 20 s exposure to 20% ethyl alcohol, under local anesthesia. Then, 0.1% riboflavin solution (Riboflavin, Ricrolin, Sooft Italia, Montegiorgio, Italy) was administered repetitively, every two minutes, within a 30-minute period, topically. The corneal stroma was irradiated using a UV-A device (PXL Platinum 330, Peschke Trade, Switzerland), using the irradiance level of 3 mW/cm^2^ for 30 minutes. Before irradiation, pachymetry was measured again by Ultrasound contact Pachymeter (Peschke Trade, Switzerland), to confirm that the thinnest part of the stroma was not below 400 µm (8,9). After irradiation, topical antibiotics, steroids, and a bandage lens were applied.

- 1. **Photorefractive keratectomy procedure**

CE samples from control individuals were collected after the photorefractive keratectomy (PRK), performed as a refractive error correction procedure (10). Briefly, after local anesthesia, exposure of corneal epithelium to 20% ethyl alcohol for the 30s was performed and epithelium was removed using a blunt spatula. Next, the excimer laser ablation was carried out using the MEL 90 excimer laser (Carl Zeiss Meditec AG, Jena, Germany). Finally, the cornea was protected with antibiotics and a bandage lens.

- 1. **Material collection and sample preparation**

Stamps towards the nose and eyebrow were made on the CE before the excision to enable correct tissue orientation during cutting and separation of the *topographic* regions.

The obtained tissues were submersed in an RNA stabilization solution (RNAlater; Qiagen, Hilden, Germany) immediately after excision and stored at −80°C until nucleic acids and proteins isolation. Directly before the isolation, the CE samples were placed on microscope slides (VWR® Superfrost® Plus Micro Slide, VWR, USA) in the correct orientation/position with the control of a light microscope (10x magnification, Zeiss Axio Scope.A1, Carl Zeiss MicroImaging GmbH, Göttingen, Germany), then fixed by immediate freezing with use of dry ice and Cryospray (Engelbrecht GmbH), and cut on dry ice using disposable razor blades. Before the CE cutting, the epithelium thickness values measured automatically using the OCT for the particular regions of CE were evaluated. Then, the specific *topographic* regions were assessed manually together with the determination of cone/apex (for KTCN and controls, respectively) location (central, superior/inferior, nasal/temporal), at the same time by the operating surgeon and the researcher processing the material.

- 1. **WGS data analyses**

Whole genome sequencing of CE samples for the selected four youngest KTCN patients and two youngest control individuals was performed with a TruSeq Nano DNA HT Library Prep Kit (Illumina, San Diego, CA, USA) and the HiSeqX platform (Illumina) with mean coverage depth 30X at CloudHealth Genomics (Shanghai, China) as previously described (11). Briefly, the short reads were trimmed using the BBDuk2 program from the BBTools suite (http://jgi.doe.gov/data-and-tools/bbtools/) to remove Illumina adapters and poor-quality regions (mean Phred quality, <5). Then, reads were mapped to reference genome GRCh38 (source: Ensembl release 100) using BWA-MEM (12). Duplicated reads were marked using Sambamba (13). SNP and indel calling has been performed using Platypus (14), and detected variants were annotated using Ensembl Variant Effect Predictor software (15). The structural variant calling has been performed using Manta (16), which uses split read and discordant paired read alignments as evidence, and cn.MOPS (17), which uses genome coverage as evidence, and Parliament2 (18), which exploit different methods/tools (e.g. Breakdancer, Breakseq2, CNVnator, Delly, Lumpy) and combine their outputs into a single consensus. Structural variants were only kept for further analysis if they had at least 90% mutual overlap with any region detected by at least one other tool. The variants interpretation was performed using additional data from population databases as the sample size and power weren't calculated.

The prediction of the effects of DNA variants located in regulatory elements (RE) on transcription factor binding sites (TFBSs) was executed using web-based application FABIAN-variant (19). Analysis was conducted including all 5014 models and all 1387 transcription factors, and variants with combined scores ≤-0.9 or ≥0.9 were further assessed as potentially forming or abolishing TFBS (TFBS loss or TFBS gain), adequately.

## SUPPLEMENTARY FIGURES AND TABLES


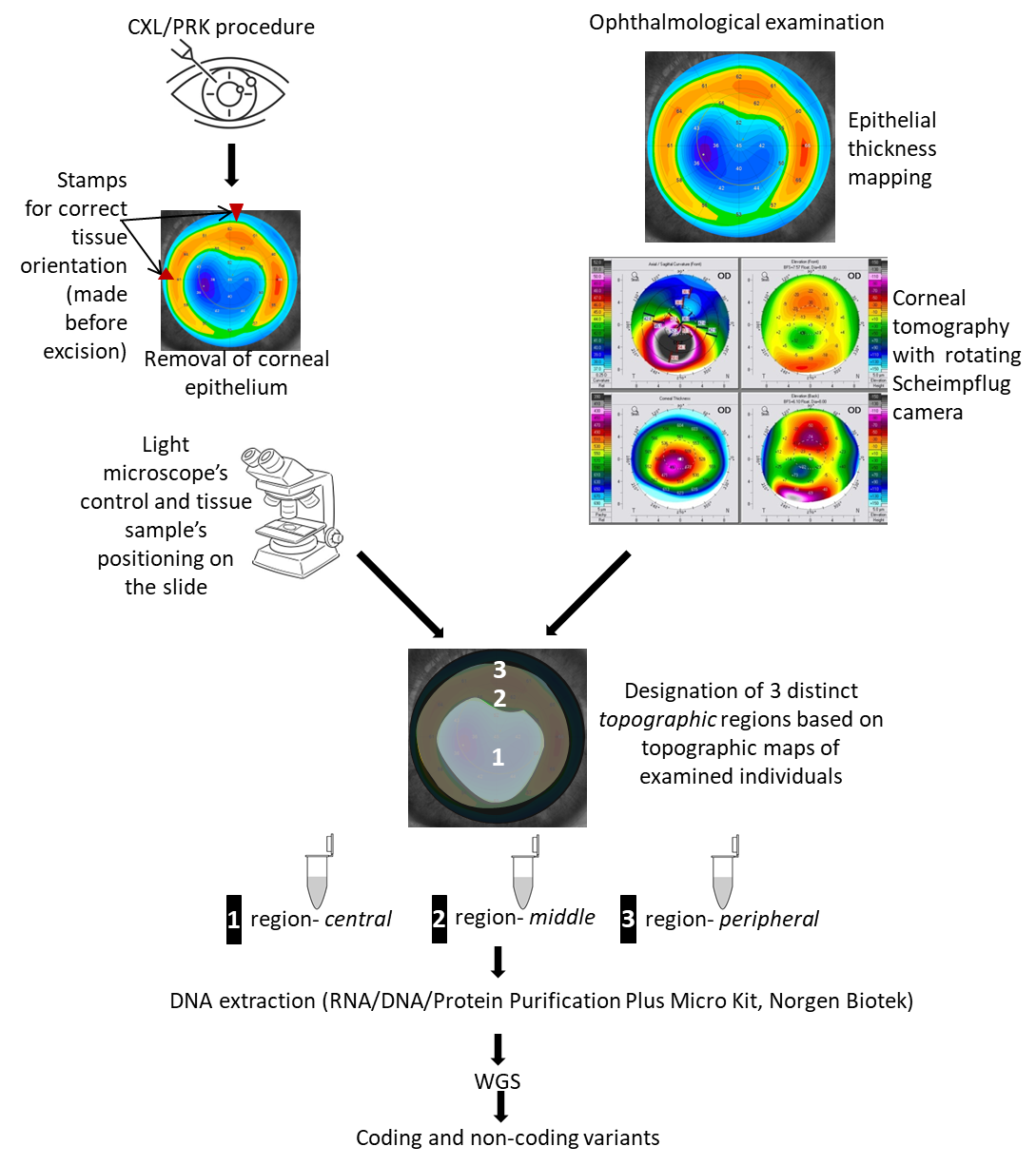


**S1 Figure. The study workflow.** The genomic features of corneal epithelium (CE) were evaluated based on the analysis of 3 distinct CE *topographic* regions (1-*central*, 2-*middle*, 3-*peripheral*). First, individuals underwent the ophthalmic examination and qualification to the CXL or PRK procedure. During the CXL/PRK procedure, the stamps for the correct tissue orientation (made before corneal epithelium removal) were done. Next, based on the epithelial thickness map and corneal tomography, 3 distinct epithelial *topographic* regions were separated. After DNA, RNA, and protein extraction, the DNA samples of the four youngest KTCN patients and two myopic controls were preceded to WGS sequencing. The variants concerning coding and non-coding genome sequence were integrated and biologically interpreted to provide an insight into the genetic background of KTCN.


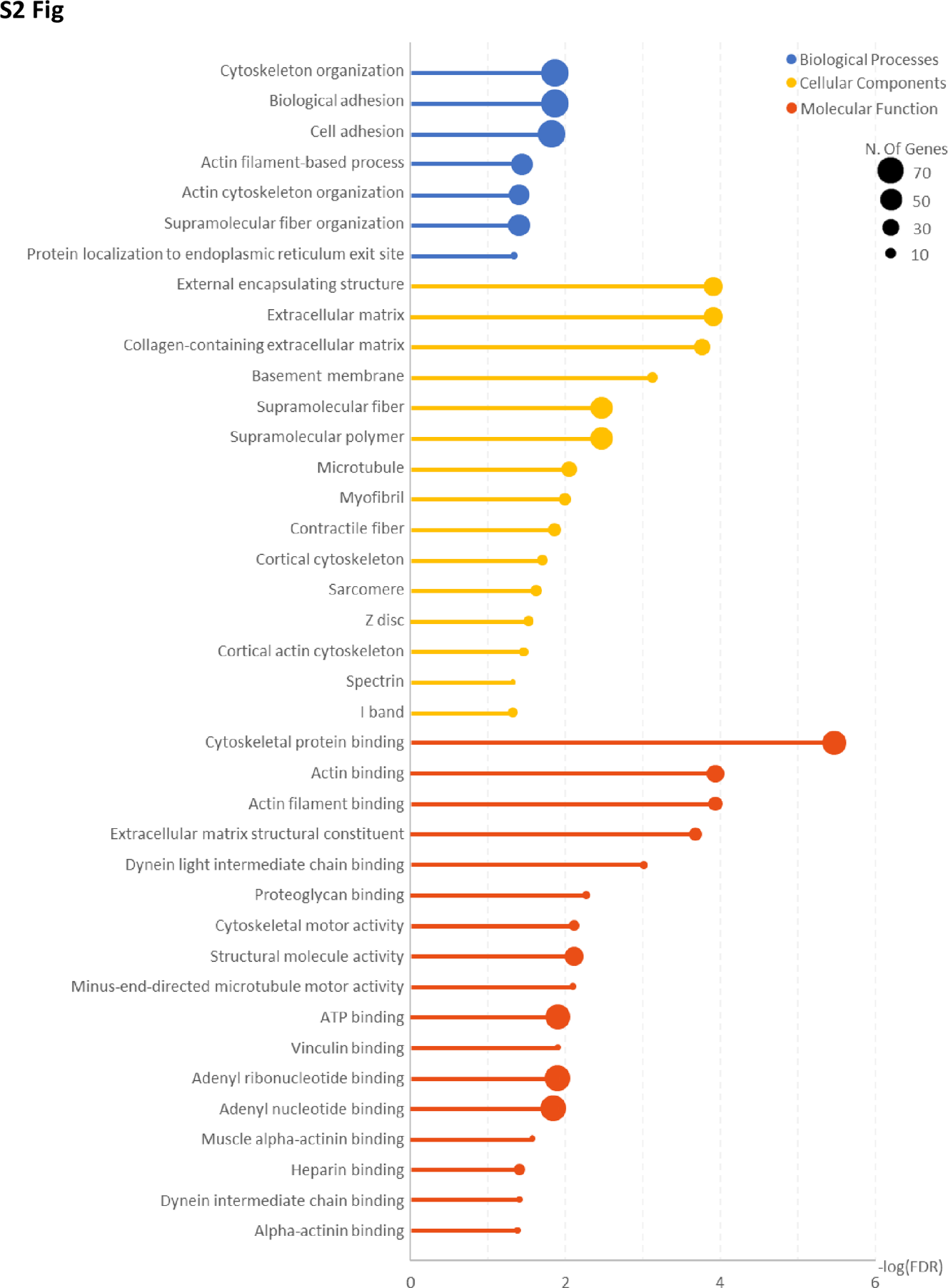


**S2 Figure.** **Representative results of Gene Ontology (GO) analysis** **of genes with variants identified in coding sequence, unique for adolescent patients with KTCN**. Presented GO terms indicate biological processes (blue color of bubbles), cellular components ( yellow color of bubbles), and molecular functions (red color of bubbles) of gene products significantly enriched (FDR≤0.05) in patients with KTCN. On the x axis -log(FDR) values of GO term enrichment, and on the y axis the GO terms are presented. The size of bubbles indicates the number of genes with identified variants attributed to particular GO term.

**
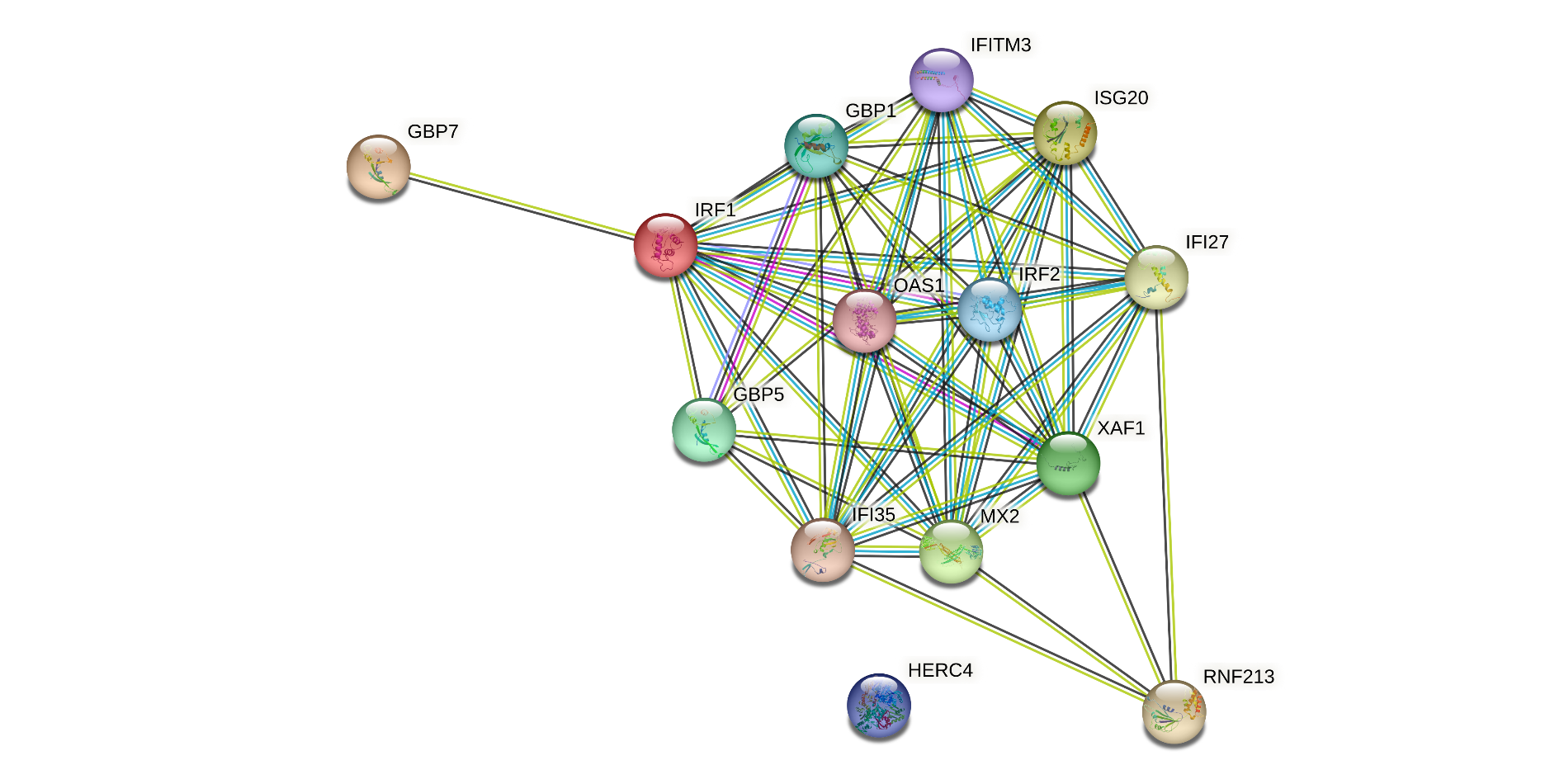
S3 Figure.** STRING protein-protein interaction map of 14 genes with variants identified in coding sequence, unique for adolescent patients with KTCN, forming association network of interferon alpha/beta signaling (cluster #19380: *Mixed, incl. Interferon alpha/beta signaling, and negative regulation of type I interferon production*; FDR=1.07e-07). Network nodes represent genes. Lines of different colors represent eight types of evidence used in predicting associations. Purple line: experimental evidence; light blue line: database evidence; red line: predicted fusion; dark green line: predicted neighborhood; dark blue line: predicted co-occurrence; light green line: textmining evidence; black line: co-expression evidence; violet line: homology evidence.


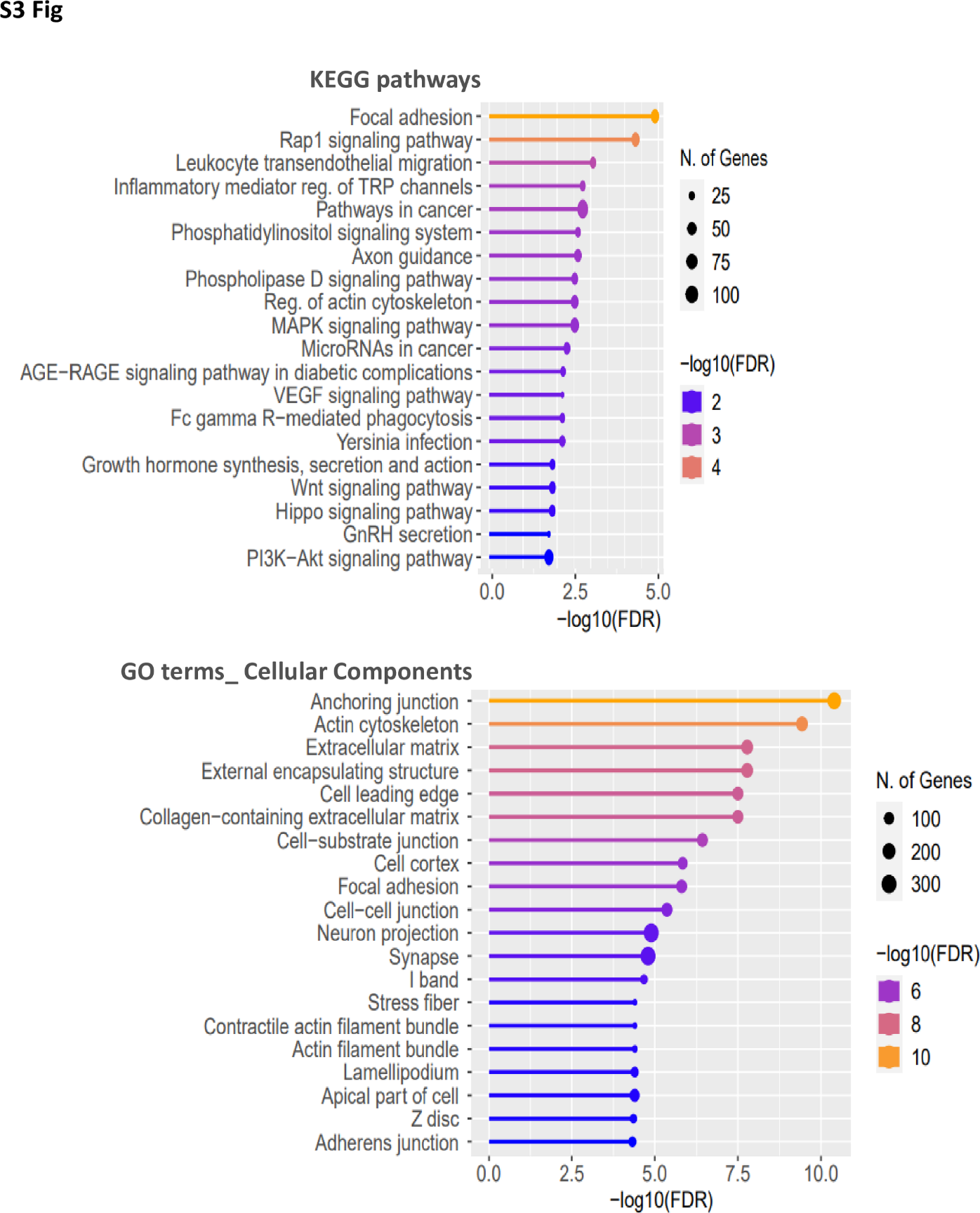


**S4 Figure**. **The representative results of pathway enrichment analysis (KEGG database) and gene ontology (GO) term enrichment analysis ('Cellular Component' category) of genes with revealed variants in coding sequence and genes in which close proximity at least one sequence variant in regulatory element (RE) per patient with KTCN was identified**. On x axes -log(FDR) values of enriched pathways/GO terms, and on y axes the KEGG pathways/GO terms are presented. The size of bubbles indicates the number of genes attributed to particular pathway/GO term and present in our set.

**
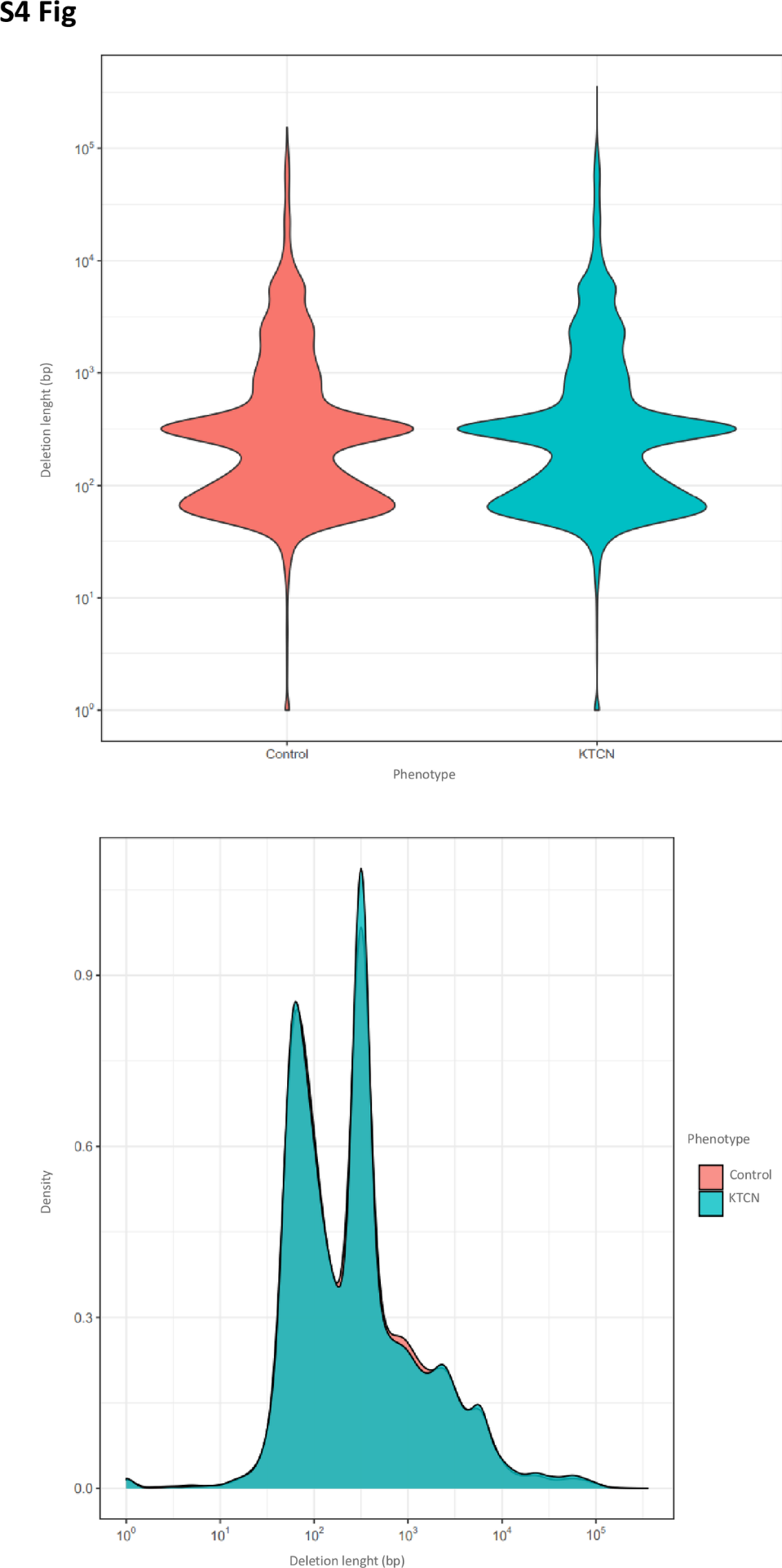
**

**S5 Figure.** **The length and density of deletions revealed in corneal epithelium (CE) samples of patients with KTCN and control individuals.**

**S1 Table.** **The clinical characteristics of the ascertained adolescents patients with KTCN and control individuals (non-KTCN mild myopia patients).** Clinical data concerning both eyes is presented.

| ID | diagnosis | study subgroup | sex | age at examination | age at diagnosis | examined eye | TCT OD [µm] | TCT OS [µm] | AL OD [mm] | AL OS [mm] | IOP OD [mmHg] | IOP OS [mmHg] |
| --- | --- | --- | --- | --- | --- | --- | --- | --- | --- | --- | --- | --- |
| 10 OPT/KTCN | KTCN | ADOLESCENT | M | 14 | 14 | left | 452 | 391 | 24.28 | 24.2 | 15 | 10 |
| 13 OPT/KTCN | KTCN | ADOLESCENT | M | 16 | 16 | right | 489 | 500 | 22.45 | 22.59 | 18 | 17 |
| 18 OPT/KTCN | KTCN | ADOLESCENT | M | 14 | 13 | right | 413 | 318 | 23.71 | 23.62 | 13 | 18 |
| 30 OPT/KTCN | KTCN | ADOLESCENT | F | 13 | 12 | left | 459 | 437 | 24.36 | 24.31 | nd | nd |
| 5 OPT/M | myopia | CONTROL | M | 24 | nd | left | 539 | 531 | 23.68 | 23.31 | nd | nd |
| 6 OPT/M | myopia | CONTROL | M | 30 | nd | right | 489 | 476 | 25.09 | 25.08 | 11 | 15 |

Abbreviations in table: M – male, F – female, OD – right eye, OS – left eye, nd – data not available, n/a – not applicable, TCT – thinnest corneal thickness, AL - Axial length, ^X^-applicable for controls

**S1 Table.** continued

| ID | OD cone/apex^X^ location (central, superior/inferior, nasal/temporal) | OD average thickness of central region-1 [μm] | OD average thickness of middle region-2 [μm] | OD average thickness of periphery region-3 [μm] | OS cone/apex^X^ location (central, superior/inferior, nasal/temporal) | OS average thickness of central region-1 [μm] | OS average thickness of middle region-2 [μm] | OS average thickness of periphery region-3 [μm] | KC grade OD | KC grade OS |
| --- | --- | --- | --- | --- | --- | --- | --- | --- | --- | --- |
| 10 OPT/KTCN | central | 43 | 53 | 46 | Central | 35 | 57 | 47 | 1-2 | 3 |
| 13 OPT/KTCN | inferior, temporal | 42 | 49 | 45 | inferior, temporal | 43 | 51 | 45 | 1-2 | 2 |
| 18 OPT/KTCN | inferior, central | 41 | 47 | 44 | Central | 36 | 49 | 43 | 1-2 | 3 |
| 30 OPT/KTCN | central | 45 | 53 | 46 | central, temporal | 39 | 56 | 47 | 1 | 2-3 |
| 5 OPT/M | central | 51 | 49 | 48 | Central | 52 | 50 | 49 | n/a | n/a |
| 6 OPT/M | central | 49 | 46 | 45 | Central | 49 | 46 | 45 | n/a | n/a |

Abbreviations in table: M – male, F – female, OD – right eye, OS – left eye, nd – data not available, n/a – not applicable, TCT – thinnest corneal thickness, AL - Axial length, ^X^-applicable for controls

**S2 Table.** **Qualitative and quantitative data/results of analyzes of selected behavioral, environmental, and socioeconomic aspects**

| **Variables** | | | **KTCN (n=4)** | **Control (n=2)** |
| --- | --- | --- | --- | --- |
| Age (years), mean±SD | | | 14.3 ± 1.1 | 27.0 ± 3.0 |
| Sex | | |  |  |
|  | Female | | 1 (25.0%) | 0 (0.0%) |
|  | Male | | 3 (75.0%) | 2 (100.0%) |
| Level of education | | |  |  |
|  | Primary | | 4 (100.0%) | 0 (0.0%) |
|  | Vocational education | | 0 (0.0%) | 0 (0.0%) |
|  | High school | | 0 (0.0%) | 1 (50.0%) |
|  | University | | 0 (0.0%) | 1 (50.0%) |
| Place of living up to the age of 15 | | |  |  |
|  | Village | | 1 (25.0%) | 1 (50.0%) |
|  | City up to 20000 residents | | 0 (0.0%) | 0 (0.0%) |
|  | City from 20000 to 100000 residents | | 0 (0.0%) | 1 (50.0%) |
|  | City from 100000 to 500000 residents | | 2 (50.0%) | 0 (0.0%) |
|  | City with over 500000 residents | | 1 (25.0%) | 0 (0.0%) |
| Allergy |  | |  |  |
|  | Yes | | 3 (75.0%) | 1 (50.0%) |
|  | No | | 1 (25.0%) | 1 (50.0%) |
| Food Allergy |  | |  |  |
|  | Yes | | 0 (0.0%) | 0 (0.0%) |
|  | No | | 4 (100.0%) | 2 (100.0%) |
| Pollen/grass/dust Allergy | | |  |  |
|  | Yes | | 3 (75.0%) | 1 (50.0%) |
|  | No | | 1 (25.0%) | 1 (50.0%) |
| Dry eye syndrome (self-reported) | |  |  |  |
|  | Yes | | 1 (25.0%) | 0 (0.0%) |
|  | No | | 3 (75.0%) | 2 (100.0%) |
| Professional occupation |  | |  |  |
|  | Student | | 4 (100.0%) | 0 (0.0%) |
|  | Non-office worker | | 0 (0.0%) | 1 (50.0%) |
|  | Office worker | | 0 (0.0%) | 1 (50.0%) |
| Dust in the working environment | | |  |  |
|  | Yes | | 0 (0.0%) | 1 (50.0%) |
|  | No | | 4 (100.0%) | 1 (50.0%) |
| Eye rubbing |  | |  |  |
|  | Yes | | 4 (100.0%) | 2 (100.0%) |
|  | No | | 0 (0.0%) | 0 (0.0%) |

**S3 Table**. **The results of quantity and quality control**. The results of quantity and quality control of DNA samples (A, Qubit dsDNA HS Assay Kit) and quality control of WGS reads (B).

**A.**

|  | **ID** | **CE region** | **Diagnosis** | **Age** | **Dsph/Dcyl (D)** | **Concentration (ng/µl)** | **Volume (µl)** | **Total amount (ng)** |
| --- | --- | --- | --- | --- | --- | --- | --- | --- |
| 1 | 30 OPT/KTCN/1 | 1 | KTCN | 13 | -1.0/-1.5 | 2.86 | 40 | 114.39 |
| 2 | 30 OPT/KTCN/2 | 2 | KTCN | 13 | -1.0/-1.5 | 3.76 | 40 | 150.39 |
| 3 | 30 OPT/KTCN/3 | 3 | KTCN | 13 | -1.0/-1.5 | 6.1 | 40 | 244 |
| 4 | 18 OPT/KTCN/1 | 1 | KTCN | 14 | +1.0/-1.0 | 2.34 | 40 | 93.6 |
| 5 | 18 OPT/KTCN/2 | 2 | KTCN | 14 | +1.0/-1.0 | 2.7 | 40 | 108 |
| 6 | 18 OPT/KTCN/3 | 3 | KTCN | 14 | +1.0/-1.0 | 6.22 | 40 | 248.79 |
| 7 | 10 OPT/KTCN/1 | 1 | KTCN | 14 | -2.5/-3.0 | 1.2 | 40 | 48 |
| 8 | 10 OPT/KTCN/2 | 2 | KTCN | 14 | -2.5/-3.0 | 3.84 | 40 | 153.6 |
| 9 | 10 OPT/KTCN/3 | 3 | KTCN | 14 | -2.5/-3.0 | 2.56 | 40 | 102.4 |
| 10 | 13 OPT/KTCN/1 | 1 | KTCN | 16 | +1.25/-0.75 | 0.68 | 40 | 27.36 |
| 11 | 13 OPT/KTCN/2 | 2 | KTCN | 16 | +1.25/-0.75 | 3.8 | 40 | 152 |
| 12 | 13 OPT/KTCN/3 | 3 | KTCN | 16 | +1.25/-0.75 | 2.42 | 40 | 96.8 |
| 13 | 5 OPT/M/1 | 1 | Control | 24 | -0.25/- | 3.1 | 40 | 124 |
| 14 | 5 OPT/M/2 | 2 | Control | 24 | -0.25/- | 3.92 | 40 | 156.8 |
| 15 | 5 OPT/M/3 | 3 | Control | 24 | -0.25/- | 3.18 | 40 | 127.2 |
| 16 | 6 OPT/M/1 | 1 | Control | 30 | +0.25/-1.0 | 4.94 | 40 | 197.6 |
| 17 | 6 OPT/M/2 | 2 | Control | 30 | +0.25/-1.0 | 2.68 | 40 | 107.2 |
| 18 | 6 OPT/M/3 | 3 | Control | 30 | +0.25/-1.0 | 3.98 | 40 | 159.2 |

**B.**

|  | **ID** | **CE region** | **Diagnosis** | **Raw data (Gb)** | **Raw Reads (M)** | **Q30 (%)** |
| --- | --- | --- | --- | --- | --- | --- |
| 1 | 30 OPT/KTCN/1 | 1 | KTCN | 114.35 | 762.36 | 94.19 |
| 2 | 30 OPT/KTCN/2 | 2 | KTCN | 104.44 | 696.24 | 94.10 |
| 3 | 30 OPT/KTCN/3 | 3 | KTCN | 108.33 | 722.17 | 94.06 |
| 4 | 18 OPT/KTCN/1 | 1 | KTCN | 102.88 | 685.89 | 93.90 |
| 5 | 18 OPT/KTCN/2 | 2 | KTCN | 108.52 | 723.46 | 94.44 |
| 6 | 18 OPT/KTCN/3 | 3 | KTCN | 112.90 | 752.68 | 94.35 |
| 7 | 10 OPT/KTCN/1 | 1 | KTCN | 107.04 | 713.62 | 94.06 |
| 8 | 10 OPT/KTCN/2 | 2 | KTCN | 100.57 | 670.44 | 94.30 |
| 9 | 10 OPT/KTCN/3 | 3 | KTCN | 102.24 | 681.58 | 93.87 |
| 10 | 13 OPT/KTCN/1 | 1 | KTCN | 98.98 | 659.87 | 94.02 |
| 11 | 13 OPT/KTCN/2 | 2 | KTCN | 111.95 | 746.30 | 93.91 |
| 12 | 13 OPT/KTCN/3 | 3 | KTCN | 102.20 | 681.31 | 93.88 |
| 13 | 5 OPT/M/1 | 1 | Control | 106.55 | 710.33 | 93.89 |
| 14 | 5 OPT/M/2 | 2 | Control | 103.22 | 688.11 | 93.87 |
| 15 | 5 OPT/M/3 | 3 | Control | 116.69 | 777.90 | 94.16 |
| 16 | 6 OPT/M/1 | 1 | Control | 115.40 | 769.35 | 93.88 |
| 17 | 6 OPT/M/2 | 2 | Control | 106.45 | 709.69 | 94.05 |
| 18 | 6 OPT/M/3 | 3 | Control | 90.99 | 606.57 | 93.97 |

**S4 Table. The results of gene ontology (GO) term enrichment analysis for 'Biological Process' (BP), 'Cellular Component' (CC), and 'Molecular Function' (MF) categories**. Analysis embraced genes with variants identified in coding sequence, unique for adolescents patients with KTCN.

| GO_category | GO_term | nGenes | Pathway Genes | Fold Enrichment | FDR | Genes |
| --- | --- | --- | --- | --- | --- | --- |
| BP | Cytoskeleton organization | 68 | 1553 | 1.772914 | 0.013674 | *CCDC88C, RUFY3, INSRR, SYNE2, LLGL2, ARHGEF10L, EPB41L2, FAT1, CASS4, ICAM1, MISP, ARHGEF7, TLE6, ZPR1, JHY, RSPH4A, HRG, SSX2IP, CNTRL, CCDC170, TTLL2, SORBS3, PACSIN1, VIL1, EPB41L4A, NECTIN2, CAMSAP1, CLIP1, HIP1R, MAP1B, ATP8A2, HOOK1, PDGFRA, TTLL4, TLN1, SHROOM3, BRCA2, MINK1, SLIT2, TBCK, IQGAP2, CHEK1, TUBA3E, HAUS1, FARP1, OBSCN, PDLIM3, TTN, CFAP410, NEXN, PDLIM5, SPTA1, SHROOM1, INPPL1, CEP295, MYO1A, EVPL, ULK4, CCDC8, PDE4DIP, ERCC6L, INSC, MYH6, NRAP, DMD, IQANK1, KBTBD13, MAGEL2* |
| BP | Biological adhesion | 70 | 1646 | 1.721942 | 0.013674 | *TNC, LAMC2, ERBB3, COL17A1, SDK2, GPC4, FAT1, CASS4, SIGLEC1, ICAM1, LAMB1, ARHGEF7, PIEZO1, CD276, RASAL3, APLP1, AKNA, DOCK8, CDH23, VWF, HRG, FN1, NID1, SSX2IP, TJP2, SORBS3, NECTIN2, EPO, COL5A1, LAMA5, RIN2, AP3B1, CNTN6, PDGFRA, TLN1, MMRN1, PPFIA2, HAPLN3, MINK1, SIGLEC10, ANXA9, TENM2, TNFRSF21, SDK1, FREM2, SLC7A11, CD96, OBSCN, ITGAD, NEXN, PDLIM5, CCDC141, SPTA1, ADGRV1, INPPL1, B4GALNT2, LRRC15, EFEMP2, HPSE, FUT7, MUC16, FAF1, AMTN, DMD, PECAM1, PCDHB16, MUC4, MAGI1, DCHS2, BCAR1* |
| BP | Cell adhesion | 69 | 1639 | 1.704592 | 0.014974 | *TNC, LAMC2, ERBB3, COL17A1, SDK2, GPC4, FAT1, CASS4, SIGLEC1, ICAM1, LAMB1, ARHGEF7, PIEZO1, CD276, RASAL3, APLP1, AKNA, DOCK8, CDH23, VWF, HRG, FN1, NID1, SSX2IP, TJP2, SORBS3, NECTIN2, EPO, COL5A1, LAMA5, RIN2, AP3B1, CNTN6, PDGFRA, TLN1, MMRN1, PPFIA2, HAPLN3, MINK1, SIGLEC10, ANXA9, TENM2, TNFRSF21, SDK1, FREM2, SLC7A11, CD96, OBSCN, ITGAD, NEXN, PDLIM5, CCDC141, SPTA1, ADGRV1, INPPL1, B4GALNT2, EFEMP2, HPSE, FUT7, MUC16, FAF1, AMTN, DMD, PECAM1, PCDHB16, MUC4, MAGI1, DCHS2, BCAR1* |
| BP | Actin filament-based process | 41 | 842 | 1.971615 | 0.036246 | *CCDC88C, RUFY3, INSRR, SYNE2, LLGL2, ARHGEF10L, EPB41L2, FAT1, CASS4, ICAM1, TNNC2, TLE6, HRG, SORBS3, PACSIN1, VIL1, EPB41L4A, HIP1R, PDGFRA, TLN1, SHROOM3, MINK1, SLIT2, TBCK, IQGAP2, FARP1, OBSCN, PDLIM3, TTN, KCNE2, PDLIM5, SPTA1, SHROOM1, INPPL1, MYO1A, MYH6, NRAP, DMD, IQANK1, KBTBD13, MAGEL2* |
| BP | Actin cytoskeleton organization | 37 | 744 | 2.013627 | 0.039155 | *RUFY3, INSRR, LLGL2, ARHGEF10L, EPB41L2, FAT1, CASS4, ICAM1, TLE6, HRG, SORBS3, PACSIN1, VIL1, EPB41L4A, HIP1R, PDGFRA, TLN1, SHROOM3, MINK1, SLIT2, TBCK, IQGAP2, FARP1, OBSCN, PDLIM3, TTN, PDLIM5, SPTA1, SHROOM1, INPPL1, MYO1A, MYH6, NRAP, DMD, IQANK1, KBTBD13, MAGEL2* |
| BP | Supramolecular fiber organization | 43 | 919 | 1.894537 | 0.039155 | *CCDC88C, RUFY3, COL23A1, LAMC2, SNCAIP, COL17A1, ARHGEF10L, FAT1, CASS4, ICAM1, ARHGEF7, TLE6, AEBP1, P3H1, SORBS3, IAPP, PACSIN1, VIL1, COLGALT1, CAMSAP1, COL5A1, CLIP1, HIP1R, MAP1B, HOOK1, PDGFRA, SHROOM3, SLIT2, IQGAP2, OBSCN, TTN, SPTA1, SHROOM1, INPPL1, MYO1A, EFEMP2, PDE4DIP, MYH6, NRAP, DMD, IQANK1, KBTBD13, MAGEL2* |
| BP | Protein localization to endoplasmic reticulum exit site | 4 | 10 | 16.19609 | 0.045724 | *SEC16A, MIA2, BCAP29, DUS4L-BCAP29* |
| BP | Plasma membrane bounded cell projection organization | 65 | 1649 | 1.596037 | 0.073568 | *HECW1, MAPK8IP2, RUFY3, TNC, SYNE2, LAMC2, SEMA3C, RIMS1, ICAM1, LAMB1, KIAA0586, ZMYND8, ARHGEF7, ANKRD27, PTPRZ1, CDH23, ZPR1, JHY, RSPH4A, HRG, FN1, EXOC8, MTR, SSX2IP, P3H1, CNTRL, TTC21B, PACSIN1, VIL1, NECTIN2, EPO, OLFM1, CAMSAP1, LAMA5, MAP1B, ATP8A2, CNTN6, PPFIA2, MINK1, RNF157, CPNE9, SLIT2, TENM2, TNFRSF21, SDK1, HAUS1, FARP1, CFAP410, NEXN, PDLIM5, CCDC141, SPTA1, ADGRV1, INPPL1, CLMN, MYO1A, ULK4, ROR2, ARL13B, SFI1, CEP290, DMD, SNX2, SKOR2, ERCC6* |
| BP | Cell projection organization | 66 | 1690 | 1.581275 | 0.075466 | *HECW1, DNAH9, MAPK8IP2, RUFY3, TNC, SYNE2, LAMC2, SEMA3C, RIMS1, ICAM1, LAMB1, KIAA0586, ZMYND8, ARHGEF7, ANKRD27, PTPRZ1, CDH23, ZPR1, JHY, RSPH4A, HRG, FN1, EXOC8, MTR, SSX2IP, P3H1, CNTRL, TTC21B, PACSIN1, VIL1, NECTIN2, EPO, OLFM1, CAMSAP1, LAMA5, MAP1B, ATP8A2, CNTN6, PPFIA2, MINK1, RNF157, CPNE9, SLIT2, TENM2, TNFRSF21, SDK1, HAUS1, FARP1, CFAP410, NEXN, PDLIM5, CCDC141, SPTA1, ADGRV1, INPPL1, CLMN, MYO1A, ULK4, ROR2, ARL13B, SFI1, CEP290, DMD, SNX2, SKOR2, ERCC6* |
| BP | Inner cell mass cell proliferation | 4 | 14 | 11.56864 | 0.128189 | *SALL4, ZPR1, BRCA2, CHEK1* |
| BP | Regulation of collagen fibril organization | 3 | 6 | 20.24512 | 0.128189 | *AEBP1, COLGALT1, EFEMP2* |
| CC | External encapsulating structure | 37 | 601 | 2.4927 | 0.0001 | *TNC, COL23A1, LAMC2, COL17A1, GPC4, ICAM1, LAMB1, MXRA5, APLP1, PTPRZ1, AEBP1, VWF, HRG, FN1, NID1, P3H1, MUC5B, A1BG, COL5A1, LAMA5, ENAM, MMRN1, PCSK6, HAPLN3, ECM1, FLG, FREM2, CPA3, UCMA, NAV2, MUC17, LRRC15, EFEMP2, HPSE, AMTN, OTOG, MUC5AC* |
| CC | Extracellular matrix | 37 | 600 | 2.4969 | 0.0001 | *TNC, COL23A1, LAMC2, COL17A1, GPC4, ICAM1, LAMB1, MXRA5, APLP1, PTPRZ1, AEBP1, VWF, HRG, FN1, NID1, P3H1, MUC5B, A1BG, COL5A1, LAMA5, ENAM, MMRN1, PCSK6, HAPLN3, ECM1, FLG, FREM2, CPA3, UCMA, NAV2, MUC17, LRRC15, EFEMP2, HPSE, AMTN, OTOG, MUC5AC* |
| CC | Collagen-containing extracellular matrix | 30 | 446 | 2.7236 | 0.0002 | *TNC, COL23A1, LAMC2, COL17A1, GPC4, ICAM1, LAMB1, MXRA5, APLP1, PTPRZ1, AEBP1, VWF, HRG, FN1, NID1, P3H1, A1BG, COL5A1, LAMA5, MMRN1, PCSK6, ECM1, FLG, FREM2, CPA3, NAV2, MUC17, LRRC15, EFEMP2, AMTN* |
| CC | Basement membrane | 12 | 97 | 5.0091 | 0.0008 | *TNC, LAMC2, COL17A1, LAMB1, APLP1, FN1, NID1, COL5A1, LAMA5, FREM2, EFEMP2, AMTN* |
| CC | Supramolecular fiber | 50 | 1102 | 1.8371 | 0.0034 | *DNAH9, SYNE2, KIF26A, SCTR, MISP, CEP170B, TNNC2, DNAH11, AKNA, RMDN2, KIF1C, MTUS1, CAMSAP1, COL5A1, CLIP1, SYNE1, MAP1B, MYO18B, HOOK1, TTLL4, SHROOM3, MYOM3, FLG, MYH15, IQGAP2, TUBA3E, HAUS1, OBSCN, PDLIM3, TTN, DNAH3, SQSTM1, NEXN, PDLIM5, SHROOM1, CEP295, MYO1A, EVPL, ROR2, EFEMP2, PDE4DIP, DNAH2, AHNAK2, KRT35, MYH6, NRAP, DMD, EML6, KRT33B, KRTAP10-7* |
| CC | Supramolecular polymer | 50 | 1110 | 1.8239 | 0.0034 | *DNAH9, SYNE2, KIF26A, SCTR, MISP, CEP170B, TNNC2, DNAH11, AKNA, RMDN2, KIF1C, MTUS1, CAMSAP1, COL5A1, CLIP1, SYNE1, MAP1B, MYO18B, HOOK1, TTLL4, SHROOM3, MYOM3, FLG, MYH15, IQGAP2, TUBA3E, HAUS1, OBSCN, PDLIM3, TTN, DNAH3, SQSTM1, NEXN, PDLIM5, SHROOM1, CEP295, MYO1A, EVPL, ROR2, EFEMP2, PDE4DIP, DNAH2, AHNAK2, KRT35, MYH6, NRAP, DMD, EML6, KRT33B, KRTAP10-7* |
| CC | Microtubule | 26 | 464 | 2.2688 | 0.0090 | *DNAH9, KIF26A, SCTR, MISP, CEP170B, DNAH11, AKNA, RMDN2, KIF1C, MTUS1, CAMSAP1, CLIP1, MAP1B, HOOK1, TTLL4, SHROOM3, IQGAP2, TUBA3E, HAUS1, DNAH3, SHROOM1, CEP295, ROR2, PDE4DIP, DNAH2, EML6* |
| CC | Myofibril | 17 | 244 | 2.8210 | 0.0101 | *SYNE2, TNNC2, SYNE1, MYO18B, MYOM3, MYH15, OBSCN, PDLIM3, TTN, SQSTM1, NEXN, PDLIM5, PDE4DIP, AHNAK2, MYH6, NRAP, DMD* |
| CC | Contractile fiber | 17 | 253 | 2.7207 | 0.0138 | *SYNE2, TNNC2, SYNE1, MYO18B, MYOM3, MYH15, OBSCN, PDLIM3, TTN, SQSTM1, NEXN, PDLIM5, PDE4DIP, AHNAK2, MYH6, NRAP, DMD* |
| CC | Cortical cytoskeleton | 11 | 127 | 3.5070 | 0.0198 | *LLGL2, EPB41L2, RIMS1, MISP, HIP1R, SHROOM3, SPTA1, SHROOM1, PRKCB, MYO1A, PDE4DIP* |
| CC | Sarcomere | 15 | 221 | 2.7482 | 0.0237 | *SYNE2, TNNC2, SYNE1, MYO18B, MYOM3, OBSCN, PDLIM3, TTN, SQSTM1, NEXN, PDLIM5, AHNAK2, MYH6, NRAP, DMD* |
| CC | Z disc | 11 | 136 | 3.2749 | 0.0296 | *SYNE2, MYO18B, OBSCN, PDLIM3, TTN, NEXN, PDLIM5, AHNAK2, MYH6, NRAP, DMD* |
| CC | Cortical actin cytoskeleton | 9 | 98 | 3.7185 | 0.0344 | *LLGL2, EPB41L2, MISP, HIP1R, SHROOM3, SPTA1, SHROOM1, PRKCB, MYO1A* |
| CC | Spectrin | 3 | 9 | 13.4967 | 0.0478 | *EPB41L2, SPTA1, PRKCB* |
| CC | I band | 11 | 148 | 3.0094 | 0.0478 | *SYNE2, MYO18B, OBSCN, PDLIM3, TTN, NEXN, PDLIM5, AHNAK2, MYH6, NRAP, DMD* |
| MF | Cytoskeletal protein binding | 59 | 1050 | 2.2752 | 0.0000 | *VPS41, MAPK8IP2, CCDC88C, SYNE2, KIF26A, LLGL2, TNS1, EPB41L2, MISP, TNNC2, ARHGEF7, RMDN2, CNTRL, CCDC170, TTLL2, SORBS3, PACSIN1, VIL1, KIF1C, MTUS1, EPB41L4A, CAMSAP1, CLIP1, HIP1R, SYNE1, MAP1B, MYO18B, HOOK1, TTLL4, TLN1, SHROOM3, BRCA2, MYOM3, MYH15, IQGAP2, FARP1, OBSCN, PDLIM3, TTN, PIP, NEXN, PDLIM5, SPTA1, SHROOM1, INPPL1, CLMN, CEP295, MYO1A, JAKMIP2, MX2, MRTFB, MYH6, NRAP, DMD, KIFBP, EML6, KBTBD13, MYO15B, MAGI1* |
| MF | Actin binding | 32 | 477 | 2.7163 | 0.0001 | *SYNE2, TNS1, EPB41L2, MISP, TNNC2, VIL1, HIP1R, SYNE1, MAP1B, MYO18B, HOOK1, TLN1, SHROOM3, MYOM3, MYH15, IQGAP2, PDLIM3, TTN, PIP, NEXN, PDLIM5, SPTA1, SHROOM1, INPPL1, CLMN, MYO1A, MRTFB, MYH6, NRAP, DMD, KBTBD13, MYO15B* |
| MF | Actin filament binding | 21 | 229 | 3.7131 | 0.0001 | *SYNE2, MISP, TNNC2, VIL1, HIP1R, SYNE1, TLN1, SHROOM3, MYOM3, MYH15, IQGAP2, TTN, NEXN, SPTA1, SHROOM1, CLMN, MYO1A, MYH6, NRAP, DMD, KBTBD13* |
| MF | Extracellular matrix structural constituent | 18 | 186 | 3.9184 | 0.0002 | *TNC, COL23A1, LAMC2, COL17A1, LAMB1, MXRA5, AEBP1, VWF, FN1, NID1, COL5A1, LAMA5, ENAM, MMRN1, ECM1, MUC17, EFEMP2, MUC5AC* |
| MF | Dynein light intermediate chain binding | 7 | 29 | 9.7735 | 0.0010 | *DNAH9, CCDC88C, DNAH11, HOOK1, DNAH3, DNHD1, DNAH2* |
| MF | Proteoglycan binding | 7 | 38 | 7.4587 | 0.0053 | *TNC, HRG, FN1, NID1, COL5A1, SLIT2, HPSE* |
| MF | Cytoskeletal motor activity | 12 | 124 | 3.9184 | 0.0076 | *DNAH9, KIF26A, DNAH11, KIF1C, MYO18B, MYH15, DNAH3, MYO1A, DNHD1, DNAH2, MYH6, MYO15B* |
| MF | Structural molecule activity | 38 | 784 | 1.9625 | 0.0076 | *MAPK8IP2, TNC, COL23A1, LAMC2, COL17A1, EPB41L2, LAMB1, MXRA5, AEBP1, VWF, FN1, NID1, SORBS3, COL5A1, LAMA5, MAP1B, ENAM, TLN1, MMRN1, PPFIA2, ECM1, FLG, MRPS5, TUBA3E, OBSCN, TTN, NEXN, SPTA1, EVPL, MUC17, EFEMP2, OTOG, CRYBA4, KRT35, DMD, KRT33B, POM121C, MUC5AC* |
| MF | Minus-end-directed microtubule motor activity | 5 | 19 | 10.6553 | 0.0080 | *DNAH9, DNAH11, DNAH3, DNHD1, DNAH2* |
| MF | ATP binding | 65 | 1662 | 1.5836 | 0.0125 | *DNAH9, INSRR, FLT4, PNKP, ERBB3, KARS1, KIF26A, FGFR3, TRIB3, GUCY2F, TIMM44, LIG1, DNAH11, ABCC3, RARS1, PASK, TTLL2, ABCC11, WNK4, TRAP1, KIF1C, CCT6B, ATP8A2, MYO18B, PDGFRA, TTLL4, SMC2, CDKL2, TDG, UBE2Q2, MINK1, PFKL, MYH15, TBCK, CACNA1B, CHEK1, ABCB9, ATP5F1A, OBSCN, TTN, DNAH3, FGFR4, NEK10, PRKCB, NAV2, MYO1A, ULK4, ROR2, TDRD12, MAP3K19, CHD9, DNHD1, DNAH2, ERCC6L, SLC22A5, MYH6, ATAD3A, TLK1, ATAD3C, ERCC6, CDK11B, CSNK2A3, MYO15B, PANK4, MAGI1* |
| MF | Vinculin binding | 4 | 12 | 13.4967 | 0.0125 | *SORBS3, TLN1, NRAP, DMD* |
| MF | Adenyl ribonucleotide binding | 67 | 1730 | 1.5681 | 0.0125 | *DNAH9, INSRR, FLT4, PNKP, ERBB3, KARS1, KIF26A, FGFR3, ACADVL, HCN2, TRIB3, GUCY2F, TIMM44, LIG1, DNAH11, ABCC3, RARS1, PASK, TTLL2, ABCC11, WNK4, TRAP1, KIF1C, CCT6B, ATP8A2, MYO18B, PDGFRA, TTLL4, SMC2, CDKL2, TDG, UBE2Q2, MINK1, PFKL, MYH15, TBCK, CACNA1B, CHEK1, ABCB9, ATP5F1A, OBSCN, TTN, DNAH3, FGFR4, NEK10, PRKCB, NAV2, MYO1A, ULK4, ROR2, TDRD12, MAP3K19, CHD9, DNHD1, DNAH2, ERCC6L, SLC22A5, MYH6, ATAD3A, TLK1, ATAD3C, ERCC6, CDK11B, CSNK2A3, MYO15B, PANK4, MAGI1* |
| MF | Adenyl nucleotide binding | 67 | 1743 | 1.5564 | 0.0143 | *DNAH9, INSRR, FLT4, PNKP, ERBB3, KARS1, KIF26A, FGFR3, ACADVL, HCN2, TRIB3, GUCY2F, TIMM44, LIG1, DNAH11, ABCC3, RARS1, PASK, TTLL2, ABCC11, WNK4, TRAP1, KIF1C, CCT6B, ATP8A2, MYO18B, PDGFRA, TTLL4, SMC2, CDKL2, TDG, UBE2Q2, MINK1, PFKL, MYH15, TBCK, CACNA1B, CHEK1, ABCB9, ATP5F1A, OBSCN, TTN, DNAH3, FGFR4, NEK10, PRKCB, NAV2, MYO1A, ULK4, ROR2, TDRD12, MAP3K19, CHD9, DNHD1, DNAH2, ERCC6L, SLC22A5, MYH6, ATAD3A, TLK1, ATAD3C, ERCC6, CDK11B, CSNK2A3, MYO15B, PANK4, MAGI1* |
| MF | Muscle alpha-actinin binding | 4 | 15 | 10.7974 | 0.0264 | *PDLIM3, TTN, PDLIM5, NRAP* |
| MF | Heparin binding | 13 | 184 | 2.8607 | 0.0391 | *LAMC2, LXN, APOB, APLP1, HRG, FN1, COL5A1, PCSK6, SLIT2, FGFR4, TMEM184A, NAV2, EFEMP2* |
| MF | Dynein intermediate chain binding | 5 | 29 | 6.9811 | 0.0391 | *DNAH9, DNAH11, DNAH3, DNHD1, DNAH2* |
| MF | Alpha-actinin binding | 5 | 30 | 6.7484 | 0.0415 | *PDLIM3, TTN, PDLIM5, NRAP, MAGI1* |

| Pathway identifier | Pathway name | #Entities found | #Entities total | Entities pvalue | Entities FDR |
| --- | --- | --- | --- | --- | --- |
| R-HSA-1236977 | Endosomal/Vacuolar pathway | 21 | 82 | 0.0000 | <0.0001 |
| R-HSA-983170 | Antigen Presentation: Folding, assembly and peptide loading of class I MHC | 21 | 108 | 0.0000 | 0.0001 |
| R-HSA-198933 | Immunoregulatory interactions between a Lymphoid and a non-Lymphoid cell | 38 | 316 | 0.0000 | 0.0001 |
| R-HSA-909733 | Interferon alpha/beta signaling | 27 | 190 | 0.0000 | 0.0002 |
| R-HSA-1236974 | ER-Phagosome pathway | 22 | 173 | 0.0000 | 0.0089 |
| R-HSA-1236975 | Antigen processing-Cross presentation | 22 | 195 | 0.0002 | 0.0381 |
| R-HSA-877300 | Interferon gamma signaling | 26 | 252 | 0.0002 | 0.0381 |
| R-HSA-9705671 | SARS-CoV-2 activates/modulates innate and adaptive immune responses | 24 | 227 | 0.0003 | 0.0404 |
| R-HSA-913531 | Interferon Signaling | 35 | 397 | 0.0004 | 0.0523 |
| R-HSA-9705683 | SARS-CoV-2-host interactions | 27 | 315 | 0.0027 | 0.2679 |
| R-HSA-5083636 | Defective GALNT12 causes CRCS1 | 5 | 20 | 0.0029 | 0.2679 |
| R-HSA-5083625 | Defective GALNT3 causes HFTC | 5 | 20 | 0.0029 | 0.2679 |
| R-HSA-5083632 | Defective C1GALT1C1 causes TNPS | 5 | 21 | 0.0035 | 0.3012 |
| R-HSA-373756 | SDK interactions | 2 | 2 | 0.0042 | 0.3347 |
| R-HSA-190374 | FGFR1c and Klotho ligand binding and activation | 3 | 7 | 0.0047 | 0.3519 |
| R-HSA-109704 | PI3K Cascade | 8 | 58 | 0.0071 | 0.4658 |
| R-HSA-216083 | Integrin cell surface interactions | 10 | 86 | 0.0088 | 0.5383 |
| R-HSA-5624958 | ARL13B-mediated ciliary trafficking of INPP5E | 2 | 3 | 0.0091 | 0.5383 |
| R-HSA-977068 | Termination of O-glycan biosynthesis | 5 | 28 | 0.0113 | 0.6336 |
| R-HSA-112399 | IRS-mediated signalling | 8 | 65 | 0.0133 | 0.6769 |
| R-HSA-2219530 | Constitutive Signaling by Aberrant PI3K in Cancer | 10 | 96 | 0.0175 | 0.7578 |
| R-HSA-2428928 | IRS-related events triggered by IGF1R | 8 | 69 | 0.0182 | 0.7578 |
| R-HSA-190242 | FGFR1 ligand binding and activation | 4 | 21 | 0.0184 | 0.7578 |
| R-HSA-8874081 | MET activates PTK2 signaling | 5 | 32 | 0.0190 | 0.7578 |
| R-HSA-372708 | p130Cas linkage to MAPK signaling for integrins | 4 | 22 | 0.0214 | 0.7578 |
| R-HSA-5654219 | Phospholipase C-mediated cascade: FGFR1 | 4 | 22 | 0.0214 | 0.7578 |
| R-HSA-2428924 | IGF1R signaling cascade | 8 | 72 | 0.0228 | 0.7578 |
| R-HSA-74751 | Insulin receptor signalling cascade | 8 | 72 | 0.0228 | 0.7578 |
| R-HSA-1839120 | Signaling by FGFR1 amplification mutants | 2 | 5 | 0.0238 | 0.7578 |
| R-HSA-2404192 | Signaling by Type 1 Insulin-like Growth Factor 1 Receptor (IGF1R) | 8 | 73 | 0.0244 | 0.7578 |
| R-HSA-1474244 | Extracellular matrix organization | 24 | 328 | 0.0255 | 0.7578 |
| R-HSA-3000171 | Non-integrin membrane-ECM interactions | 7 | 61 | 0.0276 | 0.7578 |
| R-HSA-983169 | Class I MHC mediated antigen processing & presentation | 32 | 479 | 0.0340 | 0.7578 |
| R-HSA-2219528 | PI3K/AKT Signaling in Cancer | 11 | 124 | 0.0363 | 0.7578 |
| R-HSA-1226099 | Signaling by FGFR in disease | 8 | 82 | 0.0434 | 0.7578 |
| R-HSA-5654689 | PI-3K cascade:FGFR1 | 4 | 28 | 0.0452 | 0.7578 |

**S5 Table. Results of pathway enrichment analysis of genes with variants in coding sequence, unique for adolescent patients with KTCN**. The Reactome database was chosen for definition of pathways.

**S6 Table. Results of STRING protein-protein interaction analysis of genes with variants identified in coding sequence, unique for adolescent patients with KTCN.**

| **#cluster ID** | **Cluster description** | **Observed gene count** | **Background gene count** | **Strength** | **FDR** | **Matching proteins in your network (labels)** |
| --- | --- | --- | --- | --- | --- | --- |
| CL:16428 | Mixed, incl. Collagen formation, and Defective B3GALTL causes Peters-plus syndrome (PpS) | 37 | 187 | 1.04 | 1.43e-21 | DCN, SERPINE1, VTN, LOX, LEPRE1, COL21A1, ADAMTS2, COLGALT1, LOXL4, COL8A1, P4HA1, NID1, PLOD2, COL6A3, COL14A1, COL12A1, FBN1, COL27A1, TLL2, ELN, MMP17, COLGALT2, COL5A1, MATN1, HSPG2, COL5A2, ASPN, LOXL2, COL23A1, COL13A1, COL25A1, COL28A1, LTBP1, ACAN, CD44, ADAMTS9, FBN2 |
| CL:16429 | Collagen formation, and Defective B3GALTL causes Peters-plus syndrome (PpS) | 35 | 168 | 1.06 | 3.30e-21 | DCN, SERPINE1, VTN, LOX, LEPRE1, COL21A1, ADAMTS2, COLGALT1, LOXL4, COL8A1, P4HA1, NID1, PLOD2, COL6A3, COL14A1, COL12A1, FBN1, COL27A1, TLL2, ELN, MMP17, COLGALT2, COL5A1, HSPG2, COL5A2, LOXL2, COL23A1, COL13A1, COL25A1, COL28A1, LTBP1, ACAN, CD44, ADAMTS9, FBN2 |
| CL:16430 | Collagen formation, and Matrix metalloproteinases | 32 | 129 | 1.14 | 3.30e-21 | DCN, SERPINE1, VTN, LOX, LEPRE1, COL21A1, ADAMTS2, COLGALT1, LOXL4, COL8A1, P4HA1, NID1, PLOD2, COL6A3, COL14A1, COL12A1, FBN1, COL27A1, TLL2, ELN, MMP17, COLGALT2, COL5A1, HSPG2, COL5A2, LOXL2, COL23A1, COL13A1, COL25A1, COL28A1, CD44, FBN2 |
| CL:16804 | Mixed, incl. protein complex involved in cell adhesion, and MET activates PTK2 signaling | 21 | 68 | 1.23 | 4.27e-15 | LAMB1, LAMA5, LAMC1, ITGAV, ITGA2B, LAMC2, ICAM1, ITGB5, JAM3, PTPN2, LIMS2, ITGA11, PARVA, FERMT2, FN1, ITGAD, LAMA1, ITGA4, PARVB, LAMA2, LIMS1 |
| CL:16802 | Mixed, incl. protein complex involved in cell adhesion, and substrate adhesion-dependent cell spreading | 22 | 82 | 1.17 | 6.73e-15 | LAMB1, LAMA5, LAMC1, ITGAV, ITGA2B, LAMC2, ICAM1, ITGB5, JAM3, PTPN2, LIMS2, ITGA11, PARVA, FERMT2, FN1, ITGA8, ITGAD, LAMA1, ITGA4, PARVB, LAMA2, LIMS1 |
| CL:16432 | Collagen biosynthesis and modifying enzymes | 18 | 66 | 1.18 | 3.43e-12 | LEPRE1, COL21A1, ADAMTS2, COLGALT1, COL8A1, P4HA1, PLOD2, COL6A3, COL14A1, COL27A1, TLL2, COLGALT2, COL5A1, COL5A2, COL23A1, COL13A1, COL25A1, COL28A1 |
| CL:16433 | Collagen biosynthesis and modifying enzymes | 17 | 57 | 1.22 | 5.06e-12 | COL21A1, ADAMTS2, COLGALT1, COL8A1, P4HA1, PLOD2, COL6A3, COL14A1, COL27A1, TLL2, COLGALT2, COL5A1, COL5A2, COL23A1, COL13A1, COL25A1, COL28A1 |
| CL:16805 | Protein complex involved in cell adhesion, and MET activates PTK2 signaling | 16 | 57 | 1.19 | 6.20e-11 | LAMB1, LAMA5, LAMC1, ITGAV, ITGA2B, LAMC2, ICAM1, ITGB5, JAM3, PTPN2, ITGA11, FN1, ITGAD, LAMA1, ITGA4, LAMA2 |
| CL:21040 | Wnt signaling pathway, and beta-catenin-TCF complex assembly | 18 | 98 | 1.01 | 8.84e-10 | BCL9, APC, AXIN1, LGR5, RSPO2, WNT3A, FZD1, LRP5, AXIN2, GSK3B, DACT1, VANGL2, ROR1, ROR2, LGR4, DAAM1, WNT5B, NLK |
| CL:16569 | Mixed, incl. Elastic fibre formation, and Matrix metalloproteinases | 14 | 58 | 1.13 | 1.00e-08 | DCN, SERPINE1, VTN, LOX, LOXL4, NID1, COL12A1, FBN1, ELN, MMP17, HSPG2, LOXL2, CD44, FBN2 |
| CL:16434 | Collagen biosynthesis and modifying enzymes | 12 | 38 | 1.24 | 1.79e-08 | COL21A1, COLGALT1, COL8A1, P4HA1, PLOD2, COL6A3, COL14A1, COLGALT2, COL23A1, COL13A1, COL25A1, COL28A1 |
| CL:21041 | ncRNAs involved in Wnt signaling in hepatocellular carcinoma, and beta-catenin-TCF complex assembly | 15 | 86 | 0.99 | 8.67e-08 | BCL9, APC, AXIN1, WNT3A, FZD1, LRP5, AXIN2, GSK3B, DACT1, VANGL2, ROR1, ROR2, DAAM1, WNT5B, NLK |
| CL:19380 | Mixed, incl. Interferon alpha/beta signaling, and negative regulation of type I interferon production | 14 | 73 | 1.03 | 1.07e-07 | IRF1, GBP7, ISG20, MX2, XAF1, GBP5, GBP1, IRF2, HERC4, IFITM3, OAS1, IFI35, RNF213, IFI27 |
| CL:16436 | Collagen biosynthesis and modifying enzymes | 10 | 27 | 1.31 | 1.59e-07 | COL21A1, COLGALT1, P4HA1, PLOD2, COL6A3, COL14A1, COLGALT2, COL23A1, COL13A1, COL25A1 |
| CL:16437 | Collagen biosynthesis and modifying enzymes | 9 | 22 | 1.36 | 5.20e-07 | COL21A1, COLGALT1, P4HA1, PLOD2, COL14A1, COLGALT2, COL23A1, COL13A1, COL25A1 |
| CL:16868 | Integrin domain superfamily, and hematopoietic stem cell migration to bone marrow | 9 | 25 | 1.3 | 1.23e-06 | ITGAV, ITGA2B, ICAM1, ITGB5, JAM3, ITGA11, FN1, ITGAD, ITGA4 |
| CL:21042 | ncRNAs involved in Wnt signaling in hepatocellular carcinoma, and beta-catenin destruction complex | 13 | 77 | 0.97 | 1.37e-06 | APC, AXIN1, WNT3A, FZD1, LRP5, AXIN2, GSK3B, DACT1, VANGL2, ROR1, ROR2, DAAM1, WNT5B |
| CL:19382 | Mixed, incl. Interferon alpha/beta signaling, and Negative regulators of DDX58/IFIH1 signaling | 12 | 67 | 1.0 | 2.81e-06 | IRF1, ISG20, MX2, XAF1, GBP5, GBP1, HERC4, IFITM3, OAS1, IFI35, RNF213, IFI27 |
| CL:19389 | Interferon alpha/beta signaling, and ISG15-protein conjugation | 10 | 42 | 1.12 | 4.23e-06 | IRF1, ISG20, MX2, XAF1, GBP1, IFITM3, OAS1, IFI35, RNF213, IFI27 |
| CL:19394 | Interferon alpha/beta signaling | 8 | 21 | 1.33 | 5.17e-06 | IRF1, ISG20, MX2, XAF1, IFITM3, OAS1, IFI35, IFI27 |
| CL:19391 | Interferon alpha/beta signaling, and ISG15-protein conjugation | 9 | 36 | 1.14 | 1.40e-05 | IRF1, ISG20, MX2, XAF1, GBP1, IFITM3, OAS1, IFI35, IFI27 |
| CL:22033 | Cell-cell junction assembly, and gap junction | 14 | 120 | 0.81 | 1.97e-05 | PKP1, CLDN16, CDH6, ITGB7, CTNNA1, DST, PKP3, COL17A1, CLDN20, KAZN, PKP4, CLDN6, CLDN8, CLDN2 |
| CL:16870 | Integrin domain superfamily, and hematopoietic stem cell migration to bone marrow | 7 | 18 | 1.33 | 3.12e-05 | ITGAV, ICAM1, JAM3, ITGA11, FN1, ITGAD, ITGA4 |
| CL:16810 | Laminin IV, and Laminin IV type B | 6 | 13 | 1.41 | 0.00010 | LAMB1, LAMA5, LAMC1, LAMC2, LAMA1, LAMA2 |
| CL:21294 | Signaling by BMP, and TGFBR1 KD Mutants in Cancer | 7 | 27 | 1.16 | 0.00027 | TGFB3, SMURF2, SMAD6, SMAD4, SMURF1, NEDD4L, SMAD1 |
| CL:22035 | Mixed, incl. Cadherin, cytoplasmic domain, and Plakophilin/Delta catenin | 9 | 56 | 0.95 | 0.00030 | PKP1, CDH6, ITGB7, CTNNA1, DST, PKP3, COL17A1, KAZN, PKP4 |
| CL:16642 | Elastic fibre formation | 6 | 19 | 1.24 | 0.00054 | LOX, LOXL4, FBN1, ELN, LOXL2, FBN2 |
| CL:21044 | ncRNAs involved in Wnt signaling in hepatocellular carcinoma, and PET prickle | 9 | 61 | 0.91 | 0.00054 | WNT3A, FZD1, LRP5, DACT1, VANGL2, ROR1, ROR2, DAAM1, WNT5B |
| CL:16918 | Cell-extracellular matrix interactions, and negative regulation of timing of anagen | 5 | 11 | 1.4 | 0.00081 | LIMS2, PARVA, FERMT2, PARVB, LIMS1 |
| CL:21296 | Type II transforming growth factor beta receptor binding, and Dwarfin | 5 | 12 | 1.36 | 0.0011 | TGFB3, SMURF2, SMAD6, SMURF1, SMAD1 |
| CL:16570 | Mixed, incl. Matrix metalloproteinases, and Dissolution of Fibrin Clot | 7 | 36 | 1.03 | 0.0012 | SERPINE1, VTN, NID1, COL12A1, MMP17, HSPG2, CD44 |
| CL:21310 | Dwarfin, and engulfment of target by autophagosome | 4 | 5 | 1.65 | 0.0013 | SMURF2, SMAD6, SMURF1, SMAD1 |
| CL:5083 | Anaphase-promoting complex | 5 | 13 | 1.33 | 0.0013 | ANAPC5, ANAPC1, ANAPC13, CDC27, ANAPC10 |
| CL:2688 | Mixed, incl. proteasome complex, and Ubiquitin-conjugating enzyme | 13 | 161 | 0.65 | 0.0015 | UBE2K, PSMB1, PSMD3, UBE2Q2, PSMA5, UBE3C, PSMD1, UBA6, UBE2V1, UBE2H, KCMF1, PSMC6, FBXL7 |
| CL:16921 | Regulation of cytoskeletal remodeling and cell spreading by IPP complex components, and Integrin-linked protein kinase, pseudokinase domain | 4 | 6 | 1.57 | 0.0020 | LIMS2, PARVA, PARVB, LIMS1 |
| CL:22093 | Desmosome, and hemidesmosome | 6 | 27 | 1.09 | 0.0023 | PKP1, DST, PKP3, COL17A1, KAZN, PKP4 |
| CL:19397 | Microphthalmia with limb anomalies, and Interferon alpha/beta signaling | 5 | 16 | 1.24 | 0.0027 | ISG20, MX2, XAF1, OAS1, IFI35 |
| CL:16509 | Fibrillar collagen, C-terminal, and Procollagen C-endopeptidase enhancer, NTR domain | 5 | 19 | 1.17 | 0.0052 | ADAMTS2, COL27A1, TLL2, COL5A1, COL5A2 |
| CL:16871 | Integrin domain superfamily, and hematopoietic stem cell migration to bone marrow | 4 | 10 | 1.35 | 0.0077 | ITGAV, JAM3, ITGA11, FN1 |
| CL:21292 | Regulation of pathway-restricted SMAD protein phosphorylation, and Signaling by BMP | 8 | 72 | 0.79 | 0.0077 | TGFB3, FST, SMURF2, SMAD6, SMAD4, SMURF1, NEDD4L, SMAD1 |
| CL:26218 | Calcium-dependent cysteine-type endopeptidase activity | 4 | 10 | 1.35 | 0.0077 | CAPN15, CAPN13, CAPN12, CAST |
| CL:2878 | Ubiquitin-conjugating enzyme E2, and Ubiquitin fold domain | 5 | 22 | 1.1 | 0.0086 | UBE2K, UBE2Q2, UBA6, UBE2V1, UBE2H |
| CL:19399 | Mixed, incl. 2-5-oligoadenylate synthetase activity, and Nephropathia epidemica | 4 | 11 | 1.31 | 0.0094 | MX2, XAF1, OAS1, IFI35 |
| CL:21172 | Beta-catenin destruction complex, and Glycogen synthase kinase-3 binding | 4 | 12 | 1.27 | 0.0122 | APC, AXIN1, AXIN2, GSK3B |
| CL:16438 | FACIT collagen trimer, and procollagen-proline dioxygenase activity | 4 | 13 | 1.23 | 0.0154 | COL21A1, P4HA1, COL14A1, COL13A1 |
| CL:22159 | Claudin, conserved site, and Tight junction protein ZO | 5 | 26 | 1.03 | 0.0156 | CLDN16, CLDN20, CLDN6, CLDN8, CLDN2 |
| CL:16466 | Procollagen-lysine 5-dioxygenase activity, and Glycosyl transferase family 2 | 3 | 5 | 1.52 | 0.0225 | COLGALT1, PLOD2, COLGALT2 |
| CL:16665 | Lysyl oxidase, and Fibulin-5 | 3 | 5 | 1.52 | 0.0225 | LOX, ELN, LOXL2 |
| CL:21045 | Wnt signaling in kidney disease, and Negative regulation of TCF-dependent signaling by WNT ligand antagonists | 6 | 46 | 0.86 | 0.0227 | WNT3A, FZD1, LRP5, ROR1, ROR2, WNT5B |
| CL:2701 | Proteasome | 6 | 46 | 0.86 | 0.0227 | PSMB1, PSMD3, PSMA5, PSMD1, PSMC6, FBXL7 |
| CL:16610 | Mixed, incl. Domain of unknown function (DUF3377), and G2 nidogen domain and fibulin | 4 | 16 | 1.14 | 0.0272 | NID1, COL12A1, MMP17, HSPG2 |
| CL:2872 | Mixed, incl. ubiquitin conjugating enzyme activity, and Zinc finger, UBR-type | 7 | 69 | 0.75 | 0.0281 | UBE2K, UBE2Q2, UBE3C, UBA6, UBE2V1, UBE2H, KCMF1 |
| CL:16612 | Mixed, incl. G2 nidogen domain and fibulin, and collagen type XII trimer | 3 | 6 | 1.44 | 0.0302 | NID1, COL12A1, HSPG2 |
| CL:21174 | Beta-catenin destruction complex | 3 | 6 | 1.44 | 0.0302 | APC, AXIN2, GSK3B |
| CL:21216 | ZNRF-3, ectodomain, and R-spondin, Fu-CRD domain | 3 | 6 | 1.44 | 0.0302 | LGR5, RSPO2, LGR4 |
| CL:22096 | Desmosomal cadherin, and hemidesmosome | 4 | 17 | 1.12 | 0.0307 | PKP1, DST, PKP3, COL17A1 |
| CL:3946 | Histone demethylase activity (H3-K9 specific), and histone methyltransferase activity (H4-K20 specific) | 4 | 17 | 1.12 | 0.0307 | KDM4B, SETD7, KDM4C, KDM7A |
| CL:4615 | Mixed, incl. 7SK snRNA binding, and super elongation complex | 4 | 17 | 1.12 | 0.0307 | CCNT1, CCNT2, CDK9, DOT1L |
| CL:17127 | Mixed, incl. profilin binding, and Profilin | 5 | 33 | 0.93 | 0.0338 | VASP, IQGAP1, IQGAP2, ACTB, ACTN1 |
| CL:16813 | Mixed, incl. laminin-10 complex, and sprouting of injured axon | 3 | 7 | 1.38 | 0.0372 | LAMB1, LAMA5, LAMC1 |
| CL:3948 | Histone demethylase activity (H3-K9 specific) | 3 | 7 | 1.38 | 0.0372 | KDM4B, KDM4C, KDM7A |
| CL:2707 | Proteasome | 5 | 35 | 0.9 | 0.0391 | PSMB1, PSMD3, PSMA5, PSMD1, PSMC6 |
| CL:4633 | 7SK snRNA binding, and RNA 5-methyltransferase activity | 3 | 8 | 1.32 | 0.0473 | CCNT1, CCNT2, CDK9 |

**S7 Table. The recurring coding sequence variants in patients with KTCN.** The reference number of variation, description of variants according to HGVS nomenclature, gene symbol, consequence, and presence in the particular KTCN patient (10 OPT/KTCN, 13 OPT/KTCN, 18 OPT/KTCN, and 30 OPT/KTCN) are given.

| **Variation** | **Reference number** | **HGVS** | **Gene symbol** | **Consequence** | **KTCN10** | **KTCN13** | **KTCN18** | **KTCN30** |
| --- | --- | --- | --- | --- | --- | --- | --- | --- |
| 7:129126741CAGAGCTG>C | rs1563130264 | NC_000007.14:g.129126742_129126748del | AC011005.1 | frameshift_variant | FALSE | FALSE | TRUE | TRUE |
| GL000218.1:54175G>A | rs879192097 | ENST00000612565.1:c.40C>T | AL354822.1 | missense_variant | TRUE | FALSE | TRUE | TRUE |
| 19:54773534C>A | rs687485 | NC_000019.10:g.54773534C>A | KIR2DL1 | missense_variant | FALSE | TRUE | TRUE | FALSE |
| 19:54818581G>A | rs643861 | NC_000019.10:g.54818581G>A | KIR3DL1 | missense_variant | TRUE | TRUE | TRUE | FALSE |
| 19:54819832G>T | rs652641 | NC_000019.10:g.54819832G>T | KIR3DL1 | missense_variant | TRUE | TRUE | TRUE | FALSE |
| 11:1180138C>T | rs878913005 | NC_000011.10:g.1180138C>T | MUC5AC | missense_variant | TRUE | FALSE | FALSE | TRUE |
| 11:1183604G>A | rs878949904 | NC_000011.10:g.1183604G>A | MUC5AC | missense_variant | TRUE | FALSE | FALSE | TRUE |
| 11:1188446C>T | rs1269243287 | NC_000011.10:g.1188446C>T | MUC5AC | missense_variant | TRUE | FALSE | FALSE | TRUE |
| 15:35084921C>A | rs2257251 | NC_000015.10:g.35084921C>A | NANOGP8 | missense_variant | FALSE | FALSE | TRUE | TRUE |
| 1:149554508C>G | rs1227351091 | NC_000001.11:g.149554508C>G | NBPF19 | missense_variant | FALSE | FALSE | TRUE | TRUE |
| 7:26184630A>G | rs148235978 | NC_000007.14:g.26184630A>G | NFE2L3 | missense_variant | TRUE | TRUE | FALSE | FALSE |
| 14:22007907C>T | rs767573621 | NC_000014.9:g.22007907C>T | TRAV19 | stop_gained | TRUE | FALSE | TRUE | FALSE |
| 14:22007908A>C | rs752917826 | NC_000014.9:g.22007908A>C | TRAV19 | missense_variant | TRUE | FALSE | TRUE | FALSE |
| 7:142581165C>G | rs17229 | NC_000007.14:g.142581165C>G | TRBV12-5 | missense_variant | TRUE | TRUE | TRUE | TRUE |
| 3:150411052G>T | rs151080920 | NC_000003.12:g.150411052G>T | TSC22D2 | missense_variant | FALSE | TRUE | FALSE | TRUE |
| 10:13222107G>C | rs140771568 | NC_000010.11:g.13222107G>C | UCMA | missense_variant | FALSE | FALSE | TRUE | TRUE |
| 4:69391612A>G | rs28575804 | NC_000004.12:g.69391612A>G | UGT2B25P | splice_donor_variant | FALSE | FALSE | TRUE | TRUE |

**S8 Table. The results of pathway enrichment analysis of genes with the same coding sequence variants identified in more than one KTCN patient.** The KEGG database was chosen for definition of pathways.

| Pathway | Enrichment FDR | nGenes | Pathway Genes | Fold Enrichment | Genes |
| --- | --- | --- | --- | --- | --- |
| Graft-versus-host disease | 0.0007 | 2 | 42 | 120.6138 | KIR3DL1, KIR2DL1 |
| Antigen processing and presentation | 0.0012 | 2 | 78 | 64.94587 | KIR3DL1, KIR2DL1 |
| Natural killer cell mediated cytotoxicity | 0.0023 | 2 | 130 | 38.96752 | KIR3DL1, KIR2DL1 |
| IL-17 signaling pathway | 0.0542 | 1 | 93 | 27.23536 | MUC5AC |
| Signaling pathways regulating pluripotency of stem cells | 0.0661 | 1 | 143 | 17.71251 | NANOGP8 |
| Proteoglycans in cancer | 0.077 | 1 | 202 | 12.53905 | NANOGP8 |

**S9 Table. The results of pathway enrichment analysis of genes in which close proximity at least one sequence variant in regulatory element (RE) per KTCN patient was identified.** The KEGG database was chosen for definition of pathways.

| Pathway | Enrichment FDR | nGenes | Pathway Genes | Fold Enrichment | Genes |
| --- | --- | --- | --- | --- | --- |
| Focal adhesion | 0.00001 | 59 | 200 | 2.0331 | *ITGA2B, IGF1, FLT4, TNC, MAPK9, PIK3CB, LAMC2, RASGRF1, MYLK, RHOA, ACTN1, ACTB, PPP1R12B, ITGA8, GSK3B, ITGB5, LAMB4, PDGFB, ARHGAP5, LAMA1, PIK3R2, MYL10, RAPGEF1, MAPK8, VTN, CCND1, CCND3, VEGFA, ITGA4, TNR, RAP1A, PIK3CA, VASP, RAP1B, RAC2, FLNC, LAMA5, VAV3, PDGFRA, LAMC1, ITGA11, PAK6, ITGAV, ITGB7, PDGFC, PIK3R1, EGFR, DOCK1, COL6A3, PRKCB, PTK2, TLN2, JUN, PIP5K1C, PARVB, LAMA2, PARVA, TNXB, ACTN4* |
| Rap1 signaling pathway | 0.00001 | 60 | 210 | 1.9691 | *KRIT1, ITGA2B, RALA, IGF1, FLT4, PIK3CB, RHOA, PRKCZ, ACTB, APBB1IP, RAPGEF3, FYB1, RAPGEF4, PDGFB, PLCB4, PIK3R2, SIPA1L3, RAPGEF1, VEGFA, FGF1, RAP1A, ADCY7, PIK3CA, PARD6B, PLCG1, VASP, RAP1B, RAC2, DOCK4, KRAS, VAV3, PDGFRA, RAPGEF5, FGF5, RALB, PDGFC, PIK3R1, EGFR, ANGPT1, ENAH, ADCY8, TIAM1, KIT, MRAS, ADCY1, PRKCB, RGS14, FPR1, LPAR3, TLN2, ADCY5, CSF1R, PLCB1, EFNA5, MAPK11, MAGI2, EVL, SIPA1L1, RASSF5, MAGI1* |
| Phosphatidylinositol signaling system | 0.00021 | 32 | 97 | 2.2736 | *INPP4A, PIK3CB, DGKG, MTMR1, INPP5A, SYNJ2, MTMR2, MTMR3, CDS2, PLCB4, PIK3R2, INPP4B, PIK3CA, ITPR2, PLCG1, IPPK, DGKB, IMPA2, PIK3R1, PIP4K2A, ITPR1, IPMK, DGKI, SYNJ1, IP6K3, INPPL1, PRKCB, PLCB1, PIP5K1C, PLCG2, ITPK1, DGKD* |
| Leukocyte transendothelial migration | 0.00021 | 36 | 114 | 2.1764 | *CTNNA1, PIK3CB, RHOA, ACTN1, ACTB, RAPGEF3, RAPGEF4, ARHGAP5, PIK3R2, MYL10, CXCL12, ITK, CLDN16, ITGA4, RAP1A, PIK3CA, PLCG1, VASP, RAP1B, RAC2, VAV3, PIK3R1, CLDN8, CLDN2, JAM3, PRKCB, RHOH, PTK2, CLDN20, CLDN6, MAPK11, PLCG2, PECAM1, RASSF5, NCF4, ACTN4* |
| Pathways in cancer | 0.00021 | 115 | 530 | 1.4954 | *CREBBP, ITGA2B, RALA, IGF1, FLT4, CTNNA1, MAPK9, PIK3CB, LAMC2, GSTO2, RHOA, GNB5, PLD1, RUNX1T1, GSK3B, LAMB4, ESR1, MSH2, PDGFB, BDKRB1, STK4, PLCB4, JAG1, LAMA1, AXIN1, IL7, PIK3R2, CDK6, GLI3, CXCL12, MAPK8, CCND1, WNT5B, E2F3, CCND3, VEGFA, MSH3, FGF1, TFG, HDAC1, GADD45A, TGFB3, ADCY7, PIK3CA, NCOA3, PLCG1, RAC2, DLL4, LAMA5, RARA, KRAS, IL6ST, PDGFRA, ETS1, APC, LAMC1, ITGAV, FGF5, ESR2, PML, SMAD4, RXRG, MGST3, RALB, AGTR1, CAMK2D, PIK3R1, EGFR, CDKN2B, TCF7L2, GSTO1, EDNRA, WNT3A, ADCY8, FZD1, KIT, RUNX1, CTBP1, PTGER1, LRP5, IL15, IL3, ADCY1, PRKCB, STAT6, NKX3-1, BDKRB2, STAT3, AXIN2, PTK2, CXCL8, ALK, LPAR3, PLEKHG5, CYCS, ADCY5, STAT5B, GSTA3, CTBP2, TRAF6, JUN, CSF1R, PLCB1, TXNRD2, RXRA, GNG2, BCR, MITF, LAMA2, ARHGEF12, PLCG2, CSF2RA, IFNGR2, RASSF5, DLL1* |
| Axon guidance | 0.00035 | 49 | 181 | 1.8658 | *SEMA3G, PIK3CB, RHOA, PRKCZ, NCK2, PLXNA2, SEMA5B, GSK3B, ABLIM1, NRP1, NFATC2, PIK3R2, LIMK1, CXCL12, WNT5B, SEMA5A, EPHA4, PIK3CA, PARD6B, PLCG1, RAC2, KRAS, TRPC6, PAK6, SEMA6D, PPP3CA, RGS3, SEMA6C, CAMK2D, PIK3R1, RASA1, LRRC4C, SEMA3D, ENAH, ABLIM2, PTK2, CFL1, RHOD, ABLIM3, SLIT3, EFNA5, SLIT1, SEMA4D, SRGAP3, PLXNB2, ARHGEF12, SRGAP1, PLCG2, SRGAP2* |
| Inflammatory mediator regulation of TRP channels | 0.00056 | 31 | 98 | 2.1801 | *IGF1, PRKCH, MAPK9, PIK3CB, BDKRB1, PLCB4, TRPA1, PLA2G4C, PIK3R2, MAPK8, IL1R1, PLA2G4A, ADCY7, PIK3CA, ITPR2, PLCG1, TRPM8, CAMK2D, PIK3R1, ITPR1, ADCY8, F2RL1, ADCY1, PRKCB, BDKRB2, PRKCE, ADCY5, PLCB1, MAPK11, IL1RAP, PLCG2* |
| Phospholipase D signaling pathway | 0.00082 | 41 | 148 | 1.9092 | *RALA, CYTH3, AGPAT4, PIK3CB, DGKG, RHOA, PLD1, RAPGEF3, RAPGEF4, CYTH4, PDGFB, PLCB4, PLA2G4C, PIK3R2, CYTH1, GAB1, PLA2G4A, ADCY7, PIK3CA, PTGFR, PLCG1, KRAS, PDGFRA, DGKB, RALB, AGTR1, PDGFC, PIK3R1, EGFR, ADCY8, KIT, DGKI, MRAS, ADCY1, CXCL8, LPAR3, ADCY5, PLCB1, PIP5K1C, PLCG2, DGKD* |
| MAPK signaling pathway | 0.00203 | 67 | 294 | 1.5706 | *IGF1, FLT4, MAPK9, RASGRF1, MAP4K4, MEF2C, CACNA1S, MAP3K4, NLK, MAP3K20, MKNK2, PDGFB, TAB1, CACNA1I, STK4, STK3, PLA2G4C, HSPB1, MAPK8, CACNG1, VEGFA, FGF1, MAPKAPK3, IL1R1, RAP1A, PLA2G4A, GADD45A, TGFB3, NR4A1, RAP1B, RAC2, FLNC, DUSP9, NFATC1, KRAS, PDGFRA, TAOK3, ARRB1, DUSP5, FGF5, PPP3CA, CACNA1A, PDGFC, RASA1, EGFR, NTRK2, PTPRR, CACNA2D1, ANGPT1, KIT, MRAS, MAPKAPK2, PRKCB, HSPA6, TRAF6, JUN, ERBB4, CSF1R, EFNA5, MAPK11, NTF3, IL1RAP, CACNA1H, NF1, DUSP8, RPS6KA1, CACNA1C* |
| Fc gamma R-mediated phagocytosis | 0.00203 | 29 | 97 | 2.0605 | *GAB2, PIK3CB, PLD1, AMPH, PLA2G4C, PIK3R2, ACTR3C, LIMK1, PLA2G4A, PIK3CA, PLCG1, VASP, RAC2, ARPC1B, VAV3, BIN1, MYO10, PIK3R1, DOCK1, ASAP1, FCGR3B, INPPL1, PRKCB, PRKCE, CFL1, PIP5K1C, PLCG2, PTPRC, ASAP3* |
| Regulation of actin cytoskeleton | 0.00203 | 53 | 217 | 1.6833 | *ITGA2B, PIK3CB, CYFIP2, MYLK, RHOA, ACTN1, ACTB, PPP1R12B, ITGA8, ITGB5, PDGFB, MYH9, BDKRB1, ARHGEF7, MYH14, PIK3R2, MYL10, ACTR3C, LIMK1, CXCL12, FGF1, ITGA4, PIK3CA, RAC2, ARPC1B, KRAS, VAV3, PDGFRA, APC, ITGA11, PAK6, ITGAV, FGF5, ITGB7, IQGAP1, PDGFC, PIK3R1, IQGAP2, EGFR, DOCK1, PIP4K2A, ENAH, TIAM1, ITGAD, MRAS, BDKRB2, PTK2, CFL1, PIP5K1C, ARHGEF12, TMSB4X, CYFIP1, ACTN4* |
| MicroRNAs in cancer | 0.00203 | 42 | 161 | 1.7979 | *CREBBP, CD44, TNC, PIK3CB, RHOA, HDAC4, TP63, PDGFB, PIK3R2, CDK6, EZH2, CCND1, E2F3, VEGFA, FOXP1, GLS, TNR, HDAC1, PIK3CA, RECK, PLCG1, KRAS, PDGFRA, APC, KIF23, SLC7A1, TPM1, RPTOR, PIK3R1, EGFR, ZEB1, HMGA2, WNT3A, PRKCB, BMI1, STAT3, IRS1, PRKCE, EFNA5, PLCG2, TNXB, ABCC1* |
| Yersinia infection | 0.00228 | 37 | 137 | 1.8613 | *MAPK9, PIK3CB, RHOA, ACTB, FYB1, GSK3B, TAB1, NFATC2, ARHGEF7, PIK3R2, ACTR3C, LIMK1, MAPK8, ITGA4, WIPF1, PIK3CA, WIPF3, PLCG1, RAC2, ARPC1B, NFATC1, VAV3, GIT2, PIK3R1, DOCK1, CD8A, PTK2, CXCL8, TRAF6, RHOG, JUN, LCK, MAPK11, PIP5K1C, ARHGEF12, ARHGEF28, RPS6KA1* |
| AGE-RAGE signaling pathway in diabetic complications | 0.00312 | 29 | 100 | 1.9987 | *MAPK9, PIK3CB, PRKCZ, NOX4, PLCB4, PIK3R2, SERPINE1, MAPK8, CCND1, VEGFA, F3, TGFB3, PIK3CA, PLCG1, NFATC1, KRAS, SMAD4, AGTR1, PIK3R1, NOS3, PRKCB, STAT3, CXCL8, PRKCE, STAT5B, JUN, PLCB1, MAPK11, PLCG2* |
| VEGF signaling pathway | 0.00318 | 20 | 59 | 2.3362 | *PIK3CB, NFATC2, PLA2G4C, PIK3R2, HSPB1, VEGFA, MAPKAPK3, PLA2G4A, PIK3CA, PLCG1, RAC2, KRAS, PPP3CA, PIK3R1, MAPKAPK2, NOS3, PRKCB, PTK2, MAPK11, PLCG2* |
| Hippo signaling pathway | 0.00388 | 40 | 157 | 1.7559 | *WWTR1, CTNNA1, PRKCZ, ACTB, GSK3B, AXIN1, STK3, PPP2R1A, SERPINE1, BMPR1A, CCND1, WNT5B, CCND3, FGF1, WWC1, BMP8B, TGFB3, PARD6B, GDF5, DLG4, APC, YAP1, FRMD6, SMAD4, LIMD1, CRB2, TCF7L2, DLG2, SAV1, BMP6, FRMD1, WNT3A, FZD1, YWHAZ, AXIN2, YWHAG, SMAD1, TEAD1, CSNK1E, LLGL1* |
| Cushing syndrome | 0.00517 | 39 | 155 | 1.7341 | *CACNA1S, GSK3B, CACNA1I, PLCB4, AXIN1, CDK6, CCND1, WNT5B, E2F3, RAP1A, CREB1, ADCY7, CDKN2C, ITPR2, NR4A1, RAP1B, LDLR, APC, AGTR1, NCEH1, CAMK2D, EGFR, CDKN2B, TCF7L2, ITPR1, WNT3A, ADCY8, FZD1, CREB3L1, ADCY1, AXIN2, KCNK3, ADCY5, PLCB1, PBX1, WDR5, CACNA1H, CRHR1, CACNA1C* |
| Growth hormone synthesis, secretion and action | 0.00517 | 32 | 119 | 1.8533 | *CREBBP, IGF1, MAPK9, PIK3CB, CACNA1S, GSK3B, PLCB4, PIK3R2, MAPK8, CREB1, ADCY7, PIK3CA, ITPR2, PLCG1, KRAS, ADCY10, PIK3R1, ITPR1, ADCY8, SST, CREB3L1, ADCY1, PRKCB, STAT3, IRS1, PTK2, ADCY5, STAT5B, PLCB1, MAPK11, PLCG2, CACNA1C* |
| Shigellosis | 0.00517 | 56 | 246 | 1.5689 | *CYTH3, CD44, MAPK9, PIK3CB, RHOA, ACTN1, ACTB, GSK3B, CYTH4, TAB1, PLCB4, MAP1LC3A, PIK3R2, MYL10, ACTR3C, MAPK8, CYTH1, IL1R1, PIK3CA, SEPTIN7, ITPR2, PLCG1, ARPC1B, H3-3B, FNBP1L, SEPTIN11, RPTOR, PIK3R1, TNIP1, EGFR, DOCK1, ITPR1, CAST, HKDC1, TAB3, H3-3A, SEPTIN8, UBE2V2, PTK2, CXCL8, PRKCE, TLN2, CYCS, GLMN, TRAF6, JUN, UBE2N, SHARPIN, PLCB1, MAPK11, PLCG2, VDAC1, UBE2V1, MAP1LC3B2, ACTN4, H3C2* |
| Wnt signaling pathway | 0.00530 | 41 | 166 | 1.7022 | *CREBBP, MAPK9, RHOA, GSK3B, NLK, DAAM1, NFATC2, PLCB4, TBL1X, AXIN1, TLE4, MAPK8, CCND1, WNT5B, CCND3, RAC2, NFATC1, APC, PPP3CA, LGR5, SMAD4, CAMK2D, RSPO2, TCF7L2, WNT3A, FZD1, CTBP1, LRP5, VANGL2, PRICKLE2, PRKCB, AXIN2, CTNND2, CTBP2, TBL1XR1, JUN, PLCB1, ROR1, LGR4, CSNK1E, CSNK2A3* |
| Colorectal cancer | 0.00569 | 25 | 86 | 2.0035 | *RALA, MAPK9, PIK3CB, RHOA, GSK3B, MSH2, AXIN1, PIK3R2, MAPK8, CCND1, MSH3, GADD45A, TGFB3, PIK3CA, RAC2, KRAS, APC, SMAD4, RALB, PIK3R1, EGFR, TCF7L2, AXIN2, CYCS, JUN* |
| Inositol phosphate metabolism | 0.00696 | 22 | 73 | 2.0770 | *INPP4A, PIK3CB, MTMR1, INPP5A, SYNJ2, MTMR2, MTMR3, PLCB4, MINPP1, INPP4B, PIK3CA, PLCG1, IPPK, IMPA2, PIP4K2A, IPMK, SYNJ1, INPPL1, PLCB1, PIP5K1C, PLCG2, ITPK1* |
| GnRH secretion | 0.00708 | 20 | 64 | 2.1537 | *PIK3CB, CACNA1S, CACNA1I, PLCB4, KCNN4, PIK3R2, PIK3CA, ITPR2, KRAS, ARRB1, ESR2, KCNN3, PIK3R1, ITPR1, HCN1, PRKCB, KISS1, PLCB1, CACNA1H, CACNA1C* |
| TGF-beta signaling pathway | 0.00747 | 26 | 93 | 1.9268 | *CREBBP, DCN, LTBP1, RHOA, PPP2R1A, BMPR1A, SMURF2, ACVR1, BMP8B, TGFB3, INHBA, GDF5, E2F5, FST, ACVR1B, SMAD6, INHBE, SMAD4, CDKN2B, BMP6, FBN1, SMAD1, RGMB, THSD4, LEFTY1, SMURF1* |
| Cortisol synthesis and secretion | 0.00815 | 20 | 65 | 2.1206 | *CACNA1S, CACNA1I, PLCB4, CREB1, ADCY7, ITPR2, NR4A1, LDLR, AGTR1, NCEH1, ITPR1, ADCY8, CREB3L1, ADCY1, KCNK3, ADCY5, PLCB1, PBX1, CACNA1H, CACNA1C* |
| Non-small cell lung cancer | 0.01265 | 21 | 72 | 2.0101 | *PIK3CB, STK4, PIK3R2, CDK6, CCND1, E2F3, GADD45A, PIK3CA, PLCG1, KRAS, RXRG, PIK3R1, EGFR, PRKCB, STAT3, KIF5B, ALK, STAT5B, RXRA, PLCG2, RASSF5* |
| CGMP-PKG signaling pathway | 0.01534 | 39 | 166 | 1.6192 | *MYLK, RHOA, CNGB1, MEF2C, CACNA1S, NFATC2, PLCB4, CREB1, ADCY7, ITPR2, VASP, NFATC1, TRPC6, PPP3CA, AGTR1, KCNMB1, ITPR1, EDNRA, GUCY1A2, ADCY8, KCNMA1, ATP2B2, CREB3L1, ADORA1, ADCY1, NOS3, BDKRB2, IRS1, SLC25A6, ADRB2, PRKCE, PDE3A, ADCY5, PLCB1, PRKG1, VDAC1, ADORA3, GATA4, CACNA1C* |
| Sphingolipid signaling pathway | 0.01571 | 30 | 119 | 1.7375 | *MAPK9, PIK3CB, RHOA, PRKCZ, PLD1, PPP2R5C, PPP2R3C, PLCB4, PPP2R1A, PIK3R2, MAPK8, PIK3CA, RAC2, KRAS, CERS5, PIK3R1, CERS3, ADORA1, NOS3, PRKCB, BDKRB2, PRKCE, CERS6, SPTLC3, PLCB1, MAPK11, ASAH2, PPP2R3B, ABCC1, ADORA3* |
| Platelet activation | 0.01571 | 31 | 124 | 1.7230 | *ITGA2B, PIK3CB, MYLK, RHOA, PRKCZ, ACTB, APBB1IP, PLCB4, PLA2G4C, PIK3R2, RAP1A, PLA2G4A, ADCY7, PIK3CA, ITPR2, VASP, RAP1B, PIK3R1, ITPR1, GUCY1A2, ADCY8, ADCY1, NOS3, FGG, TLN2, ADCY5, PLCB1, MAPK11, PRKG1, ARHGEF12, PLCG2* |
| ErbB signaling pathway | 0.01664 | 23 | 84 | 1.8871 | *MAPK9, PIK3CB, NCK2, GSK3B, PIK3R2, MAPK8, GAB1, CBLB, PIK3CA, PLCG1, KRAS, PAK6, CAMK2D, PIK3R1, EGFR, NRG1, PRKCB, PTK2, STAT5B, JUN, ERBB4, NRG3, PLCG2* |
| Endocrine resistance | 0.01735 | 25 | 95 | 1.8137 | *IGF1, MAPK9, PIK3CB, ESR1, JAG1, PIK3R2, MAPK8, CCND1, E2F3, ADCY7, PIK3CA, CDKN2C, NCOA3, DLL4, KRAS, ESR2, PIK3R1, EGFR, ADCY8, ADCY1, PTK2, ADCY5, JUN, MAPK11, DLL1* |
| Tight junction | 0.01735 | 39 | 169 | 1.5904 | *NEDD4L, MAPK9, RHOA, PRKCZ, ACTN1, ACTB, EPB41L4B, MYH9, MYH14, PPP2R1A, ACTR3C, PRKAG2, MAPK8, CCND1, CLDN16, RAP1A, STK11, TJP2, PARD6B, VASP, ARPC1B, PATJ, DLG2, WHAMM, CLDN8, TIAM1, RUNX1, CLDN2, JAM3, TUBA1C, PRKCE, CLDN20, SYNPO, JUN, CLDN6, ACTN4, MAGI1, LLGL1, GATA4* |
| Proteoglycans in cancer | 0.01735 | 45 | 202 | 1.5353 | *DCN, IGF1, CD44, PIK3CB, GPC1, RHOA, ACTB, PPP1R12B, ITGB5, ESR1, PIK3R2, VTN, GAB1, CCND1, WNT5B, VEGFA, PIK3CA, ITPR2, PLCG1, FLNC, KRAS, VAV3, ITGAV, IQGAP1, HSPG2, CAMK2D, PIK3R1, EGFR, ITPR1, ANK3, WNT3A, TIAM1, FZD1, MRAS, FRS2, PRKCB, STAT3, PTK2, SDC2, HPSE2, ERBB4, MAPK11, ARHGEF12, PLCG2, TWIST2* |
| Adherens junction | 0.01855 | 20 | 71 | 1.9414 | *CREBBP, CTNNA1, RHOA, ACTN1, ACTB, NLK, SORBS1, SSX2IP, RAC2, LMO7, IQGAP1, SMAD4, EGFR, TCF7L2, PTPRJ, FER, PTPRM, NECTIN3, CSNK2A3, ACTN4* |
| Parathyroid hormone synthesis, secretion and action | 0.01855 | 27 | 106 | 1.7555 | *RHOA, PLD1, MEF2C, PTHLH, PLCB4, PDE4D, CREB1, ADCY7, ITPR2, RUNX2, KL, ARRB1, RXRG, CREB5, EGFR, ITPR1, ADCY8, CREB3L1, LRP5, ADCY1, PRKCB, ADCY5, PLCB1, PDE4B, RXRA, SLC34A3, MMP17* |
| Dilated cardiomyopathy | 0.01855 | 25 | 96 | 1.7948 | *ITGA2B, IGF1, ACTB, ITGA8, CACNA1S, ITGB5, LAMA1, CACNG1, ITGA4, TGFB3, ADCY7, MYBPC3, ITGA11, ITGAV, ITGB7, TPM1, CACNA2D1, ADCY8, LMNA, ADCY1, SGCD, ADCY5, LAMA2, DMD, CACNA1C* |
| Choline metabolism in cancer | 0.02447 | 25 | 98 | 1.7581 | *MAPK9, PIK3CB, DGKG, SLC44A1, PLD1, PDGFB, PLA2G4C, PIK3R2, MAPK8, PLA2G4A, PIK3CA, PLCG1, RAC2, KRAS, PDGFRA, DGKB, PDGFC, PIK3R1, EGFR, DGKI, PCYT1A, PRKCB, JUN, PIP5K1C, DGKD* |
| Ras signaling pathway | 0.02820 | 49 | 231 | 1.4619 | *RALA, IGF1, GAB2, FLT4, MAPK9, PIK3CB, RASGRF1, RHOA, GNB5, PLD1, PDGFB, STK4, PLA2G4C, PIK3R2, MAPK8, GAB1, VEGFA, FGF1, RAP1A, PLA2G4A, PIK3CA, PLCG1, RAP1B, RAC2, KRAS, PDGFRA, ETS1, RAPGEF5, PAK6, FGF5, RALB, PDGFC, PIK3R1, RASA1, EGFR, NTRK2, HTR7, ANGPT1, TIAM1, KIT, MRAS, PRKCB, CSF1R, EFNA5, NTF3, GNG2, NF1, PLCG2, RASSF5* |
| PI3K-Akt signaling pathway | 0.02831 | 70 | 354 | 1.3628 | *ITGA2B, IGF1, FLT4, TNC, PIK3CB, LAMC2, GNB5, ITGA8, PPP2R5C, GSK3B, ITGB5, LAMB4, PPP2R3C, OSM, PDGFB, LAMA1, IL7, PPP2R1A, PIK3R2, CDK6, VTN, CCND1, CCND3, VEGFA, PRLR, FGF1, ITGA4, TNR, STK11, CREB1, SGK1, PIK3CA, NR4A1, LAMA5, KRAS, PDGFRA, LAMC1, ITGA11, ITGAV, FGF5, ITGB7, RPTOR, PDGFC, OSMR, PIK3R1, CREB5, EGFR, NTRK2, ANGPT1, KIT, CREB3L1, COL6A3, IL3, NOS3, YWHAZ, IRS1, PTK2, YWHAG, LPAR3, ERBB4, CSF1R, EFNA5, NTF3, RXRA, GNG2, MAGI2, LAMA2, TNXB, PPP2R3B, MAGI1* |
| Renin secretion | 0.02831 | 19 | 69 | 1.8978 | *CACNA1S, PLCB4, CREB1, ITPR2, EDN3, PPP3CA, ADCYAP1, AGTR1, ITPR1, EDNRA, GUCY1A2, PDE1C, KCNMA1, ADORA1, ADRB2, PDE3A, PLCB1, AQP1, CACNA1C* |
| Fluid shear stress and atherosclerosis | 0.03214 | 32 | 138 | 1.5981 | *ITGA2B, MAPK9, PIK3CB, GPC1, GSTO2, RHOA, PRKCZ, ACTB, MEF2C, PDGFB, PIK3R2, MAPK8, BMPR1A, VEGFA, ACVR1, IL1R1, SUMO1, PIK3CA, RAC2, ASS1, ITGAV, MGST3, PIK3R1, GSTO1, NOS3, PTK2, SDC2, GSTA3, JUN, MAPK11, SUMO2, PECAM1* |
| Amoebiasis | 0.03728 | 25 | 102 | 1.6892 | *C8B, PIK3CB, LAMC2, ACTN1, ARG2, LAMB4, PLCB4, LAMA1, PIK3R2, HSPB1, IL1R1, TGFB3, PIK3CA, LAMA5, LAMC1, PIK3R1, C8A, ADCY1, PRKCB, PTK2, CXCL8, PLCB1, LAMA2, RAB7B, ACTN4* |
| Pancreatic cancer | 0.03728 | 20 | 76 | 1.8137 | *RALA, MAPK9, PIK3CB, PLD1, PIK3R2, CDK6, MAPK8, CCND1, E2F3, VEGFA, GADD45A, TGFB3, PIK3CA, RAC2, KRAS, SMAD4, RALB, PIK3R1, EGFR, STAT3* |
| Chemical carcinogenesis | 0.04206 | 42 | 197 | 1.4693 | *KPNA6, PIK3CB, GSTO2, CACNA1S, ESR1, JAG1, KLF5, PIK3R2, CCND1, CCND3, VEGFA, CREB1, ADCY7, PIK3CA, DLL4, KRAS, ARRB1, PAQR5, FGF5, ESR2, CACNA1A, RXRG, MGST3, PIK3R1, CREB5, EGFR, GSTO1, ADCY8, CREB3L1, ADCY1, PRKCB, STAT3, ADRB2, ADCY5, STAT5B, GSTA3, JUN, RXRA, KPNA4, DLL1, RPS6KA1, CACNA1C* |
| Aldosterone synthesis and secretion | 0.04243 | 24 | 98 | 1.6878 | *CACNA1S, CACNA1I, PLCB4, CREB1, ADCY7, ITPR2, ATF1, NR4A1, LDLR, AGTR1, CAMK2D, ITPR1, ADCY8, ATP2B2, CREB3L1, ADCY1, PRKCB, PRKCE, KCNK3, ADCY5, CYP11B2, PLCB1, CACNA1H, CACNA1C* |
| FoxO signaling pathway | 0.04521 | 30 | 131 | 1.5783 | *CREBBP, IGF1, MAPK9, PIK3CB, NLK, STK4, HOMER2, PIK3R2, PRKAG2, MAPK8, CCND1, GADD45A, STK11, SGK1, TGFB3, PIK3CA, CDKN2D, KRAS, SMAD4, SETD7, PIK3R1, EGFR, FBXO25, CDKN2B, FBXO32, STAT3, IRS1, MAPK11, USP7, CSNK1E* |
| Cell cycle | 0.04673 | 29 | 126 | 1.5862 | *MAD1L1, CDC27, CREBBP, CDC14B, GSK3B, ANAPC5, MCM5, MCM4, CDK6, CCND1, E2F3, CCND3, HDAC1, GADD45A, STAG1, TGFB3, CDKN2C, ANAPC13, CDKN2D, E2F5, SMAD4, CDKN2B, ANAPC1, BUB1B, PTTG1, YWHAZ, YWHAG, CDK1, PRKDC* |
| Calcium signaling pathway | 0.04866 | 49 | 240 | 1.4071 | *FLT4, MYLK, CACNA1S, P2RX7, PDGFB, CACNA1I, BDKRB1, PLCB4, TNNC2, VEGFA, FGF1, TACR1, CASQ2, ADCY7, PTGFR, ITPR2, PLCG1, PDGFRA, FGF5, PPP3CA, CACNA1A, AGTR1, CAMK2D, PDGFC, EGFR, NTRK2, HTR7, ITPR1, EDNRA, PDE1C, ADCY8, MCU, ATP2B2, PTGER1, TPCN2, ADCY1, NOS3, PRKCB, BDKRB2, SLC25A6, ADRB2, ERBB4, PLCB1, TPCN1, CACNA1H, PLCG2, ASPH, VDAC1, CACNA1C* |
| Endocytosis | 0.04892 | 51 | 252 | 1.3948 | *CYTH3, VTA1, RABEP1, NEDD4L, PSD, RHOA, PRKCZ, EPN2, PLD1, AMPH, SNX5, WASHC2A, CYTH4, CHMP4B, ACTR3C, CYTH1, SMURF2, CBLB, WIPF1, WIPF3, PARD6B, PSD4, EPS15L1, SNX6, LDLR, ARPC1B, VPS4A, PDGFRA, BIN1, ARRB1, GIT2, PML, MVB12A, IQSEC1, EGFR, SH3GLB2, ASAP1, PSD3, AGAP1, RAB31, KIF5B, HSPA6, TRAF6, VPS37D, IST1, IZUMO1R, PIP5K1C, RNF103-CHMP3, IQSEC3, ASAP3, SMURF1* |
| Hypertrophic cardiomyopathy | 0.05426 | 22 | 90 | 1.6847 | *ITGA2B, IGF1, ACTB, ITGA8, CACNA1S, ITGB5, LAMA1, PRKAG2, CACNG1, ITGA4, TGFB3, MYBPC3, ITGA11, ITGAV, ITGB7, TPM1, CACNA2D1, LMNA, SGCD, LAMA2, DMD, CACNA1C* |
| CAMP signaling pathway | 0.05524 | 45 | 219 | 1.4161 | *CREBBP, MAPK9, PIK3CB, RHOA, CNGB1, PLD1, RAPGEF3, CACNA1S, RAPGEF4, PIK3R2, GLI3, MAPK8, PDE4D, RAP1A, CREB1, ADCY7, PIK3CA, EDN3, ABCC4, RAP1B, RAC2, NFATC1, VAV3, HTR1B, GABBR2, ADCYAP1, ADCY10, CAMK2D, PIK3R1, EDNRA, GRIA1, ADCY8, TIAM1, SST, ATP2B2, CREB3L1, ADORA1, ADCY1, ADRB2, PDE3A, ADCY5, JUN, PDE4B, CRHR1, CACNA1C* |

**S10 Table. The results of pathway enrichment analysis (KEGG database) (A) and gene ontology (GO) term enrichment analysis for 'Cellular Component' category (B).** Analysis embraced genes with variants identified in coding sequence and genes in which close proximity at least one sequence variant in regulatory element (RE) per KTCN patient was identified.

**A)**

| Pathway | Enrichment FDR | nGenes | Pathway Genes | Fold Enrichment | Genes |
| --- | --- | --- | --- | --- | --- |
| Focal adhesion | 0.0034 | 25 | 200 | 2.6166 | *ITGA2B, RASGRF1, MYLK, ACTN1, PPP1R12B, ITGA8, ITGB5, LAMB1, MAPK8, CCND1, ITGA4, VASP, RAP1B, RAC2, FLNC, LAMA5, VAV3, EGFR, DOCK1, COL6A3, PRKCB, PTK2, PIP5K1C, LAMA2, TNXB* |
| Regulation of actin cytoskeleton | 0.0069 | 25 | 217 | 2.4116 | *ITGA2B, INSRR, MYLK, ACTN1, PPP1R12B, ITGA8, ITGB5, ARHGEF7, FGF1, ITGA4, RAC2, VAV3, FGF5, IQGAP2, EGFR, DOCK1, PIP4K2A, ENAH, TIAM1, MRAS, BDKRB2, PTK2, PIP5K1C, TMSB4X, CYFIP1* |
| Rap1 signaling pathway | 0.0197 | 23 | 210 | 2.2927 | *ITGA2B, RALA, FYB1, FGF1, VASP, RAP1B, RAC2, DOCK4, VAV3, FGF5, EGFR, ENAH, TIAM1, MRAS, ADCY1, PRKCB, RGS14, ADCY5, EFNA5, MAPK11, SIPA1L1, RASSF5, MAGI1* |
| Leukocyte transendothelial migration | 0.0218 | 15 | 114 | 2.7543 | *ACTN1, ICAM1, ITGA4, VASP, RAP1B, RAC2, VAV3, PRKCB, RHOH, PTK2, CLDN20, CLDN6, MAPK11, PECAM1, RASSF5* |
| Glutamatergic synapse | 0.0218 | 15 | 114 | 2.7543 | *SLC1A3, HOMER2, PLA2G4C, SLC1A2, SLC38A1, GLS, PLA2G4A, DLG4, CACNA1A, GRIA1, GRIK2, ADCY1, PRKCB, ADCY5, GNG2* |
| Axon guidance | 0.0224 | 20 | 181 | 2.3130 | *NCK2, SEMA3C, ABLIM1, NRP1, NFATC2, EPHA4, RAC2, SEMA6D, RGS3, SLIT2, CAMK2D, LRRC4C, ENAH, ABLIM2, PTK2, ABLIM3, SLIT3, EFNA5, SRGAP3, SRGAP2* |
| Phosphatidylinositol signaling system | 0.0316 | 13 | 97 | 2.8054 | *DGKG, MTMR1, SYNJ2, CDS2, INPP4B, IPPK, PIP4K2A, IPMK, DGKI, SYNJ1, INPPL1, PRKCB, PIP5K1C* |
| CGMP-PKG signaling pathway | 0.0397 | 18 | 166 | 2.2698 | *MYLK, CNGB1, MEF2C, NFATC2, VASP, NFATC1, EDNRA, KCNMA1, ATP2B2, ADORA1, ADCY1, BDKRB2, IRS1, SLC25A6, ADRB2, ADCY5, PRKG1, VDAC1* |
| MAPK signaling pathway | 0.0617 | 26 | 294 | 1.8512 | *MAPK8IP2, RASGRF1, MEF2C, MAP3K20, TAB1, CACNA1I, PLA2G4C, MAPK8, FGF1, IL1R1, PLA2G4A, RAP1B, RAC2, FLNC, DUSP9, NFATC1, FGF5, CACNA1A, EGFR, MRAS, MAPKAPK2, PRKCB, HSPA6, EFNA5, MAPK11, NF1* |
| Adherens junction | 0.0617 | 10 | 71 | 2.9483 | *ACTN1, SORBS1, RAC2, NECTIN2, LMO7, SMAD4, EGFR, PTPRJ, PTPRM, CSNK2A3* |
| Fc gamma R-mediated phagocytosis | 0.0640 | 12 | 97 | 2.5896 | *PLA2G4C, PLA2G4A, VASP, RAC2, VAV3, MYO10, DOCK1, FCGR3B, INPPL1, PRKCB, PIP5K1C, PTPRC* |
| Fluid shear stress and atherosclerosis | 0.0657 | 15 | 138 | 2.2753 | *ITGA2B, GSTO2, MEF2C, ICAM1, MAPK8, ACVR1, IL1R1, RAC2, GSTO1, PTK2, SDC2, GSTA3, MAPK11, SUMO2, PECAM1* |
| Inositol phosphate metabolism | 0.1143 | 9 | 73 | 2.5808 | *MTMR1, SYNJ2, INPP4B, IPPK, PIP4K2A, IPMK, SYNJ1, INPPL1, PIP5K1C* |
| CAMP signaling pathway | 0.1143 | 19 | 219 | 1.8161 | *CNGB1, MAPK8, RAP1B, RAC2, NFATC1, VAV3, HTR1B, GABBR2, CAMK2D, EDNRA, GRIA1, TIAM1, SST, ATP2B2, ADORA1, ADCY1, ADRB2, ADCY5, PDE4B* |
| Necroptosis | 0.1143 | 15 | 159 | 1.9748 | *CFLAR, EIF2AK2, TYK2, PLA2G4C, MAPK8, PLA2G4A, ZBP1, CAMK2D, SPATA2L, MLKL, STAT3, SLC25A6, STAT5B, SHARPIN, VDAC1* |
| VEGF signaling pathway | 0.1143 | 8 | 59 | 2.8384 | *NFATC2, PLA2G4C, PLA2G4A, RAC2, MAPKAPK2, PRKCB, PTK2, MAPK11* |
| ECM-receptor interaction | 0.1143 | 10 | 88 | 2.3787 | *ITGA2B, CD44, ITGA8, ITGB5, LAMB1, ITGA4, LAMA5, COL6A3, LAMA2, TNXB* |
| Inflammatory mediator regulation of TRP channels | 0.1143 | 11 | 98 | 2.3496 | *PRKCH, PLA2G4C, MAPK8, IL1R1, PLA2G4A, CAMK2D, ADCY1, PRKCB, BDKRB2, ADCY5, MAPK11* |
| Prolactin signaling pathway | 0.1143 | 9 | 70 | 2.6914 | *MAPK8, CCND1, PRLR, IRF1, ESR2, STAT3, SOCS6, STAT5B, MAPK11* |
| Oxytocin signaling pathway | 0.1143 | 15 | 154 | 2.0389 | *MYLK, PPP1R12B, MEF2C, NFATC2, PLA2G4C, PRKAG2, CCND1, PLA2G4A, NFATC1, CAMK2D, EGFR, ADCY1, PRKCB, ADCY5, KCNJ12* |
| AGE-RAGE signaling pathway in diabetic complications | 0.1143 | 11 | 100 | 2.3026 | *NOX4, ICAM1, SERPINE1, MAPK8, CCND1, NFATC1, SMAD4, PRKCB, STAT3, STAT5B, MAPK11* |
| Yersinia infection | 0.1143 | 14 | 137 | 2.1391 | *FYB1, TAB1, NFATC2, ARHGEF7, MAPK8, ITGA4, RAC2, NFATC1, VAV3, DOCK1, PTK2, MAPK11, PIP5K1C, ARHGEF28* |
| Amoebiasis | 0.1143 | 11 | 102 | 2.2575 | *C8B, ACTN1, LAMB1, IL1R1, LAMA5, C8A, ADCY1, PRKCB, PTK2, LAMA2, RAB7B* |
| Proteoglycans in cancer | 0.1143 | 18 | 202 | 1.8653 | *CD44, PPP1R12B, ITGB5, CCND1, FLNC, VAV3, CAMK2D, EGFR, TIAM1, MRAS, FRS2, PRKCB, STAT3, PTK2, SDC2, HPSE2, MAPK11, NANOGP8* |
| Chemical carcinogenesis | 0.1143 | 18 | 197 | 1.9127 | *GSTO2, KLF5, CCND1, FGF5, ESR2, CACNA1A, RXRG, EGFR, GSTO1, ADCY1, PRKCB, STAT3, ADRB2, ADCY5, STAT5B, GSTA3, RXRA, UGT2B11* |
| Non-small cell lung cancer | 0.1143 | 9 | 72 | 2.6166 | CCND1 RXRG EGFR PRKCB STAT3 KIF5B STAT5B RXRA RASSF5 |
| Ovarian steroidogenesis | 0.1195 | 7 | 51 | 2.8732 | PLA2G4C PLA2G4A CYP19A1 BMP6 ADCY1 ADCY5 AKR1C3 |
| Cell adhesion molecules | 0.1362 | 14 | 149 | 1.9669 | ITGA8 ICAM1 GLG1 NRCAM ITGA4 NECTIN2 LRRC4C SDC2 CLDN20 PTPRM CLDN6 PDCD1LG2 PECAM1 PTPRC |
| Th17 cell differentiation | 0.1536 | 11 | 108 | 2.1321 | NFATC2 TYK2 MAPK8 IL1R1 NFATC1 SMAD4 RXRG STAT3 STAT5B MAPK11 RXRA |
| Choline metabolism in cancer | 0.1950 | 10 | 98 | 2.1360 | DGKG PLA2G4C MAPK8 PLA2G4A RAC2 EGFR DGKI PCYT1A PRKCB PIP5K1C |

**B)**

| Pathway | Enrichment FDR | nGenes | Pathway Genes | Fold Enrichment | Genes |
| --- | --- | --- | --- | --- | --- |
| Anchoring junction | <0.0001 | 232 | 907 | 1.5898 | *KRIT1, ITGA2B, RALA, CYTH3, MRC2, ABCC2, CD44, ARHGAP31, TNC, CTNNA1, LIMA1, SYNE2, GDI2, TNK2, COL17A1, RHOA, PRKCZ, FGFR3, MAP4K4, CDH19, ACTN1, LIMS2, PANX2, FERMT2, HACD3, ACTB, APBB1IP, ITGA8, TNS1, CEACAM1, EPB41L2, PKP1, ITGB5, GRHL2, FAT1, CD59, AKR1B1, FAT2, NOX4, CASS4, ICAM1, EPB41L4B, SORBS1, NRP1, MISP, MYH9, FERMT1, MAPRE1, JAG1, VAPA, LAMA1, ARHGEF7, CORO2B, CSK, NDRG1, RPS16, SIPA1L3, HSPB1, LIMK1, DNMBP, CYTH1, GAB1, HSPA8, LPXN, CCND1, CD81, PPFIBP1, CORO1C, LIN7A, TNS2, VEGFA, ERBIN, ITK, CDH6, DBN1, CLDN16, FRMD4B, ITGA4, IL1RL1, STRN, EPHA4, SSX2IP, TJP2, SORBS3, GJA8, PIK3CA, PARD6B, PLCG1, VASP, WNK4, RAP1B, PTPN12, RAC2, FLNC, ARHGAP22, PALLD, FGF13, NECTIN2, ARPC1B, PDLIM4, PATJ, MPRIP, KRAS, APC, ANXA1, LMO7, TNS3, NIBAN2, TLN1, SDCBP, TRPC6, KIF23, ITGA11, ITGAV, ARHGAP24, SHROOM3, PPP3CA, GIT2, ITGB7, IQGAP1, GRB7, APP, HSPG2, RHOB, PKP4, SCN1A, LIMD1, PHLDB2, LPP, ILDR1, USP53, PIK3R1, ARHGAP26, G3BP1, EGFR, SHROOM2, MTDH, ADD3, LIN7C, PTPRJ, DIXDC1, ANK3, FRMD4A, ADAM17, SAV1, DST, OBSCN, ENAH, PDLIM3, CLDN8, TIAM1, FZD1, KIT, SPTBN4, FGFR4, NEXN, VANGL2, XIRP2, PDLIM5, ANXA5, SHROOM1, GJB7, RPS14, DLC1, YWHAZ, CLDN2, HEPACAM, JAM3, FRS2, CLMP, PLEKHA7, MAPRE2, EVPL, IRF2, STXBP6, PTK2, LIMS1, CTNND2, YWHAG, CLDN20, PLEKHG5, FRMD5, TLN2, SYNPO, IL16, PDZD3, SYNPO2, CFL1, RPL38, PTPRM, PEAK1, HEG1, CSRP2, RHOG, NECTIN3, CALR, KIRREL1, PKP3, CLDN6, UBOX5, FLRT2, ATP6V0C, PIP5K1C, MAGI2, AMTN, NHS, FOCAD, PARVB, KAZN, EVL, AFAP1, MME, ANXA6, SVIL, COL13A1, PARVA, NRAP, DMD, TGM2, STARD10, AJM1, SCARF2, FMN1, SPECC1L-ADORA2A, PECAM1, PTPRC, PTPRK, DLL1, ACTN4, ASAP3, MAGI1, FLII* |
| Actin cytoskeleton | <0.0001 | 152 | 542 | 1.7430 | *ANLN, MYO16, CTNNA1, ARHGAP6, LIMA1, TTC17, CROCC, LIMCH1, MYLK, MYO9A, PRKCZ, HDAC4, MYO3B, ACTN1, MARK2, LLGL2, FERMT2, ACTB, AMPH, NEBL, RAPGEF3, CARMIL1, EPB41L2, FYB1, NOX4, SORBS1, ABLIM1, MYO9B, MISP, MYH9, DAAM1, TNNC2, INTS6, CORO2B, MYH14, SIPA1L3, COBL, ACTR3C, ARHGAP21, SH3PXD2A, LPXN, CORO1C, CLIC5, DBN1, SPTBN1, MLPH, WIPF1, ACTL8, GBP1, MED28, ALG2, MYOT, SEPTIN7, WIPF3, CALD1, VASP, MACF1, VIL1, HIP1, PTPN12, RAC2, MYO1B, PALLD, ARPC1B, HIP1R, PDLIM4, RARA, LANCL2, MPRIP, MYO18B, FHOD3, ANXA1, CCDC102A, BIN1, TMEM63B, SEPTIN11, SHROOM3, TPM1, IQGAP1, CTTNBP2NL, HAX1, MYH15, LPP, MYO10, IQGAP2, DCDC2, SHROOM2, DENND2A, ALDOA, FER, ADAM17, PLEKHH2, UTRN, ASAP1, PDLIM3, TTN, ADCY8, AUTS2, PPP1R9A, LAD1, KALRN, SPTBN4, SCNN1D, NEXN, XIRP2, PDLIM5, SPTA1, ABLIM2, SHROOM1, DLC1, PKNOX2, JAM3, PRKCB, MYO1A, MYO5B, MYL1, PTK2, MYO7B, MTSS1, TLN2, SYNPO, MYOZ2, SYNPO2, CFL1, ABLIM3, PEAK1, SPTBN2, SH3PXD2B, RFLNA, ARSJ, ROR1, FSCN2, ESPN, PARVB, AFAP1, SVIL, MYO5A, SIPA1L1, MYH6, PARVA, DMD, ITSN1, STK38L, NEURL1B, FMN1, MYZAP, MYO15B, SRCIN1, ACACA, ACTN4, LLGL1, FLII* |
| External encapsulating structure | <0.0001 | 158 | 601 | 1.6340 | *DCN, TNC, USH2A, ADAMTS6, LTBP1, ELN, COL23A1, LAMC2, GPC1, COL17A1, TGFBR3, GPC4, FBLN1, IMPG2, ICAM1, P3H2, GLG1, LAMB4, LAMB1, PDGFB, CHADL, PAPLN, LAMA1, MXRA5, IL7, FGL1, APLP1, PTPRZ1, SERPINE1, AEBP1, ASPN, ATRNL1, CXCL12, VTN, CTSC, VWF, WNT5B, COL12A1, VEGFA, CCN6, ERBIN, LOX, FGF1, HRG, EFEMP1, FN1, TNR, NID1, P3H1, F3, MUC5B, TGFB3, TGFBI, A1BG, PLG, ANXA11, PI3, COL21A1, OMD, LRRC17, APOE, PXDN, COL5A1, LAMA5, FIBCD1, EMILIN2, ENAM, LOXL2, ADAMDEC1, ANXA1, ADAM19, LAMC1, SERPINE2, ADAMTS7, EMILIN1, MMRN1, FBN2, INHBE, PTPRQ, PCSK6, HAPLN3, MFGE8, ADAMTS10, HSPG2, ECM1, FLG, TMEFF2, LRIG1, COL8A1, HMCN2, HSD17B12, SERPING1, NCAM1, FREM2, DST, SPARCL1, ABI3BP, ANGPT1, ACAN, COLEC12, LAD1, MATN1, COL6A3, FBLN2, ADAMTS9, CPA3, ANXA5, EGFLAM, BMPER, TNFRSF11B, UCMA, HTRA1, FBN1, NAV2, ELFN2, ANGPTL4, LGALS9, SDC2, MUC17, FGG, LRRC15, EFEMP2, HPSE2, HPSE, MMRN2, PODN, LRRN1, CLEC14A, ADAMTSL1, CPN2, CALR, MXRA7, CCBE1, FLRT2, AMTN, THSD4, COL14A1, OTOG, ZP3, COL25A1, LAMA2, COL27A1, ANXA6, COL13A1, MFAP5, ADAMTSL2, MMP17, TGM2, COL5A2, VIT, GPC2, COL28A1, ELFN1, RBP3, GDF10, POMZP3, MUC5AC, ADAMTS2* |
| Extracellular matrix | <0.0001 | 158 | 600 | 1.6367 | *DCN, TNC, USH2A, ADAMTS6, LTBP1, ELN, COL23A1, LAMC2, GPC1, COL17A1, TGFBR3, GPC4, FBLN1, IMPG2, ICAM1, P3H2, GLG1, LAMB4, LAMB1, PDGFB, CHADL, PAPLN, LAMA1, MXRA5, IL7, FGL1, APLP1, PTPRZ1, SERPINE1, AEBP1, ASPN, ATRNL1, CXCL12, VTN, CTSC, VWF, WNT5B, COL12A1, VEGFA, CCN6, ERBIN, LOX, FGF1, HRG, EFEMP1, FN1, TNR, NID1, P3H1, F3, MUC5B, TGFB3, TGFBI, A1BG, PLG, ANXA11, PI3, COL21A1, OMD, LRRC17, APOE, PXDN, COL5A1, LAMA5, FIBCD1, EMILIN2, ENAM, LOXL2, ADAMDEC1, ANXA1, ADAM19, LAMC1, SERPINE2, ADAMTS7, EMILIN1, MMRN1, FBN2, INHBE, PTPRQ, PCSK6, HAPLN3, MFGE8, ADAMTS10, HSPG2, ECM1, FLG, TMEFF2, LRIG1, COL8A1, HMCN2, HSD17B12, SERPING1, NCAM1, FREM2, DST, SPARCL1, ABI3BP, ANGPT1, ACAN, COLEC12, LAD1, MATN1, COL6A3, FBLN2, ADAMTS9, CPA3, ANXA5, EGFLAM, BMPER, TNFRSF11B, UCMA, HTRA1, FBN1, NAV2, ELFN2, ANGPTL4, LGALS9, SDC2, MUC17, FGG, LRRC15, EFEMP2, HPSE2, HPSE, MMRN2, PODN, LRRN1, CLEC14A, ADAMTSL1, CPN2, CALR, MXRA7, CCBE1, FLRT2, AMTN, THSD4, COL14A1, OTOG, ZP3, COL25A1, LAMA2, COL27A1, ANXA6, COL13A1, MFAP5, ADAMTSL2, MMP17, TGM2, COL5A2, VIT, GPC2, COL28A1, ELFN1, RBP3, GDF10, POMZP3, MUC5AC, ADAMTS2* |
| Cell leading edge | <0.0001 | 124 | 445 | 1.7319 | *ALS2, CYTH3, RUFY3, CD44, ARHGAP31, USH2A, CTNNA1, LIMA1, SYNE2, PSD, MYLK, RHOA, PRKCZ, ACTN1, FERMT2, ACTB, APBB1IP, ITGA8, AMPH, RAPGEF3, CARMIL1, FAT1, SNX5, ABLIM1, MYH9, FERMT1, BMX, ARHGEF7, PIEZO1, CDK6, COBL, LIMK1, DOCK8, KANK1, MPP2, SLC1A2, CORO1C, RIPOR2, WWC1, DBN1, FRMD4B, SPTBN1, WIPF1, AAK1, WLS, EXOC8, SSX2IP, PIK3CA, PLCG1, VAMP7, PACSIN1, PSD4, VASP, MACF1, VIL1, RAC2, PALLD, CLIP1, HIP1R, EPS8L1, PDLIM4, INPP5K, APC, CAPRIN1, TLN1, ITGAV, FGD4, TPM1, IQGAP1, BCAS3, DPP9, APP, CTTNBP2NL, HAX1, PHLDB2, MYO10, IQGAP2, RASA1, ARHGAP18, TIAM2, EGFR, PTPRJ, FER, EPS8, ADAM17, DST, PLEKHH2, ENAH, SH3RF1, FGD5, GRIA1, PSD3, TIAM1, PPP1R9A, ADORA1, APBB2, ADGRV1, DLC1, INPPL1, KCNC2, ANTXR1, LDB2, MTSS1, MCC, PLEKHG5, TLN2, CFL1, ABLIM3, PTPRM, PIP5K1C, NHS, PARVB, EVL, MYO5A, PARVA, PLCG2, DMD, SNX2, ITSN1, DDX3X, ARPIN, SRGAP2, PTPRK, ASAP3* |
| Collagen-containing extracellular matrix | <0.0001 | 124 | 446 | 1.7280 | *DCN, TNC, USH2A, LTBP1, ELN, COL23A1, LAMC2, GPC1, COL17A1, GPC4, FBLN1, IMPG2, ICAM1, P3H2, LAMB4, LAMB1, PDGFB, CHADL, LAMA1, MXRA5, IL7, FGL1, APLP1, PTPRZ1, SERPINE1, AEBP1, ASPN, ATRNL1, CXCL12, VTN, CTSC, VWF, WNT5B, COL12A1, ERBIN, HRG, EFEMP1, FN1, TNR, NID1, P3H1, F3, TGFB3, TGFBI, A1BG, PLG, ANXA11, COL21A1, OMD, APOE, PXDN, COL5A1, LAMA5, FIBCD1, EMILIN2, LOXL2, ADAMDEC1, ANXA1, ADAM19, LAMC1, SERPINE2, EMILIN1, MMRN1, FBN2, INHBE, PTPRQ, PCSK6, MFGE8, ADAMTS10, HSPG2, ECM1, FLG, TMEFF2, COL8A1, HMCN2, SERPING1, NCAM1, FREM2, DST, SPARCL1, ABI3BP, ANGPT1, ACAN, LAD1, MATN1, COL6A3, FBLN2, ADAMTS9, CPA3, ANXA5, EGFLAM, HTRA1, FBN1, NAV2, ANGPTL4, LGALS9, SDC2, MUC17, FGG, LRRC15, EFEMP2, MMRN2, PODN, CLEC14A, CALR, MXRA7, AMTN, THSD4, COL14A1, ZP3, COL25A1, LAMA2, COL27A1, ANXA6, COL13A1, MFAP5, TGM2, COL5A2, VIT, GPC2, COL28A1, RBP3, GDF10, ADAMTS2* |
| Cell-substrate junction | <0.0001 | 127 | 478 | 1.6513 | *ITGA2B, RALA, MRC2, CD44, ARHGAP31, TNC, CTNNA1, LIMA1, SYNE2, GDI2, COL17A1, RHOA, FGFR3, MAP4K4, ACTN1, LIMS2, FERMT2, HACD3, ACTB, APBB1IP, ITGA8, TNS1, EPB41L2, ITGB5, FAT1, CD59, NOX4, CASS4, ICAM1, SORBS1, NRP1, MISP, MYH9, FERMT1, MAPRE1, ARHGEF7, CORO2B, RPS16, HSPB1, LIMK1, HSPA8, LPXN, CD81, PPFIBP1, CORO1C, TNS2, ERBIN, ITGA4, IL1RL1, SORBS3, VASP, PTPN12, RAC2, FLNC, ARHGAP22, PALLD, NECTIN2, ARPC1B, MPRIP, KRAS, ANXA1, LMO7, TNS3, TLN1, SDCBP, KIF23, ITGA11, ITGAV, ARHGAP24, GIT2, ITGB7, IQGAP1, GRB7, HSPG2, RHOB, LIMD1, PHLDB2, LPP, ARHGAP26, G3BP1, EGFR, DIXDC1, ADAM17, DST, ENAH, FZD1, NEXN, XIRP2, ANXA5, RPS14, DLC1, YWHAZ, MAPRE2, IRF2, PTK2, LIMS1, YWHAG, TLN2, IL16, SYNPO2, CFL1, RPL38, PEAK1, CSRP2, RHOG, CALR, UBOX5, FLRT2, ATP6V0C, PIP5K1C, NHS, FOCAD, PARVB, EVL, AFAP1, MME, ANXA6, SVIL, PARVA, NRAP, DMD, TGM2, SCARF2, PTPRC, ACTN4, ASAP3, FLII* |
| Cell cortex | <0.0001 | 94 | 332 | 1.7597 | *RHOBTB2, ANLN, LAMC2, RHOA, PRKCZ, LLGL2, FERMT2, FRY, ACTB, RAPGEF3, EPB41L2, RIMS1, ERC1, OSBPL8, MYO9B, MISP, MYH9, MAPRE1, ARHGEF7, TLE6, RASAL3, PLA2G4C, COBL, ACTR3C, CORO1C, CLIC5, FGF1, DBN1, NCL, CYTIP, SPTBN1, MLPH, EXOC8, MED28, SEPTIN7, CALD1, ITPR2, GIPC1, PARD6B, HIP1, RAC2, HIP1R, EXOC4, LANCL2, DLG4, FNBP1L, SEPTIN11, SHROOM3, HAX1, RHOB, PHLDB2, MYO10, DCDC2, SHROOM2, HMCN2, ADD3, FER, EPS8, DST, PLEKHH2, UTRN, PPP1R9A, CTBP1, GPSM1, SPTBN4, SPTA1, SEPTIN8, SHROOM1, DLC1, DENND2B, PRKCB, MYO1A, MYO5B, RHOH, STXBP6, PTK2, BFSP2, CFL1, RHOD, SPTBN2, CTBP2, TRAF6, RHOG, PDE4DIP, EXOC3, TRAK1, SEPTIN5, SEPTIN10, INSC, ASPH, UNC13B, MICAL3, MYZAP, LLGL1* |
| Focal adhesion | <0.0001 | 123 | 471 | 1.6231 | *ITGA2B, RALA, MRC2, CD44, ARHGAP31, TNC, CTNNA1, LIMA1, SYNE2, GDI2, RHOA, FGFR3, MAP4K4, ACTN1, LIMS2, FERMT2, HACD3, ACTB, APBB1IP, ITGA8, TNS1, EPB41L2, ITGB5, FAT1, CD59, NOX4, CASS4, ICAM1, SORBS1, NRP1, MISP, MYH9, FERMT1, MAPRE1, ARHGEF7, CORO2B, RPS16, HSPB1, LIMK1, HSPA8, LPXN, CD81, PPFIBP1, CORO1C, TNS2, ITGA4, IL1RL1, SORBS3, VASP, PTPN12, RAC2, FLNC, ARHGAP22, PALLD, NECTIN2, ARPC1B, MPRIP, KRAS, ANXA1, LMO7, TNS3, TLN1, SDCBP, KIF23, ITGA11, ITGAV, ARHGAP24, GIT2, ITGB7, IQGAP1, GRB7, HSPG2, RHOB, LIMD1, PHLDB2, LPP, ARHGAP26, G3BP1, EGFR, DIXDC1, ADAM17, DST, ENAH, FZD1, NEXN, XIRP2, ANXA5, RPS14, DLC1, YWHAZ, MAPRE2, IRF2, PTK2, LIMS1, YWHAG, TLN2, IL16, SYNPO2, CFL1, RPL38, PEAK1, CSRP2, RHOG, CALR, UBOX5, FLRT2, ATP6V0C, PIP5K1C, NHS, FOCAD, PARVB, EVL, AFAP1, MME, ANXA6, SVIL, PARVA, TGM2, SCARF2, PTPRC, ACTN4, ASAP3, FLII* |
| Cell-cell junction | <0.0001 | 131 | 519 | 1.5688 | *KRIT1, CYTH3, ABCC2, CTNNA1, TNK2, COL17A1, RHOA, PRKCZ, CDH19, ACTN1, LIMS2, PANX2, FERMT2, ACTB, CEACAM1, PKP1, GRHL2, FAT1, AKR1B1, FAT2, EPB41L4B, SORBS1, MYH9, JAG1, VAPA, LAMA1, CSK, NDRG1, SIPA1L3, DNMBP, CYTH1, GAB1, CCND1, LIN7A, VEGFA, ITK, CDH6, DBN1, CLDN16, FRMD4B, STRN, EPHA4, SSX2IP, TJP2, GJA8, PIK3CA, PARD6B, PLCG1, VASP, WNK4, RAP1B, FGF13, NECTIN2, PDLIM4, PATJ, APC, ANXA1, LMO7, NIBAN2, TLN1, SDCBP, TRPC6, ARHGAP24, SHROOM3, PPP3CA, IQGAP1, APP, PKP4, SCN1A, LIMD1, ILDR1, USP53, PIK3R1, SHROOM2, MTDH, ADD3, LIN7C, PTPRJ, ANK3, FRMD4A, ADAM17, SAV1, OBSCN, PDLIM3, CLDN8, TIAM1, KIT, SPTBN4, FGFR4, NEXN, VANGL2, PDLIM5, SHROOM1, GJB7, CLDN2, HEPACAM, JAM3, FRS2, CLMP, PLEKHA7, EVPL, STXBP6, LIMS1, CTNND2, CLDN20, PLEKHG5, FRMD5, SYNPO, PDZD3, PTPRM, HEG1, NECTIN3, KIRREL1, PKP3, CLDN6, FLRT2, PIP5K1C, MAGI2, AMTN, NHS, KAZN, COL13A1, NRAP, STARD10, AJM1, FMN1, SPECC1L-ADORA2A, PECAM1, PTPRK, DLL1, MAGI1* |
| Neuron projection | <0.0001 | 304 | 1444 | 1.3085 | *ALS2, CX3CL1, USH1C, PROM1, RUFY3, SAMD4A, SPAST, INSRR, USH2A, DTNBP1, MAP4, OPN3, KIF1B, CYFIP2, RASGRF1, PSD, AP3D1, MYO9A, RHOA, PRKCZ, SYT1, INPP5A, KCNAB2, CNGB1, MYO3B, MARK2, CRMP1, TP63, SRI, MARK3, ACTB, ITGA8, SLC1A3, ATP8B1, GSK3B, NCOA1, MTMR2, AURKA, NRCAM, RGS17, RPGRIP1, NRP1, PALMD, CBARP, CACNA1I, NIN, TRIM9, BDKRB1, ZMYND8, PLCB4, GUCY2F, RBM3, ARHGEF7, NECAB2, HOMER2, KCNN4, ANKRD27, MYH14, PTPRS, PPP2R1A, COBL, HSPB1, LIMK1, SLC1A1, ABHD17B, MAPK8, CDH23, BMPR1A, CHRNE, MPP2, ZPR1, HSPA8, SLC1A2, SLC38A1, RIPOR2, DBN1, TANC1, ITGA4, SPTBN1, MLPH, STRN, SLC1A4, AAK1, EPHA4, RAP1A, HDAC1, WLS, EXOC8, ADGRL2, KIF17, MPL, NRP2, CREB1, GOT1, MYOT, TSHZ3, GIPC1, PREX1, SLC12A5, STAU1, VAMP7, PACSIN1, CPNE5, PCSK2, KLC1, MCHR1, DOCK4, PALLD, KIF1C, FGF13, APOE, OLFM1, ATXN10, ASS1, HIP1R, PDLIM4, EXOC4, MAP1B, RARA, DNAJB1, SLC6A11, INPP5K, DDC, GUCY2D, DLG4, TERF2, AP3B1, PDE6A, ATP8A2, NTS, SBF2, CNTN6, IL6ST, NAV1, DTNA, HNF1A, HTR1B, CAPRIN1, DOCK10, SCN7A, BIN1, STXBP1, GABBR2, ARRB1, TTLL7, SEPTIN11, ANXA3, PPP3CA, PPFIA2, ABHD13, STON2, IQGAP1, KATNB1, ADCYAP1, MINK1, RPTOR, APP, AZIN2, ADCY10, KCNN3, CNIH3, SCN1A, SLC4A10, CAMK2D, GLRA3, MYO10, PAM, CPLX2, TENM2, DCDC2, TNFRSF21, TIAM2, NTRK2, RGR, HTR7, TENM4, NCAM1, ROM1, HNMT, DLG2, PIP4K2A, ANK3, EPS8, SAV1, DST, SPOCK1, FARP1, UTRN, ASAP1, ROBO4, GRIA1, ADCY8, TIAM1, RPGR, NPTN, NMNAT2, NRG1, DGKI, AUTS2, PPP1R9A, GDPD5, PINK1, SYNJ1, ATP13A2, RGS12, CFAP410, SPTBN4, BRSK1, DHRS3, NEXN, ADORA1, CCDC141, SPTA1, APBB2, IFT122, NPY5R, ADGRV1, SEPTIN8, GRIK2, HCN1, BAALC, HEPACAM, KCNC2, CKB, PRKCB, PRRT2, ROR2, RGS14, PTK2, SCN9A, TMEM266, NLGN1, CTNND2, FRMPD4, KISS1, EMB, KIF5B, FEZ2, NPTX1, LPAR3, PLEKHG5, GPHN, SYNPO, HCFC1, DAB1, CHRNA7, BASP1, NECTIN3, AP3S1, SLC17A8, SHARPIN, EXOC3, KCNIP1, TRAK1, KCNB2, MX2, PDE4B, OSBP2, SORCS2, ANKS1B, FLRT2, PRKN, ROR1, NTF3, AGBL4, BLOC1S4, GLDN, BCR, FSCN2, ESPN, MAGI2, BLOC1S2, MME, CACNA1H, LAMA2, NF1, STRN3, HTT, STMN3, MYO5A, SIPA1L1, SPG7, ERO1A, CEP290, UNC13B, OPA1, DMD, ITSN1, PRCD, GNAT3, ELFN1, SRGAP2, SRCIN1, ABR, CRHR1, APBA2, DRD4, NEFL, CYFIP1, IQCJ-SCHIP1, CHRNB1, LLGL1, CACNA1C, CLSTN3* |
| Synapse | <0.0001 | 303 | 1443 | 1.3051 | *ALS2, USH1C, MAPK8IP2, RNF216, SAMD4A, MUSK, RNF19A, INPP4A, USH2A, DTNBP1, KIF1B, CYFIP2, GDI2, PSD, GPC1, SNCAIP, AP3D1, MYO9A, RHOA, PRKCZ, SYT1, HDAC4, SDK2, KCNAB2, GNB5, CNGB1, NCK2, ACTN1, SRI, ACTB, PLD1, GPC4, ITGA8, AMPH, SYNJ2, SLC1A3, RIMS1, DLGAP4, MEF2C, CADPS2, GSK3B, ERC1, MTMR2, P2RX7, RAPGEF4, RGS17, RAB18, NRP1, PALMD, CBARP, MYH9, TRIM9, ZMYND8, EEF1A2, PLCB4, MAP1LC3A, ARHGEF7, TSC2, HOMER2, NDRG1, OLFM2, PTPRS, CHN2, PTPRZ1, SLC1A1, ABHD17B, DNMBP, MAPK8, FBXL20, RGS9, CHRNE, MPP2, USP46, HSPA8, SLC1A2, PPFIBP1, CORO1C, LIN7A, PPM1H, PHACTR1, QKI, ERBIN, PCDHB3, DBN1, FGF12, TANC1, SPTBN1, RTN4, GLS, STRN, SLC1A4, AAK1, EPHA4, TNR, RAP1A, ADGRL2, NRP2, PCDH17, BCL11A, GOT1, PDZRN3, GIPC1, SLC12A5, PLCG1, STAU1, VAMP7, PACSIN1, ZC4H2, HIP1, ARHGAP22, PALLD, APOE, OLFM1, LAMA5, HIP1R, SYNE1, PDLIM4, EXOC4, MAP1B, DNAJB1, SLC6A11, DDC, DLG4, AP3B1, DCLK1, NTS, CNTN6, NAPG, DTNA, ANXA1, HTR1B, CAPRIN1, DOCK10, SERPINE2, DGKB, BIN1, STXBP1, GABBR2, ARRB1, SDCBP, DTNB, KIAA1109, SEPTIN11, PPP3CA, PPFIA2, STON2, MCTP2, ADCYAP1, MINK1, CACNA1A, APP, ANXA9, CNIH3, PPFIA4, PKP4, SLC4A10, IQSEC1, GLRA3, CPLX2, TENM2, KCNMB1, TIAM2, SDK1, EGFR, NTRK2, CACNA1B, HTR7, ADD3, LIN7C, LRRC4C, DLG2, ITPR1, ANK3, EPS8, SPOCK1, SPARCL1, FARP1, UTRN, ASAP1, CEP112, WNT3A, ENAH, GRIA1, ADCY8, PSD3, KCNMA1, TIAM1, NPTN, SST, NMNAT2, ATP2B2, NRG1, DGKI, ACAN, PPP1R9A, SYNJ1, CTBP1, RGS12, BRSK1, PDLIM5, ADORA1, CADPS, APBB2, SLC9B2, NPY5R, ADGRV1, EGFLAM, SEPTIN8, GRIK2, DAGLB, RPS14, ADCY1, YWHAZ, BAALC, DACT1, RAPSN, KCNC2, GPR176, PPFIBP2, PRKCB, PRRT2, SLC3A2, STAT3, RGS14, PTK2, NLGN1, CTNND2, FRMPD4, YWHAG, EMB, NPTX1, KCNK3, CLCN5, LPAR3, GPHN, TLN2, SYNPO, RPL38, ABLIM3, SYT12, DAB1, CNP, SPTBN2, CTBP2, CHRNA7, MCTP1, KCMF1, NECTIN3, ERBB4, SLC17A8, SHARPIN, EXOC3, EXT1, PLCB1, MX2, PDE4B, SEPTIN5, SORCS2, ANKS1B, FLRT2, PRKN, ROR1, NTF3, SYN3, NRG3, PIP5K1C, ZNRF1, BCR, MAGI2, IL1RAP, MME, LAMA2, SLC6A17, HTT, COL13A1, SIPA1L1, ITSN2, UNC13B, DMD, PJA2, ITSN1, GPC2, VDAC1, ELFN1, STON1, IQSEC3, SEC22B, SRGAP2, DOC2B, PCDHB16, SRCIN1, PRIMA1, ABR, APBA2, DRD4, NEFL, CYFIP1, RPS6KA1, DLGAP2, PTPRD, CHRNB1, CACNA1C, CLSTN3* |
| I band | <0.0001 | 49 | 148 | 2.0578 | *SYNE2, HDAC4, ACTN1, FERMT2, SRI, PPP1R12B, NEBL, CACNA1S, FKBP1A, JPH1, DNAJB6, HSPB1, CASQ2, FKBP1B, MYOT, FLNC, PALLD, PDLIM4, MYO18B, FHOD3, CAB39, BIN1, PPP3CA, SCN1A, JPH2, ALDOA, ANK3, DST, OBSCN, PDLIM3, TTN, FBXO32, NEXN, XIRP2, PDLIM5, ANKRD2, SYNPO, MYOZ2, SYNPO2, CSRP2, PDE4B, AHNAK2, PARVB, MYH6, PARVA, NRAP, DMD, MYZAP, CACNA1C* |
| Stress fiber | <0.0001 | 29 | 71 | 2.5386 | *LIMA1, LIMCH1, MYLK, PRKCZ, ACTN1, FERMT2, NEBL, NOX4, SORBS1, ABLIM1, MYH9, DAAM1, MYH14, SIPA1L3, SEPTIN7, PALLD, PDLIM4, SEPTIN11, TPM1, LPP, PDLIM3, XIRP2, PDLIM5, PTK2, SYNPO, SYNPO2, ABLIM3, ROR1, MYH6* |
| Lamellipodium | <0.0001 | 63 | 214 | 1.8297 | *ALS2, RUFY3, CD44, ARHGAP31, CTNNA1, SYNE2, MYLK, RHOA, FERMT2, ACTB, APBB1IP, RAPGEF3, CARMIL1, FAT1, ABLIM1, ARHGEF7, PIEZO1, LIMK1, DOCK8, CORO1C, DBN1, PIK3CA, PLCG1, VAMP7, VASP, VIL1, RAC2, PALLD, PDLIM4, APC, CAPRIN1, ITGAV, FGD4, APP, CTTNBP2NL, HAX1, MYO10, IQGAP2, TIAM2, FER, PLEKHH2, ENAH, SH3RF1, FGD5, PPP1R9A, APBB2, INPPL1, ANTXR1, MCC, PLEKHG5, CFL1, ABLIM3, PTPRM, NHS, PARVB, EVL, PARVA, DMD, SNX2, ITSN1, DDX3X, ARPIN, SRGAP2* |
| Actin filament bundle | <0.0001 | 31 | 79 | 2.4389 | *LIMA1, LIMCH1, MYLK, PRKCZ, ACTN1, FERMT2, NEBL, NOX4, SORBS1, ABLIM1, MYH9, DAAM1, MYH14, SIPA1L3, SEPTIN7, VIL1, PALLD, PDLIM4, SEPTIN11, TPM1, LPP, PDLIM3, XIRP2, PDLIM5, PTK2, SYNPO, SYNPO2, ABLIM3, RFLNA, ROR1, MYH6* |
| Apical part of cell | <0.0001 | 113 | 453 | 1.5504 | *USH1C, PROM1, ABCC2, CD44, USH2A, PRKCZ, HYAL2, CYBRD1, CDHR2, PLD1, CEACAM1, ATP8B1, FAT1, CEACAM6, NOX4, SLC23A2, EPB41L4B, RAB18, NIN, JAG1, GPR143, HOMER2, ANXA13, SIPA1L3, SLC1A1, CUBN, SLC38A1, RIPOR2, CLIC5, PDE4D, ACVR1, FN1, IGFBP2, MREG, ECRG4, ABCC11, SEPTIN7, CNTFR, HVCN1, PARD6B, ABCC4, MYO1B, LDLR, HIP1R, PATJ, SLC14A2, KL, ANXA1, LMO7, SHROOM3, ENPEP, SLC39A8, SLC7A1, APP, ADCY10, HAX1, SLC4A10, CPO, OSMR, EGFR, SHROOM2, MTDH, MAL2, CRB2, SLC5A12, SLC7A11, ADAM17, KCNE4, FRMD1, SLC26A2, ADCY8, KCNMA1, NRG1, STC1, VANGL2, PLB1, SLC9B2, SHROOM1, SLC7A13, KCNC2, MYO1A, MYO5B, SLC3A2, SLC22A11, AHCYL1, ADRB2, MUC17, MYO7B, KISS1, CLCN5, PDZD3, DAB1, SPTBN2, SLC26A9, UMODL1, SVBP, DYNC2H1, NHS, OTOG, INSC, SLC4A5, SLC22A5, SLC22A12, SLC34A3, STK39, TMEM235, EMP2, GNAT3, AJM1, AQP1, DLL1, ABCC1, SLC16A1* |
| Contractile actin filament bundle | <0.0001 | 29 | 71 | 2.5386 | *LIMA1, LIMCH1, MYLK, PRKCZ, ACTN1, FERMT2, NEBL, NOX4, SORBS1, ABLIM1, MYH9, DAAM1, MYH14, SIPA1L3, SEPTIN7, PALLD, PDLIM4, SEPTIN11, TPM1, LPP, PDLIM3, XIRP2, PDLIM5, PTK2, SYNPO, SYNPO2, ABLIM3, ROR1, MYH6* |
| Z disc | <0.0001 | 45 | 136 | 2.0565 | *SYNE2, HDAC4, ACTN1, SRI, PPP1R12B, NEBL, FKBP1A, JPH1, DNAJB6, HSPB1, CASQ2, FKBP1B, MYOT, FLNC, PALLD, PDLIM4, MYO18B, FHOD3, CAB39, BIN1, PPP3CA, SCN1A, JPH2, ANK3, DST, OBSCN, PDLIM3, TTN, FBXO32, NEXN, XIRP2, PDLIM5, SYNPO, MYOZ2, SYNPO2, CSRP2, PDE4B, AHNAK2, PARVB, MYH6, PARVA, NRAP, DMD, MYZAP, CACNA1C* |
| Adherens junction | <0.0001 | 55 | 180 | 1.8991 | *CYTH3, CTNNA1, TNK2, CDH19, FERMT2, ACTB, CEACAM1, PKP1, FAT2, SORBS1, MYH9, JAG1, NDRG1, CYTH1, LIN7A, VEGFA, CDH6, FRMD4B, EPHA4, SSX2IP, TJP2, NECTIN2, PDLIM4, APC, ANXA1, LMO7, NIBAN2, TLN1, SDCBP, ARHGAP24, SHROOM3, PKP4, LIMD1, SHROOM2, LIN7C, FRMD4A, PDLIM3, SPTBN4, NEXN, PDLIM5, SHROOM1, FRS2, PLEKHA7, STXBP6, CTNND2, FRMD5, PTPRM, NECTIN3, PKP3, PIP5K1C, AJM1, FMN1, PTPRK, DLL1, MAGI1* |
| Actomyosin | <0.0001 | 31 | 80 | 2.4084 | *LIMA1, LIMCH1, MYLK, PRKCZ, HDAC4, ACTN1, FERMT2, NEBL, NOX4, SORBS1, ABLIM1, MYH9, DAAM1, MYH14, SIPA1L3, DBN1, SEPTIN7, PALLD, PDLIM4, SEPTIN11, TPM1, LPP, PDLIM3, XIRP2, PDLIM5, PTK2, SYNPO, SYNPO2, ABLIM3, ROR1, MYH6* |
| Apical plasma membrane | 0.0001 | 97 | 385 | 1.5659 | *PROM1, ABCC2, CD44, USH2A, PRKCZ, HYAL2, CYBRD1, CDHR2, PLD1, CEACAM1, ATP8B1, FAT1, CEACAM6, NOX4, SLC23A2, RAB18, JAG1, GPR143, ANXA13, SIPA1L3, SLC1A1, CUBN, SLC38A1, RIPOR2, CLIC5, PDE4D, FN1, IGFBP2, MREG, ECRG4, ABCC11, SEPTIN7, CNTFR, HVCN1, PARD6B, ABCC4, HIP1R, PATJ, SLC14A2, KL, ANXA1, LMO7, SHROOM3, ENPEP, SLC39A8, SLC7A1, ADCY10, HAX1, SLC4A10, CPO, OSMR, EGFR, SHROOM2, MTDH, MAL2, CRB2, SLC5A12, ADAM17, KCNE4, FRMD1, SLC26A2, ADCY8, KCNMA1, NRG1, STC1, VANGL2, PLB1, SLC9B2, SHROOM1, SLC7A13, KCNC2, MYO1A, SLC3A2, SLC22A11, AHCYL1, ADRB2, MUC17, KISS1, PDZD3, SPTBN2, SLC26A9, UMODL1, NHS, OTOG, SLC4A5, SLC22A5, SLC22A12, SLC34A3, STK39, TMEM235, EMP2, GNAT3, AJM1, AQP1, DLL1, ABCC1, SLC16A1* |
| Axon | 0.0002 | 156 | 691 | 1.4032 | *ALS2, RUFY3, SPAST, INSRR, USH2A, DTNBP1, MAP4, KIF1B, RASGRF1, AP3D1, MYO9A, RHOA, PRKCZ, SYT1, KCNAB2, CNGB1, CRMP1, SRI, ACTB, GSK3B, MTMR2, AURKA, NRCAM, NRP1, CBARP, NIN, NECAB2, MYH14, PTPRS, COBL, HSPB1, SLC1A1, MAPK8, ZPR1, HSPA8, SLC1A2, SLC38A1, DBN1, TANC1, ITGA4, SPTBN1, AAK1, EPHA4, EXOC8, ADGRL2, NRP2, CREB1, GOT1, MYOT, TSHZ3, PREX1, PACSIN1, KLC1, PALLD, KIF1C, FGF13, OLFM1, EXOC4, MAP1B, DDC, DLG4, TERF2, AP3B1, NTS, SBF2, CNTN6, NAV1, DTNA, HTR1B, SCN7A, BIN1, STXBP1, SEPTIN11, ANXA3, PPFIA2, IQGAP1, KATNB1, ADCYAP1, MINK1, APP, AZIN2, ADCY10, SCN1A, SLC4A10, CPLX2, TENM2, TNFRSF21, TIAM2, NTRK2, DLG2, ANK3, EPS8, DST, SPOCK1, UTRN, ROBO4, GRIA1, ADCY8, TIAM1, NPTN, NMNAT2, NRG1, DGKI, AUTS2, PPP1R9A, GDPD5, PINK1, SYNJ1, SPTBN4, BRSK1, NEXN, ADORA1, SPTA1, APBB2, SEPTIN8, GRIK2, HCN1, HEPACAM, KCNC2, PRKCB, PRRT2, SCN9A, EMB, KIF5B, FEZ2, LPAR3, PLEKHG5, HCFC1, BASP1, NECTIN3, AP3S1, SLC17A8, EXOC3, TRAK1, MX2, ROR1, NTF3, AGBL4, BLOC1S4, GLDN, BCR, BLOC1S2, MME, NF1, HTT, STMN3, MYO5A, SPG7, UNC13B, OPA1, DMD, SRCIN1, ABR, NEFL, IQCJ-SCHIP1, LLGL1* |
| Basement membrane | 0.0002 | 34 | 97 | 2.1785 | *TNC, USH2A, LAMC2, COL17A1, FBLN1, P3H2, LAMB4, LAMB1, LAMA1, APLP1, ATRNL1, ERBIN, FN1, NID1, TGFBI, COL5A1, LAMA5, LOXL2, LAMC1, HSPG2, TMEFF2, COL8A1, HMCN2, FREM2, DST, ACAN, LAD1, EGFLAM, FBN1, EFEMP2, MMRN2, AMTN, LAMA2, COL28A1* |
| Myofibril | 0.0008 | 65 | 244 | 1.6557 | *SYNE2, HDAC4, ACTN1, FERMT2, SRI, PPP1R12B, NEBL, CACNA1S, FKBP1A, TNNC2, MYOM1, JPH1, DNAJB6, HSPB1, CORO1C, SPTBN1, CASQ2, TRIM32, FKBP1B, MYOT, CALD1, FLNC, PALLD, MYOD1, SYNE1, PDLIM4, MYO18B, MYBPC3, FHOD3, CAB39, BIN1, PPP3CA, TPM1, MYOM3, SCN1A, MYH15, JPH2, ALDOA, ANK3, DST, OBSCN, PDLIM3, TTN, FBXO32, SQSTM1, NEXN, XIRP2, PDLIM5, ANKRD2, MYL1, SYNPO, MYOZ2, SYNPO2, CSRP2, PDE4DIP, PDE4B, AHNAK2, PARVB, SVIL, MYH6, PARVA, NRAP, DMD, MYZAP, CACNA1C* |
| Plasma membrane region | 0.0008 | 268 | 1323 | 1.2590 | *TFPI, RALA, PROM1, ABCC2, CD44, MUSK, USH2A, CTNNA1, DTNBP1, LIMA1, SYNE2, PSD, TNK2, SNCAIP, ERBB3, MYLK, RHOA, PRKCZ, SYT1, HYAL2, CNGB1, CYBRD1, EVC, FERMT2, CDHR2, PLD1, GPC4, ITGA8, SLC1A3, CEACAM1, RIMS1, SLC4A4, ATP8B1, ERC1, FAT1, CEACAM6, NOX4, MTMR2, SLC23A2, RAB18, NRP1, PDGFB, MYH9, FERMT1, MAPRE1, JAG1, GPR143, BMX, PIEZO1, ANXA13, PTPRS, SIPA1L3, IQCE, SLC1A1, DOCK8, KANK1, ABHD17B, PKD2L1, CUBN, BMPR1A, RGS9, CHRNE, ABCC3, MPP2, HSPA8, SLC1A2, CD81, CORO1C, LIN7A, SLC38A1, RIPOR2, CLIC5, ERBIN, PDE4D, WWC1, DBN1, SPTBN1, FN1, IGFBP2, STRN, AAK1, EPHA4, WLS, ADGRL2, KIF17, MREG, NRP2, SGIP1, PCDH17, ECRG4, ABCC11, BBS9, SEPTIN7, CNTFR, HVCN1, PARD6B, PLCG1, PTGIS, PACSIN1, ABCC4, PSD4, VASP, ZC4H2, EPS15L1, MACF1, HIP1, MCHR1, LDLR, HIP1R, SYNE1, EPS8L1, TBC1D5, PDLIM4, SLC6A11, INPP5K, GUCY2D, DLG4, PATJ, SLC14A2, PDE6A, KL, CNTN6, APC, ANXA1, HTR1B, LMO7, DGKB, STXBP1, GABBR2, TLN1, ARRB1, CLCA2, MYOF, ITGAV, SLC40A1, SHROOM3, ENPEP, SLC39A8, SLC7A1, TPM1, APP, ADCY10, HAX1, CNIH3, RHOB, SLC4A10, CPO, GLRA3, MYO10, OSMR, TENM2, EGFR, SHROOM2, MTDH, MAL2, CRB2, HMCN2, SLC5A12, LIN7C, LRRC4C, PTPRJ, ROM1, DLG2, SLC7A11, ANK3, EPS8, ADAM17, DST, KCNE4, AP1S3, FARP1, UTRN, FRMD1, ASAP1, CD8A, FGD5, SLC7A7, GRIA1, SLC26A2, ADCY8, PSD3, KCNMA1, CLDN8, TIAM1, NPTN, ATP2B2, NRG1, DGKI, PKD1L1, SYNJ1, STC1, DHRS3, VANGL2, ADORA1, PLB1, SLC9B2, ADGRV1, SHROOM1, GRIK2, DAGLB, DLC1, ADCY1, NOS3, SLC7A13, ABCA1, RAPSN, KCNC2, MYO1A, PRRT2, SLC3A2, SLC22A11, AHCYL1, IRS1, ADRB2, ARL13B, ANTXR1, NLGN1, MUC17, KISS1, GPHN, PDZD3, CFL1, SPTBN2, SLC26A9, CHRNA7, UMODL1, NECTIN3, ERBB4, EXOC3, GPR157, MX2, KIRREL1, EFNA5, CLDN6, SORCS2, PIP5K1C, NHS, OTOG, ASAH2, SLC4A5, SVIL, LRP10, SLC22A5, COL13A1, SLC28A3, SLC22A12, PLCG2, ITSN2, SLC34A3, STK39, UNC13B, ALPK2, DMD, TMEM235, ITSN1, EMP2, PRCD, GNAT3, AJM1, AQP1, SRGAP2, DLL1, ABCC1, DGKD, SLC16A1, CHRNB1, CACNA1C, CLSTN3* |
| Sarcomere | 0.0008 | 60 | 221 | 1.6874 | *SYNE2, HDAC4, ACTN1, FERMT2, SRI, PPP1R12B, NEBL, CACNA1S, FKBP1A, TNNC2, MYOM1, JPH1, DNAJB6, HSPB1, CORO1C, SPTBN1, CASQ2, TRIM32, FKBP1B, MYOT, FLNC, PALLD, SYNE1, PDLIM4, MYO18B, MYBPC3, FHOD3, CAB39, BIN1, PPP3CA, TPM1, MYOM3, SCN1A, JPH2, ALDOA, ANK3, DST, OBSCN, PDLIM3, TTN, FBXO32, SQSTM1, NEXN, XIRP2, PDLIM5, ANKRD2, MYL1, SYNPO, MYOZ2, SYNPO2, CSRP2, PDE4B, AHNAK2, PARVB, MYH6, PARVA, NRAP, DMD, MYZAP, CACNA1C* |
| Glutamatergic synapse | 0.0008 | 88 | 358 | 1.5278 | *RNF216, RNF19A, DTNBP1, AP3D1, RHOA, PRKCZ, SYT1, ACTN1, ACTB, GPC4, DLGAP4, CADPS2, GSK3B, NRP1, PLCB4, HOMER2, NDRG1, PTPRS, ABHD17B, FBXL20, RGS9, MPP2, USP46, HSPA8, SLC1A2, PPM1H, ERBIN, DBN1, SPTBN1, EPHA4, TNR, RAP1A, ADGRL2, NRP2, PCDH17, GIPC1, PLCG1, STAU1, HIP1, ARHGAP22, APOE, DNAJB1, DLG4, DGKB, STXBP1, SEPTIN11, PPP3CA, PPFIA2, CPLX2, LIN7C, LRRC4C, EPS8, SPARCL1, FARP1, WNT3A, GRIA1, ADCY8, TIAM1, NPTN, ATP2B2, NRG1, DGKI, ACAN, PPP1R9A, CTBP1, CADPS, GRIK2, ADCY1, YWHAZ, PRRT2, STAT3, RGS14, NLGN1, FRMPD4, NPTX1, ABLIM3, SPTBN2, CTBP2, ERBB4, PLCB1, SYN3, NRG3, BCR, IL1RAP, SLC6A17, ITSN1, ABR, PTPRD* |
| Neuron to neuron synapse | 0.0009 | 93 | 385 | 1.5013 | *ALS2, MAPK8IP2, RNF19A, INPP4A, DTNBP1, PSD, PRKCZ, SYT1, NCK2, ITGA8, MTMR2, RAPGEF4, PLCB4, TSC2, HOMER2, PTPRS, SLC1A1, ABHD17B, RGS9, MPP2, LIN7A, DBN1, TANC1, SPTBN1, RTN4, STRN, EPHA4, BCL11A, VAMP7, HIP1R, MAP1B, DNAJB1, DLG4, DCLK1, ARRB1, DTNB, SEPTIN11, MINK1, PKP4, IQSEC1, NTRK2, ADD3, LIN7C, LRRC4C, DLG2, ITPR1, EPS8, SPOCK1, GRIA1, ADCY8, PSD3, TIAM1, NPTN, ATP2B2, NRG1, DGKI, PPP1R9A, PDLIM5, ADORA1, GRIK2, RPS14, ADCY1, YWHAZ, BAALC, PRRT2, STAT3, RGS14, NLGN1, CTNND2, FRMPD4, GPHN, SYNPO, RPL38, SYT12, DAB1, NECTIN3, ERBB4, SHARPIN, MX2, PDE4B, SORCS2, ANKS1B, PRKN, SYN3, BCR, MAGI2, SIPA1L1, DMD, PJA2, IQSEC3, SRGAP2, SRCIN1, CACNA1C* |
| Postsynapse | 0.0014 | 149 | 682 | 1.3579 | *ALS2, MAPK8IP2, RNF216, MUSK, RNF19A, INPP4A, DTNBP1, PSD, AP3D1, RHOA, PRKCZ, NCK2, SRI, ACTB, ITGA8, DLGAP4, MEF2C, GSK3B, MTMR2, P2RX7, NRP1, PALMD, ZMYND8, PLCB4, ARHGEF7, TSC2, HOMER2, PTPRS, SLC1A1, ABHD17B, RGS9, CHRNE, MPP2, HSPA8, LIN7A, ERBIN, DBN1, TANC1, SPTBN1, RTN4, STRN, EPHA4, ADGRL2, NRP2, PCDH17, BCL11A, GIPC1, ZC4H2, HIP1, HIP1R, SYNE1, PDLIM4, MAP1B, DNAJB1, SLC6A11, DLG4, DCLK1, DOCK10, DGKB, STXBP1, GABBR2, ARRB1, DTNB, SEPTIN11, PPP3CA, PPFIA2, MINK1, APP, CNIH3, PKP4, SLC4A10, IQSEC1, GLRA3, CPLX2, TENM2, NTRK2, ADD3, LIN7C, LRRC4C, DLG2, ITPR1, ANK3, EPS8, SPOCK1, FARP1, UTRN, ASAP1, GRIA1, ADCY8, PSD3, KCNMA1, TIAM1, NPTN, ATP2B2, NRG1, DGKI, PPP1R9A, PDLIM5, ADORA1, GRIK2, DAGLB, RPS14, ADCY1, BAALC, RAPSN, KCNC2, PRRT2, STAT3, RGS14, PTK2, NLGN1, CTNND2, FRMPD4, GPHN, SYNPO, RPL38, DAB1, SPTBN2, CHRNA7, NECTIN3, ERBB4, SHARPIN, PLCB1, MX2, PDE4B, SORCS2, ANKS1B, PRKN, SYN3, BCR, MAGI2, LAMA2, SLC6A17, HTT, COL13A1, SIPA1L1, DMD, PJA2, ITSN1, IQSEC3, SRGAP2, SRCIN1, ABR, APBA2, DRD4, NEFL, CHRNB1, CACNA1C, CLSTN3* |
| Cortical actin cytoskeleton | 0.0014 | 32 | 98 | 2.0295 | *ANLN, LLGL2, RAPGEF3, EPB41L2, MISP, MYH9, COBL, ACTR3C, DBN1, SPTBN1, MLPH, MED28, CALD1, HIP1, HIP1R, LANCL2, SHROOM3, DCDC2, SHROOM2, PLEKHH2, UTRN, PPP1R9A, SPTBN4, SPTA1, SHROOM1, DLC1, PRKCB, MYO1A, CFL1, SPTBN2, MYZAP, LLGL1* |
| Somatodendritic compartment | 0.0016 | 191 | 913 | 1.3002 | *ALS2, CX3CL1, MAPK8IP2, RUFY3, SAMD4A, USH2A, DTNBP1, KIF1B, PSD, SNCAIP, RHOA, PRKCZ, INPP5A, MARK2, CRMP1, TP63, SLC24A1, SRI, MARK3, ITGA8, SLC1A3, GSK3B, APOB, MTMR2, AURKA, EFHC1, NRP1, PALMD, NIN, TRIM9, ZMYND8, EEF1A2, PLCB4, RBM3, ARHGEF7, NECAB2, HOMER2, KCNN4, PTPRS, PPP2R1A, COBL, SLC1A1, ABHD17B, MAPK8, BMPR1A, MPP2, ZPR1, HSPA8, SLC38A1, DBN1, TANC1, ITGA4, PDE1A, RTN4, MLPH, STRN, SLC1A4, EPHA4, HDAC1, WLS, KIF17, MPL, TGFB3, MYOT, IAPP, GIPC1, PREX1, SLC12A5, STAU1, CPNE5, PCSK2, KIF1C, FGF13, LDLR, APOE, TRPM4, OLFM1, ATXN10, ASS1, HIP1R, PDLIM4, MAP1B, RARA, DNAJB1, DDC, DLG4, IL6ST, HTR1B, CAPRIN1, DOCK10, BIN1, ARRB1, TTLL7, SEPTIN11, ANXA3, PPP3CA, PPFIA2, ASCL1, ABHD13, PMM2, KATNB1, ADCYAP1, MINK1, RPTOR, CACNA1A, APP, AZIN2, ADCY10, KCNN3, CNIH3, SCN1A, SLC4A10, GLRA3, MYO10, PAM, G3BP1, CPLX2, TENM2, TIAM2, NTRK2, HTR7, DLG2, ANK3, SAV1, FARP1, ASAP1, PDE1C, GRIA1, ADCY8, TIAM1, NPTN, SST, DGKI, PPP1R9A, ATP13A2, RGS12, SPTBN4, ADORA1, GRIK2, HCN1, KCNC2, CKB, PRRT2, SLC3A2, ROR2, RGS14, PTK2, TMEM266, NLGN1, CTNND2, FRMPD4, KISS1, KIF5B, GPHN, SYNPO, HCFC1, DAB1, SPTBN2, NECTIN3, SLC17A8, SHARPIN, KCNIP1, TRAK1, KCNB2, MX2, CCR4, PDE4B, OSBP2, SORCS2, ANKS1B, NTF3, BCR, MAGI2, MME, LAMA2, NF1, STRN3, HTT, SIPA1L1, ERO1A, OPA1, DMD, ITSN1, ELFN1, SRGAP2, SRCIN1, ABR, APBA2, DRD4, CACNA1C, CLSTN3* |
| Postsynaptic density | 0.0018 | 85 | 352 | 1.5008 | *ALS2, MAPK8IP2, INPP4A, DTNBP1, PSD, PRKCZ, NCK2, ITGA8, MTMR2, PLCB4, TSC2, HOMER2, PTPRS, ABHD17B, RGS9, MPP2, LIN7A, DBN1, TANC1, SPTBN1, RTN4, STRN, EPHA4, BCL11A, HIP1R, MAP1B, DNAJB1, DLG4, DCLK1, ARRB1, DTNB, MINK1, PKP4, IQSEC1, NTRK2, ADD3, LIN7C, LRRC4C, DLG2, ITPR1, EPS8, SPOCK1, GRIA1, ADCY8, PSD3, TIAM1, NPTN, ATP2B2, NRG1, DGKI, PPP1R9A, PDLIM5, ADORA1, GRIK2, RPS14, ADCY1, BAALC, PRRT2, STAT3, RGS14, NLGN1, CTNND2, FRMPD4, GPHN, SYNPO, RPL38, DAB1, NECTIN3, ERBB4, SHARPIN, MX2, PDE4B, SORCS2, ANKS1B, PRKN, SYN3, BCR, MAGI2, SIPA1L1, DMD, PJA2, IQSEC3, SRGAP2, SRCIN1, CACNA1C* |
| Ruffle | 0.0019 | 52 | 191 | 1.6921 | *ALS2, CYTH3, LIMA1, PSD, RHOA, ACTN1, SNX5, MYH9, FERMT1, BMX, ARHGEF7, CDK6, COBL, KANK1, CORO1C, WWC1, FRMD4B, WIPF1, PLCG1, PACSIN1, PSD4, MACF1, VIL1, PALLD, CLIP1, HIP1R, EPS8L1, INPP5K, APC, TLN1, ITGAV, FGD4, TPM1, IQGAP1, MYO10, RASA1, ARHGAP18, EGFR, PTPRJ, EPS8, ADAM17, FGD5, PSD3, TIAM1, DLC1, MTSS1, TLN2, CFL1, PIP5K1C, MYO5A, PLCG2, ASAP3* |
| Microtubule cytoskeleton | 0.0019 | 276 | 1391 | 1.2332 | *MAD1L1, ALS2, CDC27, VPS41, DNAH9, NISCH, DDX11, TACC3, CCDC88C, SPAST, RNF19A, CUL3, USH2A, CNTLN, DTNBP1, MAP4, MPHOSPH9, FNIP2, KIF1B, CROCC, EML1, KIF26A, PRKCZ, KIF2A, KCNAB2, CRMP1, EVC, FRY, WDR62, KIF22, SCTR, CDC14B, GSK3B, ERC1, PIBF1, KIF3C, TTC39A, MAST2, RTRAF, AURKA, ANAPC5, PPP2R3C, SORBS1, EFHC1, IFT74, MISP, CEP170B, CYTH4, TTC28, MYH9, IFT27, POLR3H, NIN, KIAA0586, DAAM1, PCIF1, RAE1, MAPRE1, MAP1LC3A, VAPA, TBL1X, ARHGEF7, PARP4, KATNAL1, NUP93, CSPP1, NDRG1, CCDC61, PPP2R1A, CDK6, DNAH11, HSPB1, AKNA, CCNJ, CCSER2, ANKRD26, B9D1, SLAIN2, CTSC, CEP85L, RAB23, CLIC5, PDE4D, IQCG, CLIP4, RMDN2, SLC1A4, ACTL8, SSX2IP, KIF17, CENPF, STAG1, CAMSAP2, DNAH7, CNTRL, NEK9, IFT43, CCDC170, ANXA11, BBS9, SEPTIN7, STIL, STAU1, FAM110A, KLC1, MACF1, KIF1C, MTUS1, FGF13, CAMSAP1, TUBGCP2, CLIP1, SYNE1, MAP1B, VPS4A, PATJ, PSRC1, NAV1, KCTD1, HOOK1, TPGS2, DZIP1, APC, CCDC146, CEP162, DYSF, TTLL4, ODF2, CDK5RAP2, KIF12, KIF13A, NEK1, DCUN1D5, MAPKBP1, KIF23, HAUS2, LRRC49, TTLL7, TACC2, HECW2, SEPTIN11, CDKL2, SHROOM3, BRCA2, IQGAP1, KATNB1, BCAS3, SS18, SMAD4, MVB12A, DPP9, APP, ADCY10, LYST, PKP4, TBCK, IQGAP2, TBC1D7, DCDC2, ARHGAP18, SHROOM2, HMBOX1, TRIM55, CHEK1, HNMT, NEK7, FER, DST, TUBA3E, HAUS1, SPEF2, AK5, DNAAF1, CEP112, TSEN2, SPAG17, CFAP70, WHAMM, CCT8, TIAM1, RPGR, TBC1D31, BUB1B, RAB28, EYA3, DNAH3, SYNJ1, CFAP410, BRSK1, MAPKAPK2, PBXIP1, CCT3, CCDC141, PPP4R2, ATXN7, IFT122, POC1A, SEPTIN8, SHROOM1, GEM, KIF27, PKNOX2, EML5, RAPSN, CEP295, CEP57, TBATA, CLMP, PLEKHA7, DCTN5, MAPRE2, TUBA1C, E4F1, PXK, AXIN2, CEP120, ROR2, RGS14, PTK2, CCDC8, CDK1, TTLL6, KIF5B, HSPA6, CNP, THAP6, JAKMIP2, TBL1XR1, PDE4DIP, RPP25, DNHD1, HARBI1, MAP6D1, PLAG1, RELL1, IST1, FIGN, LCK, CABCOCO1, MX2, FBXL7, DNAH2, CLECL1, PDE4B, SEPTIN5, TEDC1, AGBL4, SEPTIN10, DYNC2H1, HEPACAM2, GTF2F2, BLOC1S2, SVIL, HTT, SFI1, UVRAG, ITSN2, CEP290, PRC1, TRAF3IP1, PTPN20, EML6, ZBED1, UBXN2B, DDX3X, KIF4B, MICAL3, MAP1LC3B2, SPECC1L-ADORA2A, CCDC187, RASSF5, ARHGEF10, PPP4R3B, RCC2, SLC16A1, FLII* |
| Asymmetric synapse | 0.0019 | 86 | 358 | 1.4931 | *ALS2, MAPK8IP2, INPP4A, DTNBP1, PSD, PRKCZ, NCK2, ITGA8, MTMR2, PLCB4, TSC2, HOMER2, PTPRS, SLC1A1, ABHD17B, RGS9, MPP2, LIN7A, DBN1, TANC1, SPTBN1, RTN4, STRN, EPHA4, BCL11A, HIP1R, MAP1B, DNAJB1, DLG4, DCLK1, ARRB1, DTNB, MINK1, PKP4, IQSEC1, NTRK2, ADD3, LIN7C, LRRC4C, DLG2, ITPR1, EPS8, SPOCK1, GRIA1, ADCY8, PSD3, TIAM1, NPTN, ATP2B2, NRG1, DGKI, PPP1R9A, PDLIM5, ADORA1, GRIK2, RPS14, ADCY1, BAALC, PRRT2, STAT3, RGS14, NLGN1, CTNND2, FRMPD4, GPHN, SYNPO, RPL38, DAB1, NECTIN3, ERBB4, SHARPIN, MX2, PDE4B, SORCS2, ANKS1B, PRKN, SYN3, BCR, MAGI2, SIPA1L1, DMD, PJA2, IQSEC3, SRGAP2, SRCIN1, CACNA1C* |
| Contractile fiber | 0.0019 | 65 | 253 | 1.5968 | *SYNE2, HDAC4, ACTN1, FERMT2, SRI, PPP1R12B, NEBL, CACNA1S, FKBP1A, TNNC2, MYOM1, JPH1, DNAJB6, HSPB1, CORO1C, SPTBN1, CASQ2, TRIM32, FKBP1B, MYOT, CALD1, FLNC, PALLD, MYOD1, SYNE1, PDLIM4, MYO18B, MYBPC3, FHOD3, CAB39, BIN1, PPP3CA, TPM1, MYOM3, SCN1A, MYH15, JPH2, ALDOA, ANK3, DST, OBSCN, PDLIM3, TTN, FBXO32, SQSTM1, NEXN, XIRP2, PDLIM5, ANKRD2, MYL1, SYNPO, MYOZ2, SYNPO2, CSRP2, PDE4DIP, PDE4B, AHNAK2, PARVB, SVIL, MYH6, PARVA, NRAP, DMD, MYZAP, CACNA1C* |
| Cortical cytoskeleton | 0.0020 | 38 | 127 | 1.8597 | *ANLN, LLGL2, ACTB, RAPGEF3, EPB41L2, RIMS1, ERC1, MISP, MYH9, MAPRE1, COBL, ACTR3C, DBN1, SPTBN1, MLPH, MED28, CALD1, HIP1, HIP1R, LANCL2, DLG4, SHROOM3, DCDC2, SHROOM2, PLEKHH2, UTRN, PPP1R9A, SPTBN4, SPTA1, SHROOM1, DLC1, PRKCB, MYO1A, CFL1, SPTBN2, PDE4DIP, MYZAP, LLGL1* |
| Postsynaptic specialization | 0.0023 | 89 | 376 | 1.4712 | *ALS2, MAPK8IP2, INPP4A, DTNBP1, PSD, PRKCZ, NCK2, ITGA8, DLGAP4, MTMR2, PLCB4, TSC2, HOMER2, PTPRS, ABHD17B, RGS9, MPP2, HSPA8, LIN7A, DBN1, TANC1, SPTBN1, RTN4, STRN, EPHA4, BCL11A, HIP1R, MAP1B, DNAJB1, DLG4, DCLK1, ARRB1, DTNB, SEPTIN11, MINK1, PKP4, IQSEC1, NTRK2, ADD3, LIN7C, LRRC4C, DLG2, ITPR1, EPS8, SPOCK1, GRIA1, ADCY8, PSD3, TIAM1, NPTN, ATP2B2, NRG1, DGKI, PPP1R9A, PDLIM5, ADORA1, GRIK2, RPS14, ADCY1, BAALC, RAPSN, PRRT2, STAT3, RGS14, NLGN1, CTNND2, FRMPD4, GPHN, SYNPO, RPL38, DAB1, NECTIN3, ERBB4, SHARPIN, MX2, PDE4B, SORCS2, ANKS1B, PRKN, SYN3, BCR, MAGI2, SIPA1L1, DMD, PJA2, IQSEC3, SRGAP2, SRCIN1, CACNA1C* |
| Dendritic spine | 0.0023 | 52 | 193 | 1.6746 | *ALS2, DTNBP1, PSD, RHOA, SRI, ITGA8, MTMR2, PALMD, ZMYND8, SLC1A1, ABHD17B, MPP2, STRN, EPHA4, GIPC1, HIP1R, PDLIM4, MAP1B, DNAJB1, DLG4, DOCK10, ARRB1, SEPTIN11, PPP3CA, PPFIA2, APP, TENM2, NTRK2, FARP1, ASAP1, GRIA1, TIAM1, DGKI, PPP1R9A, ADORA1, PRRT2, RGS14, PTK2, NLGN1, FRMPD4, SYNPO, MX2, PDE4B, SORCS2, ANKS1B, BCR, LAMA2, SIPA1L1, ITSN1, SRGAP2, ABR, APBA2* |
| Neuron spine | 0.0035 | 52 | 196 | 1.6489 | *ALS2, DTNBP1, PSD, RHOA, SRI, ITGA8, MTMR2, PALMD, ZMYND8, SLC1A1, ABHD17B, MPP2, STRN, EPHA4, GIPC1, HIP1R, PDLIM4, MAP1B, DNAJB1, DLG4, DOCK10, ARRB1, SEPTIN11, PPP3CA, PPFIA2, APP, TENM2, NTRK2, FARP1, ASAP1, GRIA1, TIAM1, DGKI, PPP1R9A, ADORA1, PRRT2, RGS14, PTK2, NLGN1, FRMPD4, SYNPO, MX2, PDE4B, SORCS2, ANKS1B, BCR, LAMA2, SIPA1L1, ITSN1, SRGAP2, ABR, APBA2* |
| Endoplasmic reticulum lumen | 0.0048 | 78 | 328 | 1.4780 | *FMO1, TNC, LTBP1, COL23A1, PDIA5, COL17A1, APOB, TXNDC16, P3H2, LAMB1, TSPAN15, SERPIND1, PDGFB, FOXRED2, STS, MINPP1, CTSC, WNT5B, COL12A1, ARSB, STC2, EVA1A, FN1, P3H1, CASQ2, P4HA1, COL21A1, SDF2L1, VGF, CALU, APOE, COLGALT1, COL5A1, ENAM, POGLUT2, GOLM1, LAMC1, CKAP4, ADAMTS7, UGGT1, SLC27A2, MFGE8, ARSG, IGFBP4, FKBP10, APP, SUMF1, COL8A1, PDGFC, SERPING1, PRSS23, ADAM17, TGOLN2, SPARCL1, WNT3A, COL6A3, MELTF, LIPC, FBN1, TSPAN5, PCSK9, SDC2, FGG, ADAMTSL1, CALR, ARSJ, PDIA2, TOR3A, AMTN, COL14A1, COL25A1, COL27A1, COL13A1, ERO1A, ASPH, COLGALT2, COL5A2, COL28A1* |
| Supramolecular fiber | 0.0065 | 220 | 1102 | 1.2408 | *DNAH9, DCN, SPAST, CUL3, ARHGAP6, MAP4, LTBP1, ELN, KIF1B, SYNE2, EML1, KIF26A, HDAC4, KIF2A, KCNAB2, ACTN1, MARK2, FERMT2, SRI, ACTB, PPP1R12B, FBLN1, NEBL, KIF22, CARMIL1, SCTR, CACNA1S, PKP1, KIF3C, AURKA, FKBP1A, NRP1, MYO9B, MISP, CEP170B, MYH9, NIN, MAPRE1, MAP1LC3A, TNNC2, MYOM1, PARP4, KATNAL1, CSPP1, JPH1, NDRG1, MYH14, DNAH11, DNAJB6, COBL, HSPB1, AKNA, AK1, SLAIN2, CORO1C, LMNB1, DBN1, CLIP4, SPTBN1, RMDN2, SLC1A4, WIPF1, KIF17, CAMSAP2, CASQ2, DNAH7, TRIM32, FKBP1B, MYOT, WIPF3, CALD1, KLC1, MACF1, RAC2, FLNC, MYO1B, PALLD, MYOD1, KIF1C, MTUS1, FGF13, CAMSAP1, COL5A1, TUBGCP2, CLIP1, SYNE1, GFAP, PDLIM4, MAP1B, NES, MYO18B, PSRC1, NAV1, MYBPC3, HOOK1, FHOD3, TPGS2, APC, ANXA1, CEP162, TTLL4, CAB39, BIN1, ODF2, CDK5RAP2, KIF12, KIF13A, KIF23, HAUS2, LRRC49, TTLL7, SHROOM3, PPP3CA, FBN2, TPM1, IQGAP1, KATNB1, BCAS3, DPP9, ADAMTS10, MYOM3, FLG, SCN1A, MYH15, IQGAP2, DCDC2, ARHGAP18, SHROOM2, TRIM55, JPH2, ALDOA, ANK3, NEK7, DST, TUBA3E, HAUS1, OBSCN, PDLIM3, TTN, SPAG17, WHAMM, CCT8, TIAM1, FBXO32, DNAH3, SYNJ1, SPTBN4, LMNA, SQSTM1, NEXN, XIRP2, PDLIM5, PBXIP1, COL6A3, CCT3, SHROOM1, KIF27, EML5, ANKRD2, CEP295, CEP57, JAM3, FBN1, CLMP, MYO1A, MAPRE2, TUBA1C, EVPL, MYL1, ROR2, RGS14, CDK1, TTLL6, KIF5B, BFSP2, SYNPO, MYOZ2, SYNPO2, EFEMP2, CNP, CSRP2, LMNB2, PDE4DIP, MAP6D1, FIGN, DNAH2, PDE4B, AHNAK2, ESPN, DYNC2H1, THSD4, PARVB, COL27A1, KRT35, SVIL, MYO5A, MFAP5, MYH6, PARVA, NRAP, PRC1, DMD, PTPN20, COL5A2, EML6, KIF4B, FMN1, MAP1LC3B2, KRTAP3-1, KRT39, KRT33B, MYZAP, KRTAP4-11, KRTAP4-12, RASSF5, KRTAP10-7, KRTAP5-5, NEFL, RCC2, CACNA1C* |
| Supramolecular polymer | 0.0072 | 221 | 1110 | 1.2375 | *DNAH9, DCN, SPAST, CUL3, ARHGAP6, MAP4, LTBP1, ELN, KIF1B, SYNE2, EML1, KIF26A, HDAC4, KIF2A, KCNAB2, ACTN1, MARK2, FERMT2, SRI, ACTB, PPP1R12B, FBLN1, NEBL, KIF22, CARMIL1, SCTR, CACNA1S, PKP1, KIF3C, AURKA, FKBP1A, NRP1, MYO9B, MISP, CEP170B, MYH9, NIN, MAPRE1, MAP1LC3A, TNNC2, MYOM1, PARP4, KATNAL1, CSPP1, JPH1, NDRG1, MYH14, DNAH11, DNAJB6, COBL, HSPB1, AKNA, AK1, SLAIN2, CORO1C, LMNB1, DBN1, CLIP4, SPTBN1, RMDN2, SLC1A4, WIPF1, KIF17, CAMSAP2, CASQ2, DNAH7, TRIM32, FKBP1B, MYOT, WIPF3, CALD1, KLC1, MACF1, RAC2, FLNC, MYO1B, PALLD, MYOD1, KIF1C, MTUS1, FGF13, CAMSAP1, COL5A1, TUBGCP2, CLIP1, SYNE1, GFAP, PDLIM4, MAP1B, NES, MYO18B, PSRC1, NAV1, MYBPC3, HOOK1, FHOD3, TPGS2, APC, ANXA1, CEP162, TTLL4, CAB39, BIN1, ODF2, CDK5RAP2, KIF12, KIF13A, KIF23, HAUS2, LRRC49, TTLL7, SHROOM3, PPP3CA, FBN2, TPM1, IQGAP1, KATNB1, BCAS3, DPP9, ADAMTS10, MYOM3, FLG, SCN1A, COL8A1, MYH15, IQGAP2, DCDC2, ARHGAP18, SHROOM2, TRIM55, JPH2, ALDOA, ANK3, NEK7, DST, TUBA3E, HAUS1, OBSCN, PDLIM3, TTN, SPAG17, WHAMM, CCT8, TIAM1, FBXO32, DNAH3, SYNJ1, SPTBN4, LMNA, SQSTM1, NEXN, XIRP2, PDLIM5, PBXIP1, COL6A3, CCT3, SHROOM1, KIF27, EML5, ANKRD2, CEP295, CEP57, JAM3, FBN1, CLMP, MYO1A, MAPRE2, TUBA1C, EVPL, MYL1, ROR2, RGS14, CDK1, TTLL6, KIF5B, BFSP2, SYNPO, MYOZ2, SYNPO2, EFEMP2, CNP, CSRP2, LMNB2, PDE4DIP, MAP6D1, FIGN, DNAH2, PDE4B, AHNAK2, ESPN, DYNC2H1, THSD4, PARVB, COL27A1, KRT35, SVIL, MYO5A, MFAP5, MYH6, PARVA, NRAP, PRC1, DMD, PTPN20, COL5A2, EML6, KIF4B, FMN1, MAP1LC3B2, KRTAP3-1, KRT39, KRT33B, MYZAP, KRTAP4-11, KRTAP4-12, RASSF5, KRTAP10-7, KRTAP5-5, NEFL, RCC2, CACNA1C* |
| Apical junction complex | 0.0072 | 42 | 154 | 1.6951 | *CYTH3, CTNNA1, RHOA, PRKCZ, ACTB, EPB41L4B, SORBS1, VAPA, CYTH1, CCND1, LIN7A, CLDN16, FRMD4B, STRN, TJP2, PARD6B, VASP, WNK4, NECTIN2, PATJ, APC, SHROOM3, USP53, SHROOM2, MTDH, LIN7C, ANK3, FRMD4A, CLDN8, SHROOM1, CLDN2, JAM3, CLMP, PLEKHA7, CLDN20, SYNPO, PDZD3, NECTIN3, CLDN6, MAGI2, NHS, MAGI1* |
| Microtubule | 0.0075 | 103 | 464 | 1.3797 | *DNAH9, SPAST, CUL3, MAP4, KIF1B, EML1, KIF26A, KIF2A, KCNAB2, KIF22, SCTR, KIF3C, AURKA, MISP, CEP170B, NIN, MAPRE1, MAP1LC3A, PARP4, KATNAL1, CSPP1, NDRG1, DNAH11, AKNA, SLAIN2, CLIP4, RMDN2, KIF17, CAMSAP2, DNAH7, KLC1, MACF1, KIF1C, MTUS1, FGF13, CAMSAP1, TUBGCP2, CLIP1, MAP1B, PSRC1, NAV1, HOOK1, TPGS2, APC, CEP162, TTLL4, ODF2, CDK5RAP2, KIF12, KIF13A, KIF23, HAUS2, LRRC49, TTLL7, SHROOM3, IQGAP1, KATNB1, BCAS3, DPP9, IQGAP2, DCDC2, ARHGAP18, SHROOM2, TRIM55, NEK7, DST, TUBA3E, HAUS1, SPAG17, WHAMM, CCT8, TIAM1, DNAH3, SYNJ1, PBXIP1, CCT3, SHROOM1, KIF27, EML5, CEP295, CEP57, CLMP, MAPRE2, TUBA1C, ROR2, RGS14, CDK1, TTLL6, KIF5B, CNP, PDE4DIP, MAP6D1, FIGN, DNAH2, DYNC2H1, SVIL, PRC1, PTPN20, EML6, KIF4B, MAP1LC3B2, RASSF5, RCC2* |
| Distal axon | 0.0075 | 74 | 313 | 1.4694 | *ALS2, RUFY3, USH2A, DTNBP1, RASGRF1, AP3D1, MYO9A, CNGB1, CRMP1, SRI, ACTB, NRP1, CBARP, NIN, MYH14, PTPRS, COBL, SLC1A1, ZPR1, HSPA8, DBN1, TANC1, ITGA4, AAK1, EPHA4, EXOC8, GOT1, TSHZ3, PREX1, PACSIN1, KLC1, PALLD, FGF13, OLFM1, EXOC4, MAP1B, NTS, HTR1B, IQGAP1, KATNB1, ADCYAP1, APP, ADCY10, SLC4A10, CPLX2, TENM2, TIAM2, NTRK2, EPS8, UTRN, TIAM1, DGKI, AUTS2, PPP1R9A, GDPD5, PINK1, SYNJ1, BRSK1, ADORA1, APBB2, GRIK2, KCNC2, PRKCB, PRRT2, KIF5B, BASP1, SLC17A8, EXOC3, TRAK1, ROR1, STMN3, MYO5A, UNC13B, NEFL* |
| Leading edge membrane | 0.0083 | 49 | 189 | 1.6114 | *CD44, USH2A, SYNE2, PSD, RHOA, FERMT2, ITGA8, AMPH, FERMT1, BMX, PIEZO1, DOCK8, KANK1, MPP2, SLC1A2, CORO1C, RIPOR2, WWC1, SPTBN1, WLS, PLCG1, PACSIN1, PSD4, VASP, MACF1, HIP1R, EPS8L1, INPP5K, APC, TLN1, ITGAV, TPM1, EGFR, PTPRJ, EPS8, ADAM17, FGD5, GRIA1, PSD3, TIAM1, ADORA1, ADGRV1, DLC1, KCNC2, ANTXR1, CFL1, PIP5K1C, PLCG2, PTPRK* |
| Cluster of actin-based cell projections | 0.0098 | 45 | 171 | 1.6356 | *USH1C, ABCC2, USH2A, LIMA1, MYO3B, CYBRD1, ACTN1, CDHR2, ATP8B1, SNX5, MYH9, HOMER2, TRPA1, MYH14, CUBN, CDH23, RIPOR2, CLIC5, GIPC1, VIL1, DOCK4, MYO1B, ENPEP, PTPRQ, DCDC2, ADD3, SLC7A11, EPS8, TMPRSS15, PLB1, ADGRV1, MYO1A, MYO7B, PDZD3, DAB1, FSCN2, ESPN, LCTL, MME, SLC22A5, SLC28A3, SLC22A12, SLC34A3, AQP1, MYO15B* |
| Microfibril | 0.0116 | 7 | 11 | 3.9552 | *LTBP1, FBN2, ADAMTS10, FBN1, EFEMP2, THSD4, MFAP5* |
| Brush border | 0.0122 | 33 | 116 | 1.7681 | *USH1C, ABCC2, LIMA1, CYBRD1, ACTN1, CDHR2, ATP8B1, SNX5, MYH9, MYH14, CUBN, GIPC1, VIL1, MYO1B, ENPEP, ADD3, SLC7A11, EPS8, TMPRSS15, PLB1, MYO1A, MYO7B, PDZD3, DAB1, ESPN, LCTL, MME, SLC22A5, SLC28A3, SLC22A12, SLC34A3, AQP1, MYO15B* |
| Cell projection membrane | 0.0144 | 83 | 368 | 1.4018 | *PROM1, ABCC2, CD44, USH2A, LIMA1, SYNE2, PSD, RHOA, CNGB1, CYBRD1, EVC, FERMT2, CDHR2, ITGA8, CEACAM1, ATP8B1, FERMT1, MAPRE1, BMX, PIEZO1, IQCE, DOCK8, KANK1, PKD2L1, CUBN, MPP2, SLC1A2, CORO1C, RIPOR2, WWC1, SPTBN1, WLS, BBS9, PLCG1, PACSIN1, PSD4, VASP, MACF1, MCHR1, HIP1R, EPS8L1, INPP5K, GUCY2D, PDE6A, APC, TLN1, ITGAV, TPM1, MYO10, EGFR, PTPRJ, ROM1, SLC7A11, EPS8, ADAM17, UTRN, ASAP1, FGD5, GRIA1, SLC26A2, PSD3, TIAM1, PKD1L1, DHRS3, ADORA1, PLB1, ADGRV1, DLC1, KCNC2, ARL13B, ANTXR1, CFL1, GPR157, KIRREL1, PIP5K1C, SLC22A5, SLC28A3, SLC22A12, PLCG2, SLC34A3, DMD, PRCD, AQP1* |
| Perisynaptic extracellular matrix | 0.0144 | 4 | 4 | 6.2153 | *TNC, PTPRZ1, TNR, ACAN* |
| Synapse-associated extracellular matrix | 0.0144 | 4 | 4 | 6.2153 | *TNC, PTPRZ1, TNR, ACAN* |
| Platelet alpha granule | 0.0171 | 29 | 100 | 1.8024 | *ITGA2B, IGF1, ACTN1, PDGFB, SERPINE1, VWF, PHACTR2, VEGFA, HRG, FN1, TGFB3, A1BG, PLG, VAMP7, SERPINE2, STXBP1, MMRN1, APP, LHFPL2, PCYOX1L, SERPING1, ALDOA, CD109, FGG, PROS1, TMSB4X, PECAM1, CFD, ACTN4* |
| Spectrin | 0.0193 | 6 | 9 | 4.1435 | *EPB41L2, SPTBN1, SPTBN4, SPTA1, PRKCB, SPTBN2* |
| Actin-based cell projection | 0.0233 | 56 | 234 | 1.4874 | *USH1C, PROM1, RUFY3, CD44, USH2A, SYNE2, HYAL2, OSBPL3, MYO3B, CDHR2, UBE2K, RAPGEF3, CEACAM1, ATP8B1, FAT1, AKR1B1, HOMER2, CDH23, RIPOR2, EPHA4, VAMP7, VASP, VIL1, DOCK4, MYO1B, FGF13, EXOC4, PDGFRA, ITGAV, FGD4, APP, MYO10, IQGAP2, TENM2, TIAM2, EPS8, FARP1, UTRN, ANGPT1, ENAH, SLC26A2, PPP1R9A, ADGRV1, INPPL1, JAM3, MYO1A, ANTXR1, NLGN1, MYO7B, CNP, WWOX, FSCN2, ESPN, MYO5A, DMD, STARD10* |
| Myosin complex | 0.0327 | 19 | 59 | 2.0015 | *MYO16, LIMCH1, MYO9A, MYO3B, MYO9B, MYH9, MYH14, MYO1B, MYO18B, CCDC102A, MYH15, MYO10, MYO1A, MYO5B, MYL1, MYO7B, MYO5A, MYH6, MYO15B* |
| Centrosome | 0.0354 | 134 | 658 | 1.2657 | *MAD1L1, ALS2, CDC27, DDX11, TACC3, CCDC88C, SPAST, RNF19A, CUL3, CNTLN, FNIP2, CROCC, KIF2A, CRMP1, WDR62, CDC14B, GSK3B, ERC1, PIBF1, TTC39A, RTRAF, AURKA, PPP2R3C, SORBS1, EFHC1, IFT74, MISP, CYTH4, IFT27, POLR3H, NIN, KIAA0586, MAPRE1, ARHGEF7, KATNAL1, NUP93, CSPP1, NDRG1, CCDC61, CDK6, AKNA, CCNJ, ANKRD26, B9D1, SLAIN2, CTSC, CEP85L, RAB23, SLC1A4, SSX2IP, CENPF, CAMSAP2, CNTRL, NEK9, IFT43, BBS9, STIL, TUBGCP2, CLIP1, SYNE1, VPS4A, PATJ, KCTD1, HOOK1, DZIP1, APC, CEP162, DYSF, ODF2, CDK5RAP2, KIF13A, NEK1, KIF23, HAUS2, CDKL2, BRCA2, KATNB1, SMAD4, MVB12A, DCDC2, HMBOX1, CHEK1, HNMT, HAUS1, AK5, CEP112, TSEN2, CCT8, RPGR, TBC1D31, EYA3, BRSK1, MAPKAPK2, CCDC141, PPP4R2, POC1A, RAPSN, CEP295, CEP57, PLEKHA7, DCTN5, PXK, AXIN2, CEP120, RGS14, CCDC8, CDK1, KIF5B, PDE4DIP, RPP25, HARBI1, PLAG1, IST1, LCK, CABCOCO1, FBXL7, CLECL1, PDE4B, HEPACAM2, BLOC1S2, SFI1, UVRAG, ITSN2, CEP290, TRAF3IP1, PTPN20, ZBED1, UBXN2B, DDX3X, CCDC187, ARHGEF10, PPP4R3B, SLC16A1, FLII* |
| Ruffle membrane | 0.0361 | 29 | 105 | 1.7166 | *PSD, RHOA, FERMT1, BMX, KANK1, CORO1C, WWC1, PLCG1, PACSIN1, PSD4, MACF1, HIP1R, EPS8L1, INPP5K, APC, TLN1, ITGAV, TPM1, EGFR, PTPRJ, EPS8, ADAM17, FGD5, PSD3, TIAM1, DLC1, CFL1, PIP5K1C, PLCG2* |
| Dendrite | 0.0446 | 136 | 674 | 1.2541 | *ALS2, RUFY3, SAMD4A, DTNBP1, KIF1B, PSD, RHOA, INPP5A, MARK2, TP63, SRI, MARK3, ITGA8, GSK3B, MTMR2, PALMD, NIN, TRIM9, ZMYND8, PLCB4, RBM3, NECAB2, HOMER2, PPP2R1A, COBL, SLC1A1, ABHD17B, MAPK8, BMPR1A, MPP2, HSPA8, DBN1, TANC1, MLPH, STRN, SLC1A4, EPHA4, WLS, KIF17, GIPC1, PREX1, STAU1, PCSK2, KIF1C, FGF13, APOE, ATXN10, HIP1R, PDLIM4, MAP1B, RARA, DNAJB1, DLG4, IL6ST, HTR1B, CAPRIN1, DOCK10, BIN1, ARRB1, TTLL7, SEPTIN11, ANXA3, PPP3CA, PPFIA2, ABHD13, MINK1, RPTOR, APP, AZIN2, ADCY10, CNIH3, SLC4A10, GLRA3, CPLX2, TENM2, NTRK2, HTR7, ANK3, SAV1, FARP1, ASAP1, GRIA1, ADCY8, TIAM1, NPTN, DGKI, PPP1R9A, RGS12, ADORA1, GRIK2, HCN1, KCNC2, CKB, PRRT2, ROR2, RGS14, PTK2, TMEM266, NLGN1, CTNND2, FRMPD4, KIF5B, GPHN, SYNPO, HCFC1, NECTIN3, SLC17A8, SHARPIN, KCNIP1, TRAK1, KCNB2, MX2, PDE4B, OSBP2, SORCS2, ANKS1B, NTF3, BCR, MAGI2, MME, LAMA2, NF1, STRN3, HTT, SIPA1L1, ERO1A, OPA1, ITSN1, ELFN1, SRGAP2, SRCIN1, ABR, APBA2, DRD4, CACNA1C, CLSTN3* |
| Growth cone | 0.0446 | 46 | 190 | 1.5047 | *ALS2, RUFY3, DTNBP1, RASGRF1, MYO9A, CRMP1, NRP1, CBARP, NIN, MYH14, PTPRS, COBL, ZPR1, DBN1, ITGA4, EPHA4, EXOC8, TSHZ3, PREX1, KLC1, PALLD, FGF13, OLFM1, EXOC4, MAP1B, IQGAP1, KATNB1, APP, ADCY10, TENM2, TIAM2, EPS8, UTRN, TIAM1, AUTS2, PPP1R9A, GDPD5, PINK1, APBB2, KIF5B, BASP1, EXOC3, TRAK1, STMN3, MYO5A, NEFL* |
| Sarcoplasm | 0.0466 | 23 | 79 | 1.8095 | *DTNBP1, SYNE2, SRI, MEF2C, FKBP1A, P3H2, TMEM38B, JPH1, CASQ2, FKBP1B, FABP3, ITPR2, FLNC, CALU, HAX1, CAMK2D, JPH2, ITPR1, ANK3, SPOCK1, CACNA2D1, CALR, ASPH* |
| Site of polarized growth | 0.0466 | 47 | 196 | 1.4904 | *ALS2, RUFY3, DTNBP1, RASGRF1, MYO9A, CRMP1, FRY, NRP1, CBARP, NIN, MYH14, PTPRS, COBL, ZPR1, DBN1, ITGA4, EPHA4, EXOC8, TSHZ3, PREX1, KLC1, PALLD, FGF13, OLFM1, EXOC4, MAP1B, IQGAP1, KATNB1, APP, ADCY10, TENM2, TIAM2, EPS8, UTRN, TIAM1, AUTS2, PPP1R9A, GDPD5, PINK1, APBB2, KIF5B, BASP1, EXOC3, TRAK1, STMN3, MYO5A, NEFL* |
| Platelet alpha granule lumen | 0.0466 | 21 | 70 | 1.8646 | *IGF1, ACTN1, PDGFB, SERPINE1, VWF, VEGFA, HRG, FN1, TGFB3, A1BG, PLG, MMRN1, APP, PCYOX1L, SERPING1, ALDOA, FGG, PROS1, TMSB4X, CFD, ACTN4* |
| Dendritic tree | 0.0469 | 136 | 676 | 1.2504 | *ALS2, RUFY3, SAMD4A, DTNBP1, KIF1B, PSD, RHOA, INPP5A, MARK2, TP63, SRI, MARK3, ITGA8, GSK3B, MTMR2, PALMD, NIN, TRIM9, ZMYND8, PLCB4, RBM3, NECAB2, HOMER2, PPP2R1A, COBL, SLC1A1, ABHD17B, MAPK8, BMPR1A, MPP2, HSPA8, DBN1, TANC1, MLPH, STRN, SLC1A4, EPHA4, WLS, KIF17, GIPC1, PREX1, STAU1, PCSK2, KIF1C, FGF13, APOE, ATXN10, HIP1R, PDLIM4, MAP1B, RARA, DNAJB1, DLG4, IL6ST, HTR1B, CAPRIN1, DOCK10, BIN1, ARRB1, TTLL7, SEPTIN11, ANXA3, PPP3CA, PPFIA2, ABHD13, MINK1, RPTOR, APP, AZIN2, ADCY10, CNIH3, SLC4A10, GLRA3, CPLX2, TENM2, NTRK2, HTR7, ANK3, SAV1, FARP1, ASAP1, GRIA1, ADCY8, TIAM1, NPTN, DGKI, PPP1R9A, RGS12, ADORA1, GRIK2, HCN1, KCNC2, CKB, PRRT2, ROR2, RGS14, PTK2, TMEM266, NLGN1, CTNND2, FRMPD4, KIF5B, GPHN, SYNPO, HCFC1, NECTIN3, SLC17A8, SHARPIN, KCNIP1, TRAK1, KCNB2, MX2, PDE4B, OSBP2, SORCS2, ANKS1B, NTF3, BCR, MAGI2, MME, LAMA2, NF1, STRN3, HTT, SIPA1L1, ERO1A, OPA1, ITSN1, ELFN1, SRGAP2, SRCIN1, ABR, APBA2, DRD4, CACNA1C, CLSTN3* |
| Presynapse | 0.0477 | 109 | 527 | 1.2855 | *USH2A, DTNBP1, KIF1B, SNCAIP, AP3D1, SYT1, GNB5, CNGB1, SRI, ACTB, GPC4, AMPH, SYNJ2, RIMS1, CADPS2, ERC1, MTMR2, CBARP, TRIM9, PTPRS, SLC1A1, DNMBP, FBXL20, HSPA8, SLC1A2, PPFIBP1, LIN7A, TANC1, AAK1, EPHA4, PCDH17, GOT1, GIPC1, VAMP7, PACSIN1, HIP1, SLC6A11, DDC, DLG4, NTS, CNTN6, HTR1B, BIN1, STXBP1, KIAA1109, PPFIA2, STON2, MCTP2, ADCYAP1, APP, PPFIA4, SLC4A10, IQSEC1, CPLX2, NTRK2, LIN7C, WNT3A, GRIA1, ADCY8, NPTN, ATP2B2, NRG1, DGKI, SYNJ1, CTBP1, BRSK1, PDLIM5, ADORA1, CADPS, SLC9B2, EGFLAM, SEPTIN8, GRIK2, KCNC2, PPFIBP2, PRKCB, PRRT2, NLGN1, YWHAG, CLCN5, SYT12, SPTBN2, CTBP2, MCTP1, ERBB4, SLC17A8, EXOC3, MX2, PDE4B, SEPTIN5, PRKN, ROR1, NTF3, SYN3, PIP5K1C, ZNRF1, MME, SLC6A17, HTT, ITSN2, UNC13B, DMD, ITSN1, VDAC1, STON1, SEC22B, SRCIN1, APBA2, NEFL* |
| Neuromuscular junction | 0.0480 | 22 | 75 | 1.8231 | *MUSK, HDAC4, DLGAP4, MYH9, CHRNE, EPHA4, PDZRN3, LAMA5, DLG4, SERPINE2, APP, DLG2, ANK3, SPOCK1, UTRN, NRG1, PPP1R9A, RAPSN, LAMA2, UNC13B, NEFL, CHRNB1* |

**S11 Table**. **WGS results, list of genes with high impact, missense, and regulatory elements variants attributed to the selected biological pathways.** Variants identified in control samples and variants with MAF>0.1 in the gnomAD database were excluded.

| Pathway module, Pathway | Genes with variants |
| --- | --- |
| Cytokine signaling, Interferon alpha/beta | ***HLA-A****,* ***MX2****,* ***XAF1****, IRF1^†^, IFITM3^†^, IRF2, IFI35, ISG20, IFI27, TYK2, OAS1* |
| Cytokine signaling, Interferon gamma | ***HLA-A****,* ***ICAM1****,* ***GBP7,*** *IRF1^†^, IFITM3^†^, GBP5, GBP1, MT2A, PTPN2, PML, OAS1, IRF2, TRIM31, SUMO1, CD44, TRIM35, CAMK2D, TRIM5, NCAM1, TRIM2, IFNGR2* |
| Cytokine signaling, Interleukin-1 | ***PTPN14, SQSTM1****,* ***PSMD3****, IL1R1^†^, PTPN12^†^, PELI2, TAB3, PTPN20, TAB1, IL1RAP, PTPN2, APP, SIGIRR, IL1RL1, UBE2N, PSMD1, UBE2V1, TRAF6, MAPK8, STAT3, PSMB1, PSMA5, PSMC6* |
| Cytokine signaling, Interleukin-6 | ***CNTFR****, OSM, TYK2, IL6ST, OSMR, STAT3* |
| Innate immune system, Neutrophil degradation | ***PECAM1****^*†^,* ***ATPSCKMT****,* ***HPSE****,* ***A1BG****,* ***HLA-A****,* ***TOM1****,* ***GALNS****,* ***PFKL****,* ***CEP290****,* ***GUSB****,* ***PIEZO1****,* ***EPO****,* ***PSMD3****,* ***LILRA6****,* ***ITGAD****,* ***EPX****,* ***IQGAP2****,* ***ATAD3A****, ANO6^†^, TMC6^†^, GDI2^†^, HSPA6^†^, S100Z^†^, FCGR3B^†^, RAB7B^†^, XRCC5, KCMF1, FCGR3B, GOLGA7, ATP8B4, ATP6V0C, ARSB, LPCAT1, PSMD1, CCT8, MME, TCN1, CD59, SELL, GAA, PRC1, QPCT, CPPED1, RAB18, IQGAP1, TAB1, CPNE1, PTPRJ, VAPA, BRI3, S100Z, DLC1, HVCN1, GALNS, VNN1, GLA, FPR1, RNF38, RAP1B, DERA, SDCBP, PTPRC, RAP1A, IQGAP2, PLD1, ADA2, AMPD3, ATP11A, HGSNAT, CTSC, SH3GLB2, CEACAM6, ALDOA, RHOG, TMC6, IST1, CEACAM1, CEACAM3, PSMB1, LILRA6, RAB31, RHOA, PKP1, PADI2, CAB39, CYFIP1, CFD, STK10, GLIPR1, CD44, DDX3X, DOCK2, RAB7B, SLC27A2, ITGAV, LTF, PYGL, PAFAH1B2, KCNAB2, GDI2, PECAM1, OSCAR, PSMA5, RAB44, ITGAD, LRRC43, CKAP4* |
| Innate immune system, Complement cascade | ***IGHV3-48****,* ***IGLV2-8****,* ***C9****,* ***IGLV8-61****, C1S^†^, PROS1^†^, SERPING1, CFD, IGHV3-30, SVEP1, CD81, IGKV2-29, CFH, CD59, CPN2, VTN, C6, C8B, C8A* |
| Adaptive Immune System, Class I MHC antigen processing&presentation | ***HLA-A****,* ***UBE2Q2****,* ***TOM1****,* ***FBXO27****,* ***PSMD3****,* ***RNF213****,* ***KBTBD13****,* ***SH3RF1****,* ***CDRT1****,* ***ANAPC10****, NEDD4L^†^, SMURF1^†^, S100Z^†^, ZNRF1^†^, SPSB1^†^, ASB2^†^, PJA2^†^, ANAPC5, MRC2, TRIM9, RNF126, TRIM41, FBXL7, ANAPC1, SEC22B, MIB2, PSMD1, GLMN, SEC61G, HECW2, FGG, FBXL20, SEC61A1, SH3RF1, CUL3, UBE3B, UBA6, UBE3D, UBE3C, RNF19A, MKRN1, CDC27, HERC4, TRIM32, LNX1, TRIM36, UBE2N, TRIM37, CALR, TRIM4, UBOX5, PSMC6, PRKN, FBXO32, FBXO15, SMURF2, HECTD2,, FBXO17, UBE2H, UBE2K, CDRT1, RNF220, KLHL3, PSMB1, LMO7, TFAM, CBLB, NCF4, RNF217, PXK, RNF213, ITGB5, ITGAV, UBE2V2, UBE2V1, FBXW9, FBXW7, UNK, ANAPC13, PSMA5, PATJ, NPEPPS, ABO* |
| Signal transduction, RHO GTPase Effectors (inc. Activated PKN1 stimulates transcription of AR regulated KLK2 and KLK3) | ***ERCC6L****,* ***CLIP1****,* ***PRKCB****,* ***TUBA3E****,* ***IQGAP2****, S100Z^†^, PPP2R1A^†^, KIF5B^†^, RCC2^†^, MAPK11^†^, STK4^†^, MYLK^†^, MYH9, MYH14, H3C2, H2BC6, H2AC6, KDM4C, PPP1R12B, H3-3B, YWHAG, H3-3A, YWHAZ, RHOB, RHOA* |
| Signal transduction, Signaling by Nuclear Receptors (ESR-mediated) | ***MED1****,* ***NCOA1****,****CCNT1****, EMB, NOS3, H2AC6, CREB1, STAG1, KDM4B, ZDHHC7, CCND1, GNAT3, ESR1, PTK2, ESR2, GNB5, FOSB, PIK3R2, STRN, PIK3R1, ERBB4, TNRC6C, KRAS, TNRC6B, H3-3B, H3-3A, CXCL12, GNG2, CREBBP, YY1, CDK9, EGFR, RUNX1, AXIN1, H3C2, H2BC6, POLR2B, JUN, POLR2J, AKT3, NCOA3, GTF2F2, GTF2F1, HSPB1, PIK3CA, HDAC1, PXK, KANK1, PRKCZ* |
| Signal transduction, Signaling by Receptor Tyrosine Kinases (Signaling by EGFR) | ***ARHGEF7****,* ***LIG1****, PTPN12^†^, LRIG1^†^, EPS15L1, CSK, EGFR, PTPRK, PIK3R1, PLCG1, PIK3CA, ARHGEF7, KRAS, ADAM17, ADAM12, GAB1* |
| Signal transduction, Signaling by TGFB family members | ***TGIF1****,* ***CCNT1****, NEDD4L^†^, SERPINE1^†^, SMURF1^†^, FST^†^, CDKN2B, INHBA, EMB, SMOX, SMURF2, WWTR1, FKBP1B, STAG1, E2F5, ITGA8, TGFB3, SUB1, FKBP1A, USP15, LTBP1, RHOA, TFAM, HDAC1, NCOR2, ITGB5, CCNT2, ITGAV, FBN1, BMPR1A, FPR1, ACVR1B, SMAD4, SMAD1, CDK6, PRKCZ, SMAD6, CDK9* |
| Signal transduction, Signaling by WNT | ***LGR5****,* ***ROR2****,* ***PRKCB****,* ***PSMD3****,* ***HECW1****,* ***CCDC88C****, NFATC1^†^, SMURF1^†^, PPP2R1A^†^, LRP5^†^, RAC2, CSNK1E, EMB, WNT3A, H2AC6, PRKCB, FZD1, VANGL2, WLS, PSMD1, HECW1, YWHAZ, TCF7L2, GNB5, ITPR1, ITPR2, LGR4, CUL3, TCF4, LGR5, APC, TNRC6C, KRAS, PRKG1, TNRC6B, H3-3B, GSK3B, H3-3A, GNG2, PPP3CA, BCL9, CREBBP, PSMC6, AXIN1, H3C2, AXIN2, SMURF2, H2BC6, PDE6A, WNT5B, DACT1, PSMB1, TLE4, RHOA, CTBP1, CTBP2, ROR1, LRTM1, HDAC1, PLCB1, PPP2R5C, RSPO2, NLK, DAAM1, PSMA5* |
| Cell-cell communication, Cell-cell junction organization | ***LAMC2****^*†^,* ***COL17A1****^*†^,* ***SDK1****^*†^,* ***NECTIN2****,* ***SDK2****, CLDN6^†^, FLNC, CLDN8, LIMS1, LIMS2, PARVA, CLDN2, PARVB, MEGF11, DST, VASP, PARD6B, ACTB, CDH6, LAMC2, CLDN16, FERMT2, CTNNA1, NECTIN3, CLDN20, ACTN1, PATJ* |
| Extracellular matrix organization, Integrin cell surface interaction | ***PECAM1****^*†^,* ***TNC****,* ***FN1****,* ***COL23A1****,* ***VWF****,* ***COL5A1****,* ***ITGAD****,* ***ICAM1****, ITGA4^†^, COL5A2^†^, COL8A1, COL6A3, ITGAD, COL13A1, ITGA11, COL5A1, CD44, ITGB5, VTN, ITGB7, ITGAV, FBN1, JAM3, ITGA8, TNC, ITGA2B, HSPG2, FGG* |
| Extracellular matrix organization, Collagen formation | ***LAMC2****,* ***P3H1****,* ***COL23A1****,* ***COL5A1****,* ***COL17A1****,* ***COLGALT1****, COL27A1^†^, ADAMTS2^†^, P4HA1^†^, COL5A2^†^, COL21A1, COL8A1, COL6A3, COL25A1, COLGALT2, COL5A1, P3H2, LOXL4, P3H1, MEGF11, DST, PXDN, COL17A1, LOXL2, LAMC2, PLOD2, COL28A1, COL14A1, LOX, COL13A1, TLL2, COL12A1* |
| Extracellular matrix organization, ECM proteoglycans | ***TNC****,* ***FN1****,* ***LAMB1****,* ***COL5A1****,* ***LAMA5****,* ***ITGAD****, TNR^†^, COL5A2^†^, SERPINE1^†^, COL6A3, MUSK, COL5A1, LRP10, ITGA8, TGFB3, ITGA2B, ACAN, DCN, LAMC1, LAMA1, ASPN, LAMA2, APP, LAMA5, PTPRS, ITGB5, ITGAV, MATN1, TNC, NCAM1, VTN, ITGAD, TNXB, HSPG2* |
| Extracellular matrix organization, Degradation of ECM | ***FN1****,* ***LAMB1****,* ***LAMC2****,* ***COL23A1****,* ***COL5A1****,* ***COL17A1****,* ***LAMA5****,* ***CAPN9****,* ***CAPN12****,* ***NID1****, COL5A2^†^, COL8A1, COL6A3, COL25A1, COL5A1, GPR37, CAST, MEGF11, MME, SUB1, ACAN, COL17A1, DCN, HTRA1, LAMC1, LAMC2, COL14A1, COL13A1, TLL2, COL12A1, MMP17, CD44, FBN2, LAMA5, ADAMTS9, ADAM17, FBN1, PLG, ELN, CAPN13, NID1, CAPN15, HSPG2* |
| Programmed cell death, Apoptosis | ***TJP2****^*^,* ***PSMD3****, CARD8^†^, LMNA, SH3GLB2, TP63, ACIN1, PSMD1, MAGED1, SATB1, MAPK8, AKT3, PSMB1, BMX, YWHAZ, PTK2, LMNB1, PKP1, APC, DLC1, YWHAG, CFLAR, OPA1, STAT3, CYCS, PSMA5, PSMC6, STK3* |
| DNA repair, Base excision/Double-strand breaks repair/Mismatch repair | ***NEIL3****^*†^,* ***MED1****,* ***LIG1****,* ***PNKP****,* ***TDG****,* ***RBBP8****,* ***CHEK1****,* ***BRCA2****,* ***TOP3A****,* ***EYA4****, SPIDR^†^, RTEL1^†^, H2BC6, H2AC6, GAA, CDC27, OGG1, TERF2, DUSP8, NBN, XRCC5, EXO1, ERCC1, KDM4B, BRIP1, HUS1, MAPK8, EYA3, RAD51B, PALB2, BAZ1B, LIG4, SUMO2, PPP4R2, PRKDC, SUMO1, UBE2V2, UBE2N, CDK1, MSH2, MSH3, PMS1* |
| Protein modifications, Ubiqutination | ***UBE2Q2****,* ***HLA-A****, PEX5^†^, FAM, DERL1, UBA6, WAC, H2BC6, SHPRH, PRKDC, RNF144A, UBE2H, UBE2K, UBE2V2, UBE2N, USP7* |
| Protein modifications, O-linked Glycosylation | ***MUC5AC****^†^,* ***MUC17****,* ***MUC16****,* ***MUC5B****,* ***MUC4****, ADAMTS10^†^, SEMA5A^†^, ADAMTS12^†^, ADAMTS2^†^, ADAMTS6^†^, GALNT1, ADAMTSL2, ADAMTS19, LARGE1, ST3GAL4, GALNTL6, GALNT15, GALNT18, GALNT12, C1GALT1C1, GALNT11, THSD7B, B3GLCT, SEMA5B, ADAMTS9, ADAMTS7, ST3GAL3, GCNT4, B3GNT7, B3GNT2, ADAMTSL1, POMGNT2, B3GNT8, THSD4* |
| Transcription, RNA polymerase II transcription | ***UCMA****^†^,* ***RBBP8****,* ***FANCC****,* ***ZNF77****,* ***TOP3A****,* ***PRKCB****,* ***ZNF442****,* ***TGIF1****,* ***WDR5B****,* ***TSC2****,* ***PSMD3****,* ***MED1****,* ***CPNE9****,* ***SMYD3****,* ***CCNT1****,* ***CHEK1****,* ***COX16****,* ***ZNF597****,* ***MAML2****,* ***HRG****,* ***CRYBA4****,* ***ZFP30****,* ***TFAP2E****,* ***ZNF138****,* ***ANAPC10****, ZNF705A, PIP4K2A, SERPINE1, SMURF1, SST, ITGA4, STK11, MAML3, NEDD4L, ZNF605, PPP2R1A, ZNF514, OMD, MAPK11, MED14, GLS* |
| Cell cycle, Mitosis/Cell Cycle Checkpoints | ***RBBP8****,* ***CHEK1****,* ***CLIP1****,* ***TOP3A****,* ***PSMD3****,* ***ERCC6L****,* ***NUP42****,* ***PRKCB****,* ***SFI1****,* ***CEP290****,* ***LPIN3****,* ***TUBA3E****,* ***CNTRL****,* ***POM121C****,* ***LIG1****,* ***HAUS1****,* ***SMC2****,* ***CDK11B****,* ***ANAPC10****, PPP2R1A^†^, RCC2^†^, PPP2R3B^†^, NEK7^†^, PRIM2^†^, SPAST^†^, AKT3, ANAPC1, ANAPC5, AURKA, BRIP1, BUB1B, CABLES1, CCND1, CCND3, CDC27, CDK1, CDK5RAP2, CDK6, CDKN2B, CDKN2C, CDKN2D, CENPF, CEP57, CHMP4B, CSNK1E, CYB561D1, DLC1, E2F3, E2F5, EMB, EXO1, FBXL7, FOSB, GAA, GSK3B, H2AC6, H2BC6, H3-3A, H3-3B, H3C2, HAUS2, HDAC1, HUS1, IST1, KIF23, KIF2A, LMNA, LMNB1, MAD1L1, MAPRE1, MCM4, MCM5, MPP2, NBN, NCAPH, NEK9, NIPBL, NSL1, NUP93, ODF2, PHF20, PPP1R12B, PPP2R5C, PSMA5, PSMB1, PSMC6, PSMD1, PTTG1, RAE1, SEH1L, STAG1, SUMO1, TFAM, TUBA1C, TUBGCP2, UBE2N, UBE2V2, VPS4A, YWHAG, YWHAZ* |
| Vesicle-mediated transport, Membrane trafficking, Clathrin-mediated endocytosis | ***HIP1R****,* ***APOB****,* ***PACSIN1****,* ***GAPVD1****, AAK1^†^, ADRB2^†^, SNX18^†^, EPS15L1, LDLR, PIP5K1C, SYNJ2, AGTR1, TACR1, SYNJ1, SYT1, TGOLN2, VAMP7, EMILIN2, EPN2, ACTB, FNBP1L, STON1, STON2, ARRB1, ITSN1, HIP1, ITSN2, AMPH, BIN1, SGIP1, EGFR* |
| Developmental biology, Keratinization | ***EVPL****^*^,* ***PCSK6****^*^,* ***KRTAP10-7****,* ***KRT35****,* ***FLG****,* ***KRT33B****, PKP3, KRTAP5-5, PKP4, PKP1, PI3, KRTAP4-11, KRTAP3-1, LIPM, SPRR1B, RPTN, KAZN, KRT39, SUB1* |
| Cellular responses to stimuli, Cellular responses to stress (Cellular Secenscence, Unfolded Protein Response) | ***MINK1****^*^,* ***ANAPC10****,* ***ACADVL****,* ***TLN1****, MAPK11^†^, BAALC, SSR1, CREB3L1, DDX11, NFYC, LMNA, EXTL2, CALR, CTDSP2, SCP2, PARN, PDIA5, CXCL8, EVPL, PCSK6, HMGA2, CDKN2B, H3C2, CBX2, E2F3, ANAPC5, NBN, H2BC6, H2AC6, MAPKAPK3, MAPKAPK2, JUN, MAP4K4, VENTX, ASF1A, ANAPC1, RPS6KA1, MAPK8, MAPK9, PHC2, TERF2, BMI1, PHC3, LMNB1, TFAM, EHMT1, EZH2, CDC27, TNRC6C, TNRC6B, H3-3B, H3-3A, STAT3, CDK1, CDKN2D, CDKN2C, ETS1, CDK6* |
| Chromatin organization, Chromatin modifying enzymes (Methylation) | ***WDR5B****,* ***KDM2D****,* ***SMYD3****, PRDM16^†^, KDM7A^†^, H3C2, KDM4C, KDM4B, KDM2B, SMYD3, EHMT1, WDR5, SETD7, DOT1L, SETD3, EZH2, CCND1, H2AC6, SMARCA2, ARID1B* |

**S12 Table. The count of the identified deletions located in previously published KTCN loci.**

|  |  | Number of deletions in KTCN loci | | | | | |
| --- | --- | --- | --- | --- | --- | --- | --- |
| KTCN loci | | KTCN_10 | KTCN_13 | KTCN_18 | KTCN_30 | M_5 | M_6 |
| 2q | 2:91,800,001 | 195 | 208 | 201 | 201 | 205 | 205 |
| 3p14.3 | 3:54,63  2,124-54,639,857 | 0 | 0 | 0 | 0 | 0 | 0 |
| 4q | 4:51,800,001 | 216 | 206 | 213 | 212 | 216 | 203 |
| 5p | 5:1-48,800,000 | 97 | 84 | 107 | 112 | 77 | 86 |
| 5q14.1-q21.1 | 5:77,600,001:98,900,001 | 26 | 22 | 32 | 26 | 32 | 31 |
| 5q35.2 | 5:173,300,001-177,100,000 | 5 | 6 | 5 | 5 | 7 | 4 |
| 9p | 9:1-43,000,000 | 53 | 52 | 61 | 57 | 57 | 55 |
| 11p | 11:1-51,000,000 | 80 | 81 | 103 | 94 | 89 | 89 |
| 12p | 12:1-33,200,000 | 62 | 59 | 65 | 54 | 72 | 59 |
| 13q32.3 | 13:98,916,905-98,981,997 | 1 | 0 | 0 | 0 | 1 | 1 |
| 14q | 14:18,200,001-107,043,718 | 111 | 120 | 134 | 139 | 121 | 120 |
| 15q22.33-24.2 | 15:66,900,001-76,300,000 | 15 | 16 | 13 | 17 | 13 | 16 |
| 16q22.3-q23.1 | 16:72,800,001-79,200,000 | 9 | 9 | 8 | 8 | 7 | 7 |
| 17p13 | 17:1-10,800,000 | 32 | 29 | 32 | 30 | 32 | 35 |
| 17q | 17:27,400,001 | 90 | 86 | 99 | 92 | 102 | 94 |
| 20p13 | 20:1-5,100,000 | 16 | 10 | 11 | 11 | 10 | 13 |
| 20q12 | 20:39,000,001-43,100,000 | 1 | 2 | 2 | 2 | 5 | 5 |

**S13 Table. The results of pathway enrichment analysis of 722 genes previously recognized as differentially expressed (DEGs) in full-thickness KTCN corneas and re-recognized as containing variants in regulatory elements (REs) localized in close proximity.** The Reactome database was chosen for definition of pathways.

| Pathway | Enrichment FDR | nGenes | Pathway Genes | Fold Enrichment | Genes |
| --- | --- | --- | --- | --- | --- |
| Extracellular matrix organization | >0.0001 | 54 | 418 | 3.3067 | *ITGA2B, CD44, TNC, ELN, LAMC2, ACTN1, FBLN1, ITGA8, CEACAM1, ITGB5, CEACAM6, P3H2, LAMA1, SERPINE1, ASPN, COL12A1, LOX, ITGA4, EFEMP1, NID1, P3H1, TGFB3, PLG, P4HA1, COL21A1, PXDN, COL5A1, LOXL2, ADAM19, ITGA11, EMILIN1, LOXL4, ITGAV, FBN2, HSPG2, ADAM12, PLOD2, ACAN, CAPN13, COL6A3, FBLN2, ADAMTS9, JAM3, FBN1, SDC2, COL14A1, LAMA2, COL27A1, COL13A1, MFAP5, MMP17, COL5A2, COL28A1, PECAM1* |
| Elastic fibre formation | >0.0001 | 15 | 59 | 6.5075 | *ELN, FBLN1, ITGA8, ITGB5, LOX, EFEMP1, TGFB3, LOXL2, EMILIN1, LOXL4, ITGAV, FBN2, FBLN2, FBN1, MFAP5* |
| Collagen formation | >0.0001 | 18 | 90 | 5.1192 | *LAMC2, P3H2, COL12A1, LOX, P3H1, P4HA1, COL21A1, PXDN, COL5A1, LOXL2, LOXL4, PLOD2, COL6A3, COL14A1, COL27A1, COL13A1, COL5A2, COL28A1* |
| Molecules associated with elastic fibres | 0.0001 | 12 | 52 | 5.9068 | *ELN, FBLN1, ITGA8, ITGB5, EFEMP1, TGFB3, EMILIN1, ITGAV, FBN2, FBLN2, FBN1, MFAP5* |
| Collagen biosynthesis and modifying enzymes | 0.0003 | 13 | 67 | 4.9664 | *P3H2, COL12A1, P3H1, P4HA1, COL21A1, COL5A1, PLOD2, COL6A3, COL14A1, COL27A1, COL13A1, COL5A2, COL28A1* |
| ECM proteoglycans | 0.0003 | 15 | 90 | 4.2660 | *ITGA2B, TNC, ITGA8, ITGB5, LAMA1, SERPINE1, ASPN, TGFB3, COL5A1, ITGAV, HSPG2, ACAN, COL6A3, LAMA2, COL5A2* |
| Hemostasis | 0.0003 | 55 | 738 | 1.9076 | *ITGA2B, IGF1, CD44, ACTN1, ACTB, RAPGEF3, CEACAM1, KIF3C, CEACAM6, RAPGEF4, SERPINE1, VEGFA, ITGA4, F3, TGFB3, PLG, ABCC4, IRF1, RAC2, DOCK4, CALU, VAV3, CABLES1, DOCK2, DOCK10, SERPINE2, DGKB, TRPC6, ITGAV, LHFPL2, SERPING1, DOCK1, ANGPT1, SLC7A7, ATP2B2, DGKI, ANXA5, JAM3, SDC2, PSG6, CEACAM3, RHOG, LCK, PROS1, PRKG1, GNG2, SELL, PLCG2, PSG5, PSG7, PSG1, PSG11, PSG4, SELENOP, PECAM1* |
| Integrin cell surface interactions | 0.0006 | 16 | 109 | 3.7572 | *ITGA2B, CD44, TNC, ITGA8, ITGB5, ITGA4, COL5A1, ITGA11, ITGAV, HSPG2, COL6A3, JAM3, FBN1, COL13A1, COL5A2, PECAM1* |
| Assembly of collagen fibrils and other multimeric structures | 0.0023 | 11 | 61 | 4.6157 | *LAMC2, COL12A1, LOX, PXDN, COL5A1, LOXL2, LOXL4, COL6A3, COL14A1, COL27A1, COL5A2* |
| Rho GTPase cycle | 0.0031 | 17 | 140 | 3.1081 | *ARHGAP31, DEPDC1B, TRIO, ARHGAP6, PREX1, RAC2, CHN1, STARD13, VAV3, ARHGAP24, FGD4, ARHGAP26, ARHGEF3, TAGAP, DLC1, RHOH, RHOG* |
| Collagen chain trimerization | 0.0036 | 9 | 44 | 5.2356 | *COL12A1, COL21A1, COL5A1, COL6A3, COL14A1, COL27A1, COL13A1, COL5A2, COL28A1* |
| Cell-extracellular matrix interactions | 0.0036 | 6 | 18 | 8.5321 | *ACTN1, LIMS2, FERMT2, ACTB, FLNC, PARVB* |
| Cell surface interactions at the vascular wall | 0.0062 | 20 | 198 | 2.5855 | *CD44, CEACAM1, CEACAM6, ITGA4, ITGAV, ANGPT1, SLC7A7, JAM3, SDC2, PSG6, CEACAM3, LCK, PROS1, SELL, PSG5, PSG7, PSG1, PSG11, PSG4, PECAM1* |
| Defective B3GALTL causes Peters-plus syndrome PpS | 0.0062 | 8 | 38 | 5.3887 | *ADAMTS6, ADAMTS7, ADAMTS10, ADAMTS9, ADAMTSL1, B3GLCT, THSD4, ADAMTSL2* |
| Platelet activation signaling and aggregation | 0.0062 | 26 | 295 | 2.2559 | *ITGA2B, IGF1, ACTN1, RAPGEF3, RAPGEF4, SERPINE1, VEGFA, TGFB3, PLG, ABCC4, RAC2, CALU, VAV3, DGKB, TRPC6, LHFPL2, SERPING1, DGKI, ANXA5, RHOG, LCK, PROS1, GNG2, PLCG2, SELENOP, PECAM1* |
| O-glycosylation of TSR domain-containing proteins | 0.0067 | 8 | 39 | 5.2505 | *ADAMTS6, ADAMTS7, ADAMTS10, ADAMTS9, ADAMTSL1, B3GLCT, THSD4, ADAMTSL2* |
| Non-integrin membrane-ECM interactions | 0.0067 | 11 | 73 | 3.8570 | *TNC, LAMC2, ACTN1, ITGB5, LAMA1, COL5A1, ITGAV, HSPG2, SDC2, LAMA2, COL5A2* |
| Platelet degranulation | 0.0076 | 15 | 128 | 2.9996 | *ITGA2B, IGF1, ACTN1, SERPINE1, VEGFA, TGFB3, PLG, ABCC4, CALU, LHFPL2, SERPING1, ANXA5, PROS1, SELENOP, PECAM1* |
| Response to elevated platelet cytosolic Ca2+ | 0.0110 | 15 | 133 | 2.8868 | *ITGA2B, IGF1, ACTN1, SERPINE1, VEGFA, TGFB3, PLG, ABCC4, CALU, LHFPL2, SERPING1, ANXA5, PROS1, SELENOP, PECAM1* |
| Syndecan interactions | 0.0142 | 7 | 34 | 5.2698 | *TNC, ACTN1, ITGB5, COL5A1, ITGAV, SDC2, COL5A2* |
| Regulation of Insulin-like Growth Factor IGF transport and uptake by Insulin-like Growth Factor Binding Proteins IGFBPs | 0.0159 | 14 | 124 | 2.8899 | *IGF1, TNC, FAM20A, STC2, EVA1A, PLG, CALU, APOE, IGFBP4, PRSS23, MELTF, FBN1, PCSK9, SDC2* |
| Degradation of the extracellular matrix | 0.0183 | 18 | 188 | 2.4507 | *CD44, ELN, LAMC2, COL12A1, NID1, PLG, COL5A1, FBN2, HSPG2, ACAN, CAPN13, COL6A3, ADAMTS9, FBN1, COL14A1, COL13A1, MMP17, COL5A2* |
| Post-translational protein phosphorylation | 0.0387 | 12 | 107 | 2.8706 | *TNC, FAM20A, STC2, EVA1A, CALU, APOE, IGFBP4, PRSS23, MELTF, FBN1, PCSK9, SDC2* |
| MET activates PTK2 signaling | 0.0387 | 6 | 30 | 5.1192 | *LAMC2, LAMA1, COL5A1, LAMA2, COL27A1, COL5A2* |
| Smooth Muscle Contraction | 0.0435 | 7 | 42 | 4.2660 | *MYLK, ITGB5, SORBS3, CALD1, ANXA1, TPM1, ANXA6* |
| NCAM1 interactions | 0.0483 | 7 | 43 | 4.1668 | *CACNA1I, COL5A1, ST8SIA2, COL6A3, GFRA2, CACNA1H, COL5A2* |

**S14 Table. STROBE Statement—checklist.**

STROBE Statement—checklist of items that should be included in reports of observational studies

|  | Item No | Recommendation |
| --- | --- | --- |
| **Title and abstract** | 1 | (*a*) Indicate the study’s design with a commonly used term in the title or the abstract  Indicated in the Abstract on pages 1-2. |
|  |  | (*b*) Provide in the abstract an informative and balanced summary of what was done and what was found  Provided in the Abstract on pages 1-2. |
| Introduction | | |
| Background/rationale | 2 | Explain the scientific background and rationale for the investigation being reported  Included in the Introduction on pages 3-4. |
| Objectives | 3 | State specific objectives, including any prespecified hypotheses  Stated in the Introduction on page 4. |
| Methods | | |
| Study design | 4 | Present key elements of study design early in the paper  Included in the Materials and Methods on pages 4-6 and presented in Figure 1 and S1 Figure. |
| Setting | 5 | Describe the setting, locations, and relevant dates, including periods of recruitment, exposure, follow-up, and data collection  Included in the Materials and Methods on pages 4-6 and in Supplementary Materials on pages 1-3. |
| Participants | 6 | (*a*) *Cohort study*—Give the eligibility criteria, and the sources and methods of selection of participants. Describe methods of follow-up  *Case-control study*—Give the eligibility criteria, and the sources and methods of case ascertainment and control selection. Give the rationale for the choice of cases and controls  *Cross-sectional study*—Give the eligibility criteria, and the sources and methods of selection of participants  Included in the Materials and Methods on page 5-6 and in Supplementary Materials on page 1-3. |
|  |  | (*b*) *Cohort study*—For matched studies, give matching criteria and number of exposed and unexposed  *Case-control study*—For matched studies, give matching criteria and the number of controls per case  Not applicable. |
| Variables | 7 | Clearly define all outcomes, exposures, predictors, potential confounders, and effect modifiers. Give diagnostic criteria, if applicable  Included in the Materials and Methods on pages 4-6 and in Supplementary Materials on pages 1-2. Diagnostic criteria are described in Supplementary Materials on page 1. |
| Data sources/ measurement | 8* | For each variable of interest, give sources of data and details of methods of assessment (measurement). Describe comparability of assessment methods if there is more than one group  Included in the Materials and Methods on pages 4-6 and in Supplementary Materials on pages 1-3. |
| Bias | 9 | Describe any efforts to address potential sources of bias  Included in the Supplementary Materials on page 2 and in the Discussion on pages 10-11. |
| Study size | 10 | Explain how the study size was arrived at  Included Supplementary Materials on page 2 and in the Discussion on page 11. |
| Quantitative variables | 11 | Explain how quantitative variables were handled in the analyses. If applicable, describe which groupings were chosen and why  Included in the Materials and Methods on pages 4-6 and in Supplementary Materials on pages 1-3. |
| Statistical methods | 12 | (*a*) Describe all statistical methods, including those used to control for confounding  Included in the Materials and Methods on pages 5-6 and in Supplementary Materials on pages 2-3. |
|  |  | (*b*) Describe any methods used to examine subgroups and interactions  Not applicable. |
|  |  | (*c*) Explain how missing data were addressed  Not applicable. |
|  |  | (*d*) *Cohort study*—If applicable, explain how loss to follow-up was addressed  *Case-control study*—If applicable, explain how matching of cases and controls was addressed  *Cross-sectional study*—If applicable, describe analytical methods taking account of sampling strategy  Included in the Materials and Methods on page 4. |
|  |  | (*e*) Describe any sensitivity analyses  Not applicable. |

Continued on next page

| Results | | |
| --- | --- | --- |
| Participants | 13* | (a) Report numbers of individuals at each stage of study—eg numbers potentially eligible, examined for eligibility, confirmed eligible, included in the study, completing follow-up, and analysed  Included in the Results on page 6. |
|  |  | (b) Give reasons for non-participation at each stage  Not applicable. |
|  |  | (c) Consider use of a flow diagram  Not required. |
| Descriptive data | 14* | (a) Give characteristics of study participants (eg demographic, clinical, social) and information on exposures and potential confounders  Included in the Results on page 6 and presented in Table 1 and S1 Table. |
|  |  | (b) Indicate number of participants with missing data for each variable of interest  Not applicable. |
|  |  | (c) *Cohort study*—Summarise follow-up time (eg, average and total amount)  Not applicable. |
| Outcome data | 15* | *Cohort study*—Report numbers of outcome events or summary measures over time |
|  |  | *Case-control study—*Report numbers in each exposure category, or summary measures of exposure |
|  |  | *Cross-sectional study—*Report numbers of outcome events or summary measures  Included in the Results on pages 6-8 and presented in Tables 1-2 and S1-S11 Tables. |
| Main results | 16 | (*a*) Give unadjusted estimates and, if applicable, confounder-adjusted estimates and their precision (eg, 95% confidence interval). Make clear which confounders were adjusted for and why they were included  Unadjusted results are included in the Results on pages 6-8 and presented in Tables 1-2 and S1-S11 Tables. |
|  |  | (*b*) Report category boundaries when continuous variables were categorized  Not applicable. |
|  |  | (*c*) If relevant, consider translating estimates of relative risk into absolute risk for a meaningful time period  Not required. |
| Other analyses | 17 | Report other analyses done—eg analyses of subgroups and interactions, and sensitivity analyses  Not applicable. |
| Discussion | | |
| Key results | 18 | Summarise key results with reference to study objectives  Included in the Discussion on pages 8-11. |
| Limitations | 19 | Discuss limitations of the study, taking into account sources of potential bias or imprecision. Discuss both direction and magnitude of any potential bias  Included in the Discussion on page 10-11 |
| Interpretation | 20 | Give a cautious overall interpretation of results considering objectives, limitations, multiplicity of analyses, results from similar studies, and other relevant evidence  Included in the Discussion on pages 8-11. |
| Generalisability | 21 | Discuss the generalisability (external validity) of the study results  Included in the Discussion on page 11. |
| Other information | | |
| Funding | 22 | Give the source of funding and the role of the funders for the present study and, if applicable, for the original study on which the present article is based  Provided. |

*Give information separately for cases and controls in case-control studies and, if applicable, for exposed and unexposed groups in cohort and cross-sectional studies.

**Note:** An Explanation and Elaboration article discusses each checklist item and gives methodological background and published examples of transparent reporting. The STROBE checklist is best used in conjunction with this article (freely available on the Web sites of PLoS Medicine at http://www.plosmedicine.org/, Annals of Internal Medicine at http://www.annals.org/, and Epidemiology at http://www.epidem.com/). Information on the STROBE Initiative is available at www.strobe-statement.org.

## SUPPLEMENTARY REFERENCES

1. Chatzis N, Hafezi F. Progression of keratoconus and efficacy of pediatric [corrected] corneal collagen cross-linking in children and adolescents. *J Refract Surg* (2012) 28:753–758. doi: 10.3928/1081597X-20121011-01

2. Gomes JAP, Tan D, Rapuano CJ, Belin MW, Ambrósio R, Guell JL, Malecaze F, Nishida K, Sangwan VS, Group of Panelists for the Global Delphi Panel of Keratoconus and Ectatic Diseases. Global consensus on keratoconus and ectatic diseases. *Cornea* (2015) 34:359–369. doi: 10.1097/ICO.0000000000000408

3. Soeters N, van der Valk R, Tahzib NG. Corneal cross-linking for treatment of progressive keratoconus in various age groups. *J Refract Surg* (2014) 30:454–460. doi: 10.3928/1081597X-20140527-03

4. Kohlhaas M, Spoerl E, Schilde T, Unger G, Wittig C, Pillunat LE. Biomechanical evidence of the distribution of cross-links in corneastreated with riboflavin and ultraviolet A light. *Journal of Cataract and Refractive Surgery* (2006) 32:279–283. doi: 10.1016/j.jcrs.2005.12.092

5. Brittingham S, Tappeiner C, Frueh BE. Corneal Cross-Linking in Keratoconus Using the Standard and Rapid Treatment Protocol: Differences in Demarcation Line and 12-Month Outcomes. *Investigative Ophthalmology & Visual Science* (2014) 55:8371–8376. doi: 10.1167/iovs.14-15444

6. Raiskup F, Spoerl E. Corneal Crosslinking with Riboflavin and Ultraviolet A. Part II. Clinical Indications and Results. *The Ocular Surface* (2013) 11:93–108. doi: 10.1016/j.jtos.2013.01.003

7. Wollensak G, Spoerl E, Seiler T. Riboflavin/ultraviolet-a-induced collagen crosslinking for the treatment of keratoconus. *Am J Ophthalmol* (2003) 135:620–627. doi: 10.1016/s0002-9394(02)02220-1

8. Wen D, Li Q, Song B, Tu R, Wang Q, O’Brart DPS, McAlinden C, Huang J. Comparison of Standard Versus Accelerated Corneal Collagen Cross-Linking for Keratoconus: A Meta-Analysis. *Invest Ophthalmol Vis Sci* (2018) 59:3920–3931. doi: 10.1167/iovs.18-24656

9. Wittig-Silva C, Chan E, Islam FMA, Wu T, Whiting M, Snibson GR. A randomized, controlled trial of corneal collagen cross-linking in progressive keratoconus: three-year results. *Ophthalmology* (2014) 121:812–821. doi: 10.1016/j.ophtha.2013.10.028

10. Ganesh S, Brar S, Patel U. Comparison of ReLEx SMILE and PRK in terms of visual and refractive outcomes for the correction of low myopia. *Int Ophthalmol* (2018) 38:1147–1154. doi: 10.1007/s10792-017-0575-6

11. Karolak JA, Vincent M, Deutsch G, Gambin T, Cogné B, Pichon O, Vetrini F, Mefford HC, Dines JN, Golden-Grant K, et al. Complex Compound Inheritance of Lethal Lung Developmental Disorders Due to Disruption of the TBX-FGF Pathway. *Am J Hum Genet* (2019) 104:213–228. doi: 10.1016/j.ajhg.2018.12.010

12. Li H. Aligning sequence reads, clone sequences and assembly contigs with BWA-MEM. (2013) doi: 10.48550/ARXIV.1303.3997

13. Tarasov A, Vilella AJ, Cuppen E, Nijman IJ, Prins P. Sambamba: fast processing of NGS alignment formats. *Bioinformatics* (2015) 31:2032–2034. doi: 10.1093/bioinformatics/btv098

14. Rimmer A, Phan H, Mathieson I, Iqbal Z, Twigg SRF, WGS500 Consortium, Wilkie AOM, McVean G, Lunter G. Integrating mapping-, assembly- and haplotype-based approaches for calling variants in clinical sequencing applications. *Nat Genet* (2014) 46:912–918. doi: 10.1038/ng.3036

15. McLaren W, Gil L, Hunt SE, Riat HS, Ritchie GRS, Thormann A, Flicek P, Cunningham F. The Ensembl Variant Effect Predictor. *Genome Biol* (2016) 17:122. doi: 10.1186/s13059-016-0974-4

16. Chen X, Schulz-Trieglaff O, Shaw R, Barnes B, Schlesinger F, Källberg M, Cox AJ, Kruglyak S, Saunders CT. Manta: rapid detection of structural variants and indels for germline and cancer sequencing applications. *Bioinformatics* (2016) 32:1220–1222. doi: 10.1093/bioinformatics/btv710

17. Klambauer G, Schwarzbauer K, Mayr A, Clevert D-A, Mitterecker A, Bodenhofer U, Hochreiter S. cn.MOPS: mixture of Poissons for discovering copy number variations in next-generation sequencing data with a low false discovery rate. *Nucleic Acids Research* (2012) 40:e69–e69. doi: 10.1093/nar/gks003

18. Zarate S, Carroll A, Mahmoud M, Krasheninina O, Jun G, Salerno WJ, Schatz MC, Boerwinkle E, Gibbs RA, Sedlazeck FJ. Parliament2: Accurate structural variant calling at scale. *GigaScience* (2020) 9:giaa145. doi: 10.1093/gigascience/giaa145

19. Steinhaus R, Robinson PN, Seelow D. FABIAN-variant: predicting the effects of DNA variants on transcription factor binding. *Nucleic Acids Research* (2022) 50:W322–W329. doi: 10.1093/nar/gkac393
